# The experiences of older individuals providing care to older dependents: A phenomenological study in Spain

**DOI:** 10.1371/journal.pone.0255600

**Published:** 2021-08-05

**Authors:** M. Alcañiz-Garrán, S. García-Sanjuán, J. D. Ramos-Pichardo, A. Sanjuán-Quiles, R. Montejano-Lozoya

**Affiliations:** 1 La Fe Nursing School, University of Valencia, Valencia, Spain; 2 Health Sciences Faculty, Nursing Department, University of Alicante, Alicante, Spain; 3 Nursing Faculty, Nursing Department, University of Huelva, Huelva, Spain; Newcastle University, UNITED KINGDOM

## Abstract

**Objective:**

Non-professional care provided in domestic settings by a family member or someone from the close environment and without a connection to a professional care service, is increasingly assumed by older people, mainly the spouses of those requiring care. The aim of this study was to describe the experience of older people providing care at home to older dependents.

**Methods:**

A qualitative study was carried out to describe and explore the experience of older people, caregivers of dependent older people in the home.

**Results:**

Four themes emerged as a result of the analysis: interpersonal relationships established in the caregivers’ immediate environment; the need and request for public and private resources; consequences of providing care during old age; and adaptation to the circumstance of being a caregiver during old age. Older people who provide home-based care, experience their situation as stressful, feel that it limits their daily life, deprives them of their freedom, and affects their interpersonal relationships and social activities.

**Discussion:**

Older caregivers learn quickly and can manage the skills issues. The volume of work is their challenge. Interpersonal relationships are altered depending on the length of time spent together and the demand for care. Public services and benefits are not adapted to the demands of caregivers or dependent persons.

## 1. Introduction

The unprecedented worldwide phenomenon of population aging has been increasing over the past few decades. This is the inevitable result of the decrease in birth rates and a progressive increase in life expectancy [1]. Global population aging is accompanied by the phenomenon of ‘old age aging’; in other words, an increase in the number of people aged 80 or over, with estimates suggesting that by 2050, the size of this sub-group will triple relative to 2017 [2]. Because of the influence of social, cultural, and economic factors, this higher life expectancy also usually translates into an increase in the time older people spend living in conditions of fragility or dependence, which leads to an increase in the need for long-term care [3]. The forecast for the number of people in need of care between 2007 and 2060 shows an increase of 115% in the European Union, and in countries such as China [4].

Care for dependent older people is organised through hospital care and primary care, globally and specifically in Spain. In spite of this, 60% of care in the European Union is provided by informal caregivers [5]. Traditionally in Spain, younger family members have provided the care of their older dependent relatives [6,7]. However, the change of family structures has considerably reduced the family support ratio in recent years [8,9]. As a result, there has been an increase in non-professional care of dependent older people by other older people, such as their spouses or someone in the immediate elderly environment [10,11]. The combination of the growing number of older people and those in need of care, and the shortage of qualified formal caregivers, pose a major challenge for the future structure and organization of long-term care [12]. In Spain, this situation is very similar to the rest of the European countries. In 2007, the Law for the Promotion of Personal Autonomy and Care for Dependent Persons (LAAD) was passed with the purpose to guarantee the welfare state. Since the implementation of the LAAD, a traditional family policy has been restructured and it is emerging in a new context of economic crisis. This means aid is not being granted and dependent persons continue to depend on state health services, which are unable to cover all their needs. Families acquire a new legal obligation to take responsibility and care for their dependents [13].

Caring in old age increases the risk of illness and worsens health self-perception, taking into account factors such as sex, old age, low income or duration of care, negatively influencing the health of older caregivers [14–16]. Previous studies have quantified caregiver burden and stress management in older caregivers [17,18], showing a large impact on their health-related quality of life. These studies refer to a wide range of ages or to the study of the burden of the older caregivers of people with specific pathologies such as dementia or cancer [19], and make specific reference to symptoms such as depression in older caregivers or loneliness. However, little is known about the experience of older caregivers (mainly spouses) who, having some degree of limitation must continue assuming the care of a relative who is also older, but dependent in all areas of life.

There is no consensus in the available literature about the benefits or disadvantages of being a senior citizen and providing nonprofessional care to an older dependent relative. While some studies show that elderly care can reduce levels of distress in the older family caregiver, by finding positive meaning in care [20,21], other studies point to a reduction in the quality of life of older family caregivers, with increased comorbidity and social isolation [22,23] and decreased perceived well-being [24].

Often, older people assume the role of caregiver with a feeling of loneliness, in which the older caregiver can suffer their own limitations related to mobility or a variety of diseases associated with their aging process [25,26]. In Spain it is estimated that only 11% of dependent older adults live and are cared for in residential homes [27]. The rest of the care is provided within the family, specifically by women who assume the tasks and the responsibility of care, without being financially paid [28]. This situation poses a major challenge in the planning of health services for older people, because it requires planning actions that target both the needs of the older dependents and their older caregivers. Specific interventions with the aim of preventing negative care-related outcomes for older caregivers need to be developed, not just at an European level [18] but as an international issue [29].

With a view to furthering the limited research performed to date on the experience of being an older people caring of an older dependent person in the home, and to address the wide range of concerns regarding how best to care for such people, the present qualitative study was undertaken in order to explore the experiences of the informal caregivers. Therefore, the aim of this paper was to describe the experience of older people caring for dependent older people in their homes.

## 2. Methods

### 2.1 Design

This was a qualitative study, performed from the descriptive phenomenological perspective of Giorgi et al. [30,31] by carrying out phenomenological interviews with an intentionally non-probabilistic sample of older care providers. These descriptive phenomenological interviews exposed the essence of how those providing care during their own old age live, feel, and deal with the situation on a daily basis. The issues discussed in these interviews were not rigid; they were fluid and evolved over the time spent with each participant. The importance of each topic and its order, or if the particular issues were addressed at all, were determined by the person interviewed. The work was reported according to the CO-REQ checklist.

### 2.2 Study sample and data collection

Older people were purposefully sampled. The inclusion criteria were: age of 70 years or more; living in the same home as the person receiving care; not receiving additional help besides the one provided by the state for the care of the dependent person; and the communicative capacity to be interviewed. In addition, the person receiving care had to meet the following criteria: age of 70 years or more; presenting some degree of dependency; and unable to go to their primary care centre autonomously.

The researchers contacted primary care nurses in the city of Valencia (Spain), who are the ones who usually monitor dependent persons at home. They were informed of the objective of the study and inclusion criteria for potential participants of the sample. Once the potential candidates were selected, the principal investigator contacted them by telephone, explaining the objective of the study and the conditions of their participation in it. The principal investigator did not know or have prior contact with the participants. Once the principal investigator, who has extensive experience in the use of qualitative methodologies and a high level of knowledge of the study topic, obtained their acceptance to participate in the study, a date and time for the interviews with the participants were set. Neither the nursing professionals nor the participants in the study received any type of economic compensation for their collaboration. To carry out the interview, an appointment at the home of the individuals who agreed to participate was scheduled according to their preferences and availability. Interviews were conducted alone between the principal investigator and the participant. All potential contacted candidates agreed to participate in the study. Data saturation was reached with interview number 13, thus a total of 13 interviews were conducted. Before beginning the interview the interviewees signed their informed consent to participation. Their subsequent narratives were audio-recorded and the principal investigator was also taking notes. Each interview lasted approximately 60 minutes. Thus, these in-depth interviews used an open, global starting point without anticipating specific topics: for example, “Tell me about your day-to-day experience of caregiving”.

In addition to the phenomenological interviews, we collected descriptive data to better understand the characteristics of the older caregivers we interviewed. These were: the degree of independence in the basic activities of daily living (BADL) measured using the Barthel Index [32], the objective burden of care measured through the Caregiver Effort Index [33], and the burden of subjective care through the Zarit Test [34].

### 2.3 Data analysis

The interviews were transcribed verbatim and the texts were analysed following the process described by Giorgi et al. [31,35] which involves five stages: identification of significant units, eidetic reduction, phenomenological reduction, essential structure of the experience, and a final and unique description of the phenomenon under study. This analysis method, designed to capture the specific description of the experience of a certain person, is characterised by identifying common (invariable) themes that, according to the narratives of older caregivers, correspond to the common essence of caring for a dependent at home during old age.

The interview transcripts were forwarded to the participants so they could read them and verify their accuracy. In the case of those who could not read them, the principal investigator read them aloud to them at home. There were no corrections from the participants, as they felt that their experiences related to the study topic had been completely captured. Then, units of meaning within the transcripts were discriminated, leading to the emergence of ‘the objective’ and ‘the given’. Eidetic reduction was subsequently used to identify the invariable aspects of the experience elements whose elimination or alteration would have substantially changed the experience. When such facets were identified, we used phenomenological reduction to obtain the ‘statements of meaning’. These specific phenomenon-related topics were significant to the participants narrating them because they constituted their personal experience; thus, these particular phases transform significant units (everyday language) into scientific language. When there was a relationship between these specific issues, common themes emerge, and these were the invariable essence of the phenomenon of caring for an older dependent at home during old age. The researchers did not use any software to perform the data analysis.

### 2.4 Ethical issues

The collaboration of the participants was voluntary and altruistic. Written informed consent was obtained from all the participants, according to the principles set out in the Declaration of Helsinki and the Belmont Report [36]. The project was approved by the Biomedical Research Ethics Committee at the La Fe University and Polytechnical Hospital (registration number 2012/0525).

## 3. Results

Sample comprised 13 older caregivers over 70 years of age, 8 women and 5 men, with a mean age of 81.7 years; 9 (69.2%) were completely independent in terms of the BADL and 4 (30.8%) were partially dependent. Regarding the objective load generated by care provision, 8 (61.5%) presented a high level of overexertion and 5 (38.4%) did not present any extra exertion. Likewise, 8 (61.5%) presented an intense subjective overload; 2 (15.3%) were subjectively overloaded, and 3 (23%) did not present any subjective overload (Table 1).

**Table 1:** Characteristics of older caregivers.

Code (Sex)	Age (years)	Barthel Index^a^	Burden of care	
			Caregiver Effort Index^b^	Zarit test^c^
E1—Woman	85	Independent (100 points)	High level of overexertion (11 points)	Intense overload (93 points)
E2—Man	75	Mild dependence (70 points)	No overexertion (3 points)	No overload (46 points)
E3—Woman	75	Independent (100 points)	High level of overexertion (12 points)	Intense overload (77 points)
E4—Man	89	Independent (100 points)	No overexertion (5 points)	No overload (46 points)
E5—Man	85	Independent (100 points)	High level of overexertion (13 points)	Intense overload (69 points)
E6—Woman	71	Independent (100 points)	No overexertion (6 points)	Overload (55 points)
E7—Man	84	Independent (100 points)	High level of overexertion (10 points)	Intense overload (65 points)
E8—Woman	85	Independent (100 points)	High level of overexertion (11 points)	Intense overload (61 points)
E9—Woman	82	Mild dependence (70 points)	No overexertion (6 points)	Intense overload (68 points)
E10—Woman	80	Mild dependence (95 points)	High level of overexertion (12 points)	Intense overload (57 points)
E11—Man	84	Independent (100 points)	No overexertion (6 points)	No overload (34 points)
E12—Woman	81	Independent (100 points)	High level of overexertion (10 points)	Intense overload (73 points)
E13—Woman	86	Mild dependence (85 points)	High level of overexertion (12 points)	Overload (54 points)

^a^ Basic Activities of Daily Life Scale. Scoring: 100 points = independent; > 60 points = mild dependence; 40–55 points = moderate dependence; 20–35 points = serious dependence; < 20 points = total dependence.

^b^ Used to assess the objective overload generated by providing care. Scoring: ≥ 7 points = high level of overexertion.

^c^ Used to assess the subjective overload generated by care provision. Scoring: < 47 points = no overload; 47–55 points = overload; > 55 points = intense overload.

Four common themes emerged from the interview analysis: 1) interpersonal relationships established in the caregivers’ immediate environment; 2) the need and request for public and private resources; 3) consequences of providing care during old age; and 4) adaptation to the circumstance of being a caregiver during old age (Table 2).

**Table 2:** Summary: Analysis of the experience of providing care to older dependents at home during old age.

COMMON THEMES		SPECIFIC TOPICS
**Interpersonal relationships established in the caregivers’ immediate environment**	• **The primary care team**	• Relationship with the family doctor: impersonal; managed by telephone. • Home visits by nurses: short, few, and routine. • The older caregiver is considered a ‘resource’ rather than as a ‘client’. • Mistreatment classified as: negligence; paternalism.
	• **Children**	• Relationship: short visits, contact by phone. • Help with transfers, mobilisations, or accompaniment to the doctor. • Children show little empathy. • No reciprocity of care.
	• **Grandchildren**	• Relationship influenced by certain variables: activities; imitating their parents.
	• Older **dependent person**	• Context of the couple: their cohabitation history. • Disease impact. • High demand for attention.
	• **Neighbours**	• Positive relationship based on years of residence in close proximity. • Neighbourhood conflicts: negative consequences.
**The need and request for public and private resources**	• **Public administration: increasingly difficult**	• Political decisions; economic breakdown • Lack of public information: social alarm. • Retirement pension: the influence of sex. • Incoherence in administration: unrealistic assessments. • Municipal social services: “a disaster”.
	• **The search for well-being**	• Gaining access to public or private services and benefits; their optimisation.
**Consequences of providing care during old age**	• **Sex determines the task of caring**	• ‘Delegate care’ or ‘sliding care standards’
	• **Day-to-day care**	• Deteriorating health. • Personal abandonment. • Loss of opportunities. • Family problems. • Loss of wealth. • Mistreatment classified as: psychological; abandonment.
**Adaptation to the circumstance of being a caregiver during old age**	• **Where the care is provided**	• Adaptation of the surrounding environment. • Reasons for providing care at home.
	• **Learning every day**	• Trial and error. • Observing nurses. • Following nurses’ verbal and/or written instructions. • Many years’ personal experience in care provision.
	• **Life experience**	• Capacity for resilience. • Religion. • Respite tasks: always with the help of another person.

### 3.1 Interpersonal relationships established in the caregivers’ immediate environment

During interviews participants talked about the interpersonal relationships they experienced from three different perspectives: their relationships with 1) health professionals, mainly primary care; 2) relatives and close friends; and 3) the person they were caring for.

According to their narratives, their relationship with the primary care team in general, and especially with doctors, became increasingly less direct and close. In addition, they described their perception of paternalistic attitudes from these professionals. Home visits, usually by nurses, were perceived as routine, short, and infrequent.

*The family doctor has only come twice, because I asked them to. Twice in four years. They treat her from there, by phone (…) E5*.

*(…) The nurse came and as always, took our blood pressure. She gave us the vaccine and left so fast that I didn’t remember to tell her about the sores, and I didn’t tell her anything, or that he has another one on this side [points to {his} left side] (…) E10*.

The relationships that older caregivers have with their family members depend on their relatives’ stage of life. The participants stated that both their children and grandchildren were progressively distancing themselves, so that they had less and less time to participate in and share the tasks of caregiving. In fact, in this sense, some of the participants expressed a certain feeling of not being reciprocated for the care they had given their relatives in the past.

*(…) {my} children have so many activities with work, they don’t have enough time. {The} grandchildren neither, because they’re doing, I don’t know what, if not English, then gymnastics, or I don’t know what. We don’t see them. After having raised them all. E3*

The type of relationship between the caregiver and the person they cared for prior to the care situation also emerged in the narratives as an important element in their current relationship. When the past relationship and experience of cohabitation were valued positively by the caregiver, and the relationship remembered as one of respect, trust, and love, the task of providing care was more easily assumed. However, where the previous relationship was perceived and remembered as having been asymmetric, where the older dependent person had dominated and communication was impaired, assuming the care tasks was more difficult.

*You can’t talk to him [crying], you can’t… so all day I don’t say anything to him, I don’t speak to him, only well, badly [crying], there is no relationship (…). It’s that he’s got a very fast temper… he has a fit of anger and everything you say to him, everything is NO. He rejects everything. So many years and always fighting, {it was} always like that, always like that, and me putting up with it. (…) Oh! what mistakes we make in life. E1*

Most of the narratives described the people dependent on the participants’ care, especially the main factors limiting their mobility and the cognitive impairment preventing their verbal communication or other interactions with the environment. These pathologies had been evolving for many years and created a high care demand.

*(…) She’s been {like this for} six or seven years, she can’t walk anymore, nothing, not at all, or stand up, nothing, always in the wheelchair, (…) her head goes, she’s gone deaf, and her sight is failing, she has a tube for eating and to pee. I’m so fed up with all the years we’ve been like this. E4*

*(…) since the first operation, it’s been about 14 or 15 years (…) now she doesn’t hear, you have to shout at her, and even then, she doesn’t understand much. They say she has Alzheimer’s (…) we’re doing tests, {it’s} a disaster (…). E7*

The descriptions of relationships with neighbours are often very positive. These are sometimes described as very close relationships of friendship and trust in which the neighbour even becomes a source of support and a resource for helping with the daily care tasks.

*(…) the neighbour in front comes over, and since she’s going to buy something, I tell her, “Well, bring this for me from the pharmacy, or something else”, or “come with me to go shopping and keep me company”, because for me, going alone, I’m very bad {fragile}, I can’t (…) I can’t lift as I could before, I can’t move anything by myself and she helps me. E10*

### 3.2 The need and request for public and private resources

Participants usually described economic problems related to an imbalance between their income and their needs. Some of the interviewees stated that they did not receive their own pension (and so they depended on the pension of the person they cared for) because of situations experienced during their working life, which affected their retirement entitlements.

*My husband didn’t want me to make {social security} contributions while we worked together in the shop. All his life he said: “you, why should you have to pay anything” (…), {but} now I would {have} receive{d} a pension and so look, my work was for nothing… E6*

Some also mentioned a perception of incoherence between the assessment of public administrations about the status of the person they care for and the benefits they receive. The interviewees considered the latter scarce and poorly adjusted to the real need for support.

*First, they send me to one, and then {they} send {me} to another [dependence-law evaluator] and, of course, when they saw him [her husband], they gave him the highest grade. Of course, a person who does nothing, totally dependent, and now it’s going to be three years (…) The social worker offered me a professional carer for an hour. Right, tell me, a woman for one hour, sincerely, will not solve anything, and on top of that I {would have} had to pay {her} €10. And I said, “I don’t need these favours”. And so… does this seem fair to you? I don’t expect anything anymore, I don’t expect any help because, they haven’t even bothered with us since then”. E8*

One of the interviewees commented that when the social security benefit they received became consistent with their needs, their own health and that of the person they cared for both improved.

*They decided that our health was quite poor, and they granted us {the help where} they bring food at home. At least at lunchtime, we eat, and we both now have better health. E2*

### 3.3 Consequences of providing care during old age

Older caregivers stated that they performed all the domestic tasks, as well as those related to the care, and contracted domestic help services based on their economic means. Some of them said that they had hired young people to help with the task of transferring the person receiving care and other tasks related to their workload, because they themselves could no longer cope with these tasks.

*A girl comes once a week or every 15 days, depending on the money {situation} (…) as he cannot move, I {have to} handle everything. E3*

*(…) I pay a boy who comes two hours every day and puts my husband to bed, because I can’t, with this [pointing to her husband] I can’t. E13*

For their part, older male caregivers mentioned that they usually delegate basic care to female relatives or to domestic help services, which they usually hire from Monday to Friday, meaning that at the weekend they do not have this resource.

*I pay a helper. She is here all morning for everything that has to be done at home and to take care of my wife. She gets her up, feeds her, controls her tube, her treatments, everything (…). E4*

Participants said that long-term care at home caused them stress, greater physical and emotional exhaustion, abandonment of their personal projects, and resulted in family conflicts and wealth loss, sometimes leading the older caregiver to abandon some of their care tasks.

*Sometimes I have tantrums when I feel low, which happens to me a lot. I’ve been bad for two or three days now, I don’t get excited about anything, I’m joyless (…) Look at my hair [pointing out its lack of colour]. I can’t go to the hairdresser. My head hurts all the time… I don’t feel like doing anything, I’ve lost my appetite. Terrible, a disaster, I’m a disaster. E8*

*(…) the family relationship is very tense, the older one [daughter] is single, she would also like to live her life differently, many days she’s aggressive. I, because she pressures me, well, I get angry, and when I get angry, we argue… and that’s it (…) this is the atmosphere of a house where there’s a sick person like this (…) it changes everything, from top to bottom. E5*

*(…) I bought one of those hospital beds (…) E10*

### 3.4 Adaptation to the circumstance of being a caregiver during old age

Participants said they had had to make alterations to the organisation of spaces in their homes, depending on the needs of the dependent person and the care tasks the caregiver had to carry out.

*Before, the house was more organized, but now, if I put my bag away, then I can’t find the money or the keys. If I put these away [pointing to some packs of {adult} diapers], my daughters can’t see them and they start {asking} “Mum, where’s this or that?” In the end, I put everything here [in the dining room dresser], and there aren’t any problems, everything is at hand. E3*

Narratives show that the decision to provide home care is based on a commitment to the older dependent, in the sense that the person receiving care had expressed their desire not to start living in a long-term residency center. The female older caregivers assume the role of care giving naturally. To them, they are the ones who have to take care of their spouses because “that’s the way it’s always been”. In addition, they also consider the distance to the residential center, negative experiences, and their distrust of the professionals working in these centers, stating that at home they can better control their own time and pace of life.

*(…) that’ll be when I can’t attend to him anymore, while I {still} can, that won’t happen [crying]. She makes me talk and everything, but I take care of him. E12*

*They told us to bring her home or take him to the long-stay hospital. It was like abandoning him, so far away. I thought: “no, there they’ll let him deteriorate, no, no, I’ll take her, I prefer having him at home. If something has to happen, let it happen in front of me” (…) those [professionals] get used to it, if the relatives aren’t there it seems like nothing gets done… E5*

The narratives of participants also showed that they learn about how to carry out the care tasks through trial and error, observation, experience, and by following nurses’ verbal or written instructions. In any case, the caregivers included in this study stated that the care tasks became increasingly complex as they themselves got older or as their own health had declined; they felt that they were not prepared for this work and showed signs of insecurity and doubts about what they had done in certain situations.

*(…) I give her insulin and something else that you put {inject} in{to} the belly. I don’t know if you know, I don’t know if I gave her too much, but that’s when she started to get really unwell. In short, a disaster. (…) E9*

*I treat him every day. I learned by watching. At first the nurses told me how to do it, “look, first wash him with this water”, I have a little bottle {of it} there, by the way, I don’t have any left. “I wash him and then put this ointment on him”. Whatever it is, I put it on. E13*

According to the narrations, experience, patience, knowing each other and being able to anticipate certain situations, love for the other, humour, religious beliefs, and/or emotional suppression are some of the strategies used to face these adversities. Going out to go shopping every day, to the doctor, or to the pharmacy are the moments these caregivers identified as distractions, which allowed them to maintain social relationships.

*(…) the experience comes from afar; I watched a lot of things. The family doctor tells me that I could be a nurse. E9*

*I have a lot of patience, a lot of patience. I think I’ve adapted, after living through so much and so many years (…). E8*

The narratives showed that older caregivers were mostly satisfied with the care they provided. However, some older female caregivers said that they had always performed this role, without it being recognized or valued.

*At first, {there was} a problem, what do I buy? What do I do? Now, I’m fending for myself. I’m learning. This {thing} is useful to me, this {one} isn’t useful to me. This {experience} is like that. I’m learning. For me it’s an achievement. (…). E5*

*I took care of my mother, (…) when my mother died, I spent three years looking after my father, who was blind. Then {looking after} my own family, and now this (…) always the same, {it’s} a pity. E1*

## 4. Discussion

This study reveals the stories of older caregiver who care for dependent older people. This care is complex and requires excellent skills and abilities to meet the needs of sick family members and manage their own lives. The results of this study reveal the conditions and challenges faced by these caregivers, both personally, the mobilisation of resources and the impact on their quality of life.

Participants felt that their relationship with health professionals was insufficient and too routine. In this line, some studies have also indicated the inadequacy of the professional care received by caregivers in terms of the number of visits, the time spent during each visit, and the criteria used to decide if a primary care worker home visit is necessary [37]. Even though the literature indicates that the relationship of trust caregivers have with the primary care team is decisive in whether they can cope and adapt to the care situation [9,11,18], professional interventions usually focus only on the disease and the person affected by it, and rarely include the older caregiver [38].

Our findings suggest that older care providers consider caregiving as an act of reciprocity; they internalise the role of primary caregiver and avoid seeking help, which generates feelings of ambivalence in them: they feel that they are stuck between their caregiving obligations, exhaustion, and conflicts of interests with their descendants [39,40]. They also feel obliged to take care of the dependent person at home, especially if that person had expressed a desire not to be institutionalised in long-term residential care. This circumstance is very frequent in Southern European countries such as Spain, where family ties are deeply rooted and there is a strong feeling of family [41].

Aging in place has implications for older people in terms of their sense of connection, identity, security, and familiarity [42], and so they often prefer their own home over other old age residential centres [43]. However, support policies for aging in place focus on the physical infrastructure comprising the home and pays very little attention to the caregivers, relatives, or friends required to help elderly individuals stay in their homes [44]. This situation was also reflected in our findings, which showed how older caregivers feel that they receive little or inadequate aid financial or personal assistance for caregiving tasks. According to Calvó-Perxas et al., (2018) [18], the self-perception of health among older women providing homecare is greater if they have access to respite resources and contact with other people, whether for social or learning purposes.

Some of the older male caregivers in our study do not provide all the attention on their own, but instead "delegate" certain basic care and household chores to private domestic assistance services and / or female relatives. Traditionally, it is women who have taken care of dependent family members [45–47], showing a level of unmet needs among older women [16,25], since the provision of care supposes an objective and subjective overload for older female caregivers [11,16,40]. Sex influences the way that the satisfaction of providing care is perceived. While for men, care is a source of personal enrichment, for women, care becomes something they must do to demonstrate the love they feel for the person they care for, which turns the caregiving task into something much more demanding and less satisfying. Sometimes the caregiver, especially women, assume this role because they feel it is expected of them, particularly in countries in which family-based care remains important [18].

Like other studies, our results show that providing care during old age generates stress, loss of wealth, increases physical and emotional wear and tear, and can lead to social isolation. In addition, older caregivers harbour feelings of dissatisfaction and/or unhappiness, more often suffer situations of family conflict, and perceive a loss of opportunities because they have had to dedicate themselves to care in a stage of their life they had believed would have been devoted to rest and calmness [9,29].

Factors such as excessive demands by the person receiving care, as well as factors related to the older caregiver such as being a woman, a spouse, objective and/or subjective overload, not understanding the demands or evolution of the care receiver’s disease, and emotional and social isolation, increase the risk that older caregivers could behave abusively, and may even lead to the abandonment of care tasks [48]. Therefore, establishing policies to help older caregivers which go beyond merely targeting their physical environment, is of utmost importance to improve not only their welfare, but also that the person being cared for. Ensuring the provision of quality care could also potentially reduce the long-term use of health resources for instance, by reducing hospitalisation frequencies.

### 4.1 Limitations

The analysed data in this study reflect on the lived experiences of a specific group of older people who care for dependent older people in their home. Understanding this from an experiential learning perspective, far from global generalizations, provides researchers and professional workforce with useful information to make concrete decisions about the issues identified as weaknesses of the Health Systems and older caregivers perceived needs.

## 5. Conclusions

The findings of the present study contribute to an increase in knowledge and comprehension of older people who care for dependent older people in their home. Care has a temporal dimension, in which the caregiver must adjust and acquire skills, as their family member’s condition progresses. The decontextualization, on the part of the healthcare system, of the family caregiver makes them feel invisible to that very same system. The lack of information and support for caregivers would appear to go unnoticed by healthcare providers. Consequently, it would be recommendable that healthcare professionals could incorporate caregivers’ experience, along with the dependent people caring process by implementing inclusive politics.

## Supporting information

S1 File (DOCX)
