# Supplementary material for: The experiences of older individuals providing care to older dependents: A phenomenological study in Spain
Source: PLoS One. 2021 Aug 5;16(8):e0255600. doi: 10.1371/journal.pone.0255600 (PMC8341577; doi:10.1371/journal.pone.0255600)
Supplement: S1 File — (DOCX) [file pone.0255600.s001.docx]

**Transcripts of interviews with participants.**

**Pseudonym of the interviewer: Mar**

**Code interviewees: E1;E2;E3;....**

**E1**

Mar. What have you done for a living? Have you worked outside the home? 1`25

E1. I have worked a lot. My parents were from the countryside, from the town of XXX.

Mar. Teruel.

E1. I have worked a lot, because we were nine siblings and I was the youngest of them all. They have all passed away. I also worked in the fields, with beets. In the summer, the potatoes to pick them, to plant them..... in the field I worked a lot.

Mar. And at that time... the conditions and the climate in Teruel, it was very cold.

E1: Yes, very cold, but you get used to everything.

Mar. Very cold and very hot.

E1. Yes, yes, but the cold is stronger than the heat.

Mar. Do you have any illnesses?

E1. No, I have some cholesterol but now I have to have a cholesterol test because I haven't had one for a year, the last time I didn't have anything at all. The doctor told me: "E1, you look like an 18 year old girl", I had no cholesterol, no sugar. Everything was fine. So now I have to go for a test because it's been a year.

Mar. Of course, it is convenient to have a check-up every year, that is important.

E1. Now I have high blood pressure. 3'02

Mar. Do you take medication?

E1. Yes, I take a little pill at night.

Mar. Well, do you walk? Do you go for walks?

E1. Yes, I go for a walk.

Mar. What about salt, how do you take it, do you take it out of your meals...?

E1. Well, we eat half salt, we eat very little salt at meals 3'32

Mar E1, I would like you to tell me what is your experience of taking care of other people?

E1. Well, I took care of my mother a lot, because she had asthma, she died of asthma. I stayed two months sitting in bed with my mother, she leaned on my shoulder and I sat in bed, so I spent two nights in a row, two days in a row. Because her asthma would not go away. Before, there was nothing to remove the asthma, now when the oxygen was put in, they no longer suffered, but there was no oxygen. My father became blind, my mother died and I stayed with my father for three years. Later my father came here to Valencia, he had family, siblings and family, but my father did not want to be here, he was so old that he did not want to be here. My sister came and took him to the town and I, then I went to the houses as a seamstress 4'52 and to clean silver or to iron, I worked in what I could, then, they told me to go abroad and I went. I have been in London for about 15 years.

Mar. What kind of work did you do?

E1: Oh, as a maid. There you only worked as a maid. Until you had 4 years off, you couldn't work there as anything else 5'30. Of course, they also went to study for 3 months and after 3 months they had to come back, or, they also renewed their contracts and continued. Yes, I have worked a lot in XXX. I have worked in the hospital, in XXX, which is in the center, because I saw that I earned more... The girls would tell me, "Oh, come to my hospital because there". In the first one I was in, we had two shifts, in the morning and in the afternoon, of course these girls said "oh E1", because you immediately became Spanish friends, "well, in the one I'm in we start at 7 in the morning and at 2 o'clock we are free", so I went there at 6:26 because in the afternoon I was going to work somewhere else, so I took a house for 4 hours.

Mar. And what did you do?

E1. Cleaning. After this house, I don't know why I lost it, I went back to XXX and stayed for a month. Then when I came back, I did the same, I was in the hospital, because it was very good there in the hospitals. I had the residence, the nurses downstairs on the second floor and the maids, upstairs on the second or third floor. It was a very good residence for the girls, with their television, you came down at night and they gave you a glass of milk with cookies, we were very well, they gave you food, all this paid, when they paid your salary, they discounted it, they discounted the room and the food. That's where it was better, in the hospitals. In the hotels, they made you work a lot, they made you lift beds, in the end .... the work, when they left, they made you clean the room, this was after the 4 hours that you worked in a house from 6 to 10 hours, then all this [in the hotel]. That is to say, there were times when I had three jobs, because you know, as I was alone, [in Spain] with no shelter, I said to myself "what am I doing here, I'm not doing anything here", I didn't make a penny, because I also had to help out, right? And that's why I left, I left with the desire to say "I'm going to work to buy an apartment in XXX" 8'42 With the illusion of coming to XXX and everything I bought, everything for XXX, everything, everything, that's been my life, work, work, work....

Sea. And he has succeeded.

E1. Yes

Mar. How did you meet your husband?

E1. Ah well, look. Two of my sisters died while I was in XXX, I couldn't come because I lost my job and then what do you do, I didn't have anyone to go to their house, nothing, and I couldn't go to a hotel either. In a hotel you can't, and 9'41, I met him in those conditions, some girls introduced me to him, "he's a good person, that and the other" and in six months I got married [silence] and here I am....

Mar. Is this the first time you have been taking care of him?

E1. Yes it is the first time, he has never been bad and neither have I.

Mar. You haven't had to take care of... except for the time you took care of your father and your mother, because you did take care of your mother?

E1. Yes, but that was before I went to England. I was very young. When my mother died, I was 32 years old, I was at home and when I was 3 years old my father died, a month after I was in England my father died.

Mar. A very hard situation.

E1. Look and nothing, well, that's it. You get married without love because you get married when you are 47 years old and you get married just to get married... It was 73 when I married XXX I was 47 years old and I got married just to get married.

Mar. And how was the experience?

E1. The experience as a husband? Well [silence], I don't know much about it. ....

Mar. Now, how do you live the experience of having to take care of him? 11'55

E1. Now, I am a little fed up because he becomes unbearable, at times unbearable, for nothing [nervous]... yes .... [silence].

Mar. It costs you ....... do you have any help?

E1. No... [teary eyes], I do it all myself. Well, yes [tearful, nervous], I've had bad luck. Bad, to say, "he's a bad person" no, but... he has a very bad temper, he has a very bad temper [gestures with hands and face] Last night..... Well.... If you start counting.... [cries and shuts up]

Mar. Well, it doesn't matter.......

E1. It's not worth it...... [She cries].

Mar. You can be quiet about it........

E1. It's just that she has a very strong prompt, I'm different...we are like night and day, you know [takes a deep breath and seems calmer]...and that's my life.

Mar. It's just that they are difficult situations to deal with....

E1. Anything he doesn't like..... Every day, I put him in bed, I open it for him, every day.... I warm it up for him...... [He cries], I put on his shoes, I dress him, I put on his diaper..... Everything, he doesn't notice anything.......

Mar. He doesn't cooperate?

He1. ¡¡¡¡ He ¡!!!!! No, no way, he, now, he could make a coffee with milk, but nothing.... Look, I, in the morning I make him one, at breakfast time another one, after lunch he is already asking for another coffee with milk, at snack time he wants another one, then I will make a tea, at night before going to bed another coffee with milk and he sits all day long... He cannot get up and say "I am going to make a coffee with milk".... He has the kitchen nearby, with a ceramic hob and we have a microwave, just put it in there and that's it, no more.... You have to give him everything done, because, look, he has been a waiter, his job has always been as a waiter...Nobody can come to my house...When my family comes, my family says "these are not visiting hours" so loud "because they are not visiting hours" and I tell him "but they come for me", "to see his aunt, to see me". In the end, I had to tell him "this is my house" I had to get strong.... "this is my house and in my house I'm in charge". He did the same thing to my nephew [he shows me a photo], his name is Luis and his brother XXX who came with his wife, after going to a funeral of a relative, I told them "come home for tea", since it was a little late, it didn't suit him. He started to shout and tell XXX "I don't love you, who I love is your brother"... in short, some things...... that my nephews, do not mess with anyone and are beautiful people, not because they are my nephews, but it's true.... Well, nothing 15'56 and so, so, so, so with everyone with all my family, so, another nephew that I have.... slapped him, another one... well, how could I put up with so much?

Mar. Do you go out? Do you talk to your family?

E1. Yes, yes. On Sunday I went with my family to XXX, they have a chalet, they told me "Aunt come, aunt come, you haven't been out for a long time" and I went with them. I left the food for them. I haven't been out since October, in the hospital, but he doesn't notice anything.....

Mar. What happened to XXX?

E1. Well, look, he had a thrombus in his groin and in his leg and his toes turned black, of course, the toes had to be cut off. The doctor said "we have been very lucky because we have saved his leg", he has undergone surgery for all that. Now he is doing well, eh, he is almost completely cured. Well, now in the other leg he also has a thrombus, being in the hospital, the doctor told me "before leaving the hospital we have to look at the other leg" he also has something and we have to operate. He says "I am not going to operate again, I don't want to go through the same thing" all furious, I tell him "do what you want, it's up to you, so everything is up to you".

Mar. If he doesn't want to...

E1. He will have to want to, because if not he will go through what happened to the other leg or worse, so we will see, what he decides... When we went for the check-up at the hospital we were with the doctor and he told him "that the other leg had to be operated" and he did not say anything, they made him sign the papers of the anesthesiologist and others .....

Mar. Informed consent?

E1. Yes, that is, he signed, but now, he says he is not going to have surgery, well, he can do whatever he wants....Su first of all, everything, everything, everything you tell him, everything is NO, he rejects everything. Later, maybe she will change, but the first answer is NO.

Mar. And you E1, how do you feel, do you have help?

E1. No, I don't have any help.

Mar. And how do you cope? Because there will be times when it will be very hard

E1. I'm having a hard time [nervous and looking at the door, she thinks her husband is eavesdropping and she is afraid. She cries]

Mar. She is having a hard time [She cries] E1.

E1. And what am I going to do?

Mar. The truth is that it is difficult, at the moment with him being like this, it is a very important burden for you, can't you count on the nephews? ......

E1. No, because each one has his work, you know. I have a niece who has two girls, her husband is also disabled for years and years and I can't count on her for anything either. Another one, she is a widow with a very delicate son, she has to help her son because he is unemployed. She was working in La Fe for 33 years and before that for 2 years in a factory in XXX, I know this because she is retired too, of course, we are all very old because I am already very old and they are the daughters of my sisters and brothers, I have many nephews and nieces. They love me very much. I am very fond of all of them, the family has always gotten along very well. In the village, in my parents' house, they had two houses, my sisters lived in one of them, because in the war they killed a brother and the husband of another one, he left her with four children, so of course, we all went to our grandmother's house and we all grew up together. We have all been very close.

Mar. What problem did you have for not having children?

E1. I got married when I was very old [silence] and he didn't want to either, so I told him "look, let's get treatment with pills". I also worked in a boutique and there with the girls we talked a lot and some of them commented that they couldn't have children and that they had gone to this and had stayed in this state and I told XXX "let's go see ....." but he didn't want to. If I had known that I was not going to have children, I would not have married, because I love children, as many children as there have always been at home. I have always liked them. Well, this is my life.

Mar. The truth is that you have had a very busy life, because between what you have worked for .......

E1: Oh yes, I have worked a lot. I have traveled all the meters of XXX. 23'05 from here to there... that I arrive that I don't arrive running.

Mar. What is the difference with what you are doing now, taking care of XXX? You, in your feelings? In your way of seeing is ....?

E1. Well, I have loved and treated my sick members of my family with affection, I have felt their illnesses so much... yes..... Now I am taking better care of him than he deserves... because he doesn't deserve what I do to him [cries inconsolably. Very tense situation].

Mar. You are suffering a lot E1. You are suffering a lot now............

E1. Oh my God.... [cries] I put up with him all day long [cries with grief] Mar.

Mar. I beg your pardon?

E1. Let's see if this goes away a little bit....

Mar. Of course it will go away......

E1. [Sighs]...because he has such a bad temper...you can't talk to him [cries] you can't...because he throws everything away...so all day long I don't say anything to him, I don't talk to him, nothing....solo bien.....mal... [cries]. I can't have a conversation with him, or anything....... [Cries].

Mar. Will he have problems because I came here now?

E1. I don't think so, I don't know. He asked me why I came and I told him "I don't know" [looks at the door]. [looks towards the door].

Mar. I don't know if another day, I would have to talk to you, I don't know, but I ask you to tell me in all confidence that if you are going to have any problems, I am not coming home. .....

E1. No, I don't think so. What I do think is.... That where it hasn't changed already....then it doesn't change, nor will it change..........it's like that forever, like that.

Mar. I don't want you to.... [I look for a handkerchief to give her, she doesn't stop crying.]

E1. Last night... [She cries] I have here some handkerchief....

Mar. I have handkerchiefs...

E1. Yes, I have some here too...yes, I do....thank you. Last night, I put the diaper on him and I told him "you have to do these things yourself", well, no.....he throws himself on the bed and shouts "I've taken the blanket, I've taken my clothes", but shouting, "but boy...you know that every day I put your clothes on like this", every day the same thing. He throws himself on the bed half sideways, with all his weight....and then he drops on top of it...if you already know....it's the everyday thing. I give him a piece of diaper because he has a handkerchief to wipe himself...I give it to him and he says "no, I don't want it" I pick it up and throw it on the table...because it fell on the floor...he went, he went, like crazy.... He said "oh please, help, help, help" but like a madman, but you think that asking for help "help, help, help" but like a madman, I told him "that's the most pious place, the floor", he starts asking "help, help, I'm very bad, I'm very bad" but you think so.

Mar. Does he exaggerate in order to keep you there?

E1. Well, yes, I have to put up with him a lot, a lot.

Mar. And now, for example, this afternoon... you will be here all afternoon?

E1. Every day here

Mar. Don't you go out?

E1. There at 6:30 or 7 o'clock in the evening I go out for a while, I go for a walk around here and that's it, or in the mornings, I go shopping...

Mar. Do you socialize with your neighbors?

E1. No, I don't have relations with them. I have a niece upstairs and a nephew downstairs.

Mar. Ah on the same farm

E1. Yes 28'28, but I also dispatch him, treating him like that...And the boy doesn't go upstairs, he's home alone too. Upstairs, the niece has Alzheimer's and she's with her son and daughter-in-law, and, sometimes, they come down and we have tea...and it bothers them too, then.......

Mar. The truth is that it is difficult E1.........

E1. So many years, so many years and always fighting, always fighting, always like this, but for nothing, always like this. How many times have I told him "go away, go away from my house, go away from my house"...but what, where is he going to go?

Mar. Now is when he needs you the most....

E1. No, now and before that, it has always been like that, since we have come here to XXX. He came here to XXX. Because, I came, and he stayed working. He worked there for 6 months, he came, he was here for 6 or 8 months, without working, he ran out of money right away.... and I told him "you can't stay like that, I have to look for an apartment there and I will go there". But I wasn't leaving there for ...... I worked hard enough to make my own home. I did it so that he could stay there, but he didn't stay there for 2 years and he got tired of being there, he came and he didn't work anymore, he signed up for unemployment .......

Mar. Does he have a pension?

E1: Yes, he has a pension, but he gives me 400 euros a month, and that's all, he doesn't pay water, electricity, neither this nor that, nothing at all, no contribution, nothing, everything is free.

Mar. There are things in life that we do....................

E1. There are mistakes [silence], because I have been very good with all the people, all those who have known me, all of them, have loved me...... and I have gone to catch this firecracker who is not worth it [silence].

Mar. And now I have to take care of him

E1. You see. I, if they have to operate again, if I can, they should take him to the hospital, leave him there until he is cured. Because I'm too old to take care of him. If there is a hospital that takes care of him until he is cured, right?....hasta que también....

Mar. Yes. Who comes to cure him? XXX or XXX?

E1. XXX is coming to cure him, no, no, XXX is coming, he will come on Friday.

Mar. You should talk it over with him, because XXX really needs care and you are too old to take care of him.

E1. No, no, of course not.

Mar. It's just that you can't take him up, take him down, put the package on him, you can't handle him, it would be interesting to talk it over with XXX.

E1. With XXX

Mar. Of course, if the intervention takes place, then things are valued and you can see, with the experience that you have had now...

E1. I just can't do it anymore, I can't do it anymore...cleaning the house, shopping, cooking, washing up and taking care of him, he is there all day long .... "give me this", "give me that", "give me the other" ...... He doesn't get up at all, he doesn't move 32'58

Mar. At your service.... Is he disciplined for what the doctor tells him? If he tells him that he has to walk....

E1. No, no, he doesn't walk, he goes.... The doctor tells him he has to walk, he has to go out in the street.... but, no, he goes. He went to get a pair of glasses and called a cab, took him and brought him back...he went out. Or he went down to get a haircut, he has it here under the farm, in the corner, there he has the hairdresser's...He cut his hair there, that is, he doesn't walk, nothing....he has never walked...that is why this has happened to him.... because he is always in front of the drawer, he is always in front of the TV and sentao and that is why he has had bad circulation, because he has no sugar, well that...34 He has no sugar, and it is because of bad circulation, he has not worked for many years, for many years, since he has been here, because...he was in England...when I came here, he came for 6 or 8 months, he left for 4 or 5, he was there for another 4 or 5 months and.... .then he came here definitively because .... he stayed 2 years without receiving any unemployment benefits, until he turned 65 years old, he spent 10 years here without receiving any kind of unemployment benefits, and without working anywhere...

Mar. And you paid for everything?

E1. Me.... working. Because I started to work..... I don't know if you know San Francisco Javier, which is where it is now....eso that is in front of XXX, the department, because that was a school for children... did you know it, a school for children, there were 200 children and more, and I was there in the kitchen.

Mar. I did not know the school......

E1. Yes, yes, well, I worked there for at least 6 years and then we went to XXX, with the children, with 8 kids and 3 nuns and then they made another change and I went to XXX in XXX, well there, there was another convent of nuns that also had some of these kids from .... what are they called .... I don't remember now.... de.... collected, because there I was also there for 3 years, yes, yes, I have been working here until I retired, and he has been here eating the soup.... that is why now, when this happens, if he had moved, now, he would not have 36'30, but look, what can we do, we all have our turn.......

Mar. Well, there are things that little by little can be changed, because now there is another intervention.......

E1. That's what I say, would you like a cup of tea?

Mar. No thanks because I came here so nervous, now I am calmer.

E1. Why, why didn't you know the place?

Mar. Of course, it's the first time I talk to a person about such intimate matters.

E1. I don't know about this. ....

Mar. I guarantee it to you, besides, nobody has to hear this. Only me and my director are going to listen to it, she has to help me analyze these interviews.

E1. Yes, yes, yes, yes, that's all.

Mar. Do you want to tell me anything else?

E1. Yes, I have nothing to tell you, I have only been working and working.

Mar. Do you feel a bit of relief talking to me?

E1. Well, I have relieved myself a little bit.

Mar. A lot of times talking to another person relieves us. ..........

E1. Yes, but you know what happens...I don't talk much, I'm very quiet, I don't talk much, I know my faults too, I'm not very talkative, but okay.

Mar. Do you have any problem in seeing me if I need to talk to you again?..I am going to call you anyway, to see how everything is going [laughs]....

E1. What is your name?

Mar. Mª del Mar

E1. Yes, Mª del Mar, you told me the other day.

Mar. Yes, when I see all this if I need her I will call her.

E1. Okay.

**E2**

Mar. How old are you?

E2. 75. Sex? Male.

Mar. Marital status? married

E2. Well...in those days [in a sardonic way], it was not common to remarry....

Mar. Well, but anyway, nowadays people get married too...eh [laughs], do they have children?

E2. No

Mar. Do you own the house?

E2. Yes, owned in a limited partnership.

Mar. E2, what have you worked on?

E2. Uff .....

Mar. A lot of things?

Administrative Wife ....

E2. No...my illusion and I was...was to be an accountant. I did a 1'44...such, accountant. Because I went to the banks and at the last one, like that "hey fly, go away" I saw those grandfathers with glasses like that... [Gesticula] and sleeves...with some little books like that [gesticula].... For me... I used to watch them and... that made me become an accountant. When computers started to come out, one of the first computers in Valencia was mine. 2'06. It was from here to that wall, this big, the disks were like, I don't know ..... Besides, they were two or three bad years for me, because I had to work at a temperature of 19 to 22 ºC, otherwise, I didn't know what I was doing...of course in winter you could go in there, oh how nice, but in summer, of course, you weren't going to be putting on and taking off jackets... and between smoking then, and that, the costipao I couldn't get rid of it in three years, which is when I left 2'51. Well, when the computers came, I was already working in another place, they came for me, because the boss was there as an installer of electrical installations in construction sites, but not in an apartment, but in the whole property... Or, we were doing installations in the houses, in the streets, for the whole houses... and he wanted someone that as I and the little one were going together... so he said to me: "you come with me, so and so", so I became an accountant 3'43. But when the computers came I said I am no longer an accountant, "uy" no "and now what do we do?" .... Look where, there they were installers and then we had a repair shop for portable electrical machinery...drills, such and such and I also always liked to see the guts of the thing, I said send me your son and I'll teach him, and I went to the shop, that is, first I was an accountant and then a mechanic of portable electrical machinery and that's when I retired.4'19. Of course, one of them came along who was a bit of a nutcase, "Oh, I'm not an accountant, so and so", I told him, look, today he's an accountant, he's anyone, he knows how to use a computer, he's anyone, I call the guy I had and I tell him here, make this budget, start looking and I tell him what are you looking for, he says: "the little machine", I say no, by hand and he tells me "he wants me to go crazy, you're the only one who does that". Because I have always liked mathematics and I do the operations by hand and so on. I tell him see, he doesn't know, now you will see, I tell him to take the little machine, and after fifteen minutes or so, the budget was done, I say see, this is the computer, if you know how to use it you can also be an accountant.

Mar- The truth is that the ability that some people used to have to count, nowadays they don't have 5'26.

E2 Look, I am seeing that today there are very wise people, because I really like to watch a quiz show they do now at 3:30 "Saber y Ganar"...and they all fall into calculating and they are not big operations. You see that they answer things that you say but well, if they are not old enough to know it, but in calculus....because man, to a child who is ... How old are the little ones? five or six years old, no, they are already allowed to carry calculators to do the operations, I would first make them think ... and then, they should buy calculators that they know how to handle, because today if you do not know how to handle....

Mar-That is clear....sin computers and machines you are nobody, that is also true, but, that ability has been lost a lot 6'44 but well, each thing has its own thing. E2. What diseases do you have?

E2. Well, I don't have any illnesses, now, illnesses I've had...my life was, when I was nine years old, I started, I always notice it [she touches her throat], angina. I went to bed and when I woke up, oh, if I'm not in my bed and where I am, I had had an attack of meningitis, the doctor had already signed the death certificate...but, you see,.... Then, I had a myocardial infarction, I was going around the world with two million two hundred thousand red blood cells, then, lately, with one of my knees I wanted to leave for one of these sidereal ones...but it seems that I still have no place to stay, that they don't want me in any of them... [I sense that he is talking about death]. Because now I have an ICD, defibrillator, and since that day, now I have been operated on and everything and I am so calm, that is to say, diseases of saying no, no, but...

Mar-All right, do you have domestic help?

E2. Yes a couple of hours every day, after two years of fighting I have managed to get....

Wife. Financial help

E2. Yes, sometimes they give it to me at the beginning of the month, another time 8.47 in the middle...

Mar-What is the dependency law?

E2. Yes, after two years of filing papers, and some of them are even tripled or quadrupled...

Mar-Sure, and the resource they have provided you with is domestic help?

E2. No, no, they give me some money.

Mar Ah and you use it for... very well.

E2-So, like tomorrow, that I have to go out, she [the maid] stays with her [Wife] and she is not alone. Because, now, since she fell, lately she goes with the wheelchair, I have to lift her with a crane and lay her down because anyone [makes a gesture of being very heavy]... yes, she is there holding on, she stands up and so on to put the harness on her and that, but, walking...This summer I want to start again, because of course, between the winter and fifteen days or more that we were in the hospital, the leg loses a lot of muscle.

Mar-What happened to Esposa?

E2. Well, from a postoperative period, she was allergic to a product of the anesthesia, and so on... during the operation "hey, she is leaving, she has run out", they gave her adrenaline and as she didn't need it, when the anesthesia disappeared she got hit and they took her out, she had to be killed by necessity... and they don't want her anywhere [she refers to death]. She had a lump here [she points to the area] behind the nape of her neck and she had a few droplets....

Mar-A hemorrhage?

E2 Then also, that lump disappeared and the first time this happened, she recovered, she also had gallbladder surgery. But the second time, there was no recovery, since then. [Sad, tears in her eyes]....

Mar-How long has it been?

E2 Uff, thirty-something years, three or four years after we got married, I don't know if it's a golden or silver wedding anniversary, I've already lost..... Since 69 or 70... She used to go, but she had the bad luck to break her hip, but she kept walking, but the last time, she broke her femur and since then she has been in a chair. You see it and you can just see a little scratch [she points to the scar on her thigh]. Within this, we have come to talk to doctors....for whatever, with doctor (...), he is the surgeon, so: "hey, I will not operate on you, because maybe I will leave you worse and I am risking my career" 11'56 for that osteoporosis and with an x-ray and two or three (...) it left him with a leg that you can only see a little scratch but... the hip bone was operated on and since then he has been in a wheelchair.

Mar- Where are you from?

E2 From Teruel, well, from a small town in XXX. Tomorrow I'm going to the town. Ay [sighs long and deep] my father did very well... as he knew that we were both like this, he sold all the land except the house and I have kept my house, because that way, if we want to go one day, or so, you don't have to ask the family ..... As I go to my house, I go or not when I want to.

Mar- They maintain that independence, they go to their house.

E2 We have to do some things now, well we are going to do them because if not, a closed house always gets spoiled, you have to be there and now that you have it, now it is not for sale either. Tomorrow I go, we do some things, talk to the bricklayer.

Mar- Very well, are you both from XXX?

E2 She is from here in XXX, no, I said, with a coffee with milk and a Valenciana I have a breakfast [sardonic tone and laughter].

Mar- So, do you have experience caring for a dependent person?

E2 Yes, we have adapted

Mar- And how do you manage?

E2 -Well

Mar- How about me? 14'05

E2- Now, we also have that thing where they bring us food at home....

Esposa- Menjar a casa.

E2 Well, listen that we want to do....pues, because she has been in the kitchen for a long time.... and well?..... she has a carving board, I put some steel nails and she put a potato, a cucumber and not with the knife, also, they brought us a little gadget from....belga or I don't know where, that was unknown here and ras, ras, ras, behind her that nobody could beat her at peeling and.... she was conducting the orchestra and I was obeying.

Mar- Of course. Wife has been doing, let's say the house things....

E2- No, house stuff, no, because she couldn't walk, no. That was it, in the kitchen. It was that, in the kitchen. Because she would sit in her chair there and I was the cook, she would bring the casserole, the potato, she would peel or cut... sitting down. And then one day, I don't know how it happened, I went to the social security and I saw Menjar at home that so and so... I said: they won't give it to me because you see, they don't give us any help, I haven't been able to get one from the social security... because they come and they say: "well, you have so much... and she has so much".

Mar- Do you receive both pensions?

E2- Since she had not been working for two years when he gave her this and that, well...since she was not working and she receives the minimum wage, which also took us six or eight years to get it. Some people gave it to her two days later and gave it to us...when they saw her like that, they looked at it [the papers] and said "no, you earn more than the interprofessional wage and you are not entitled to benefits". I went in and said, "Is it paid?" ...... It's a pittance what it pays. By the way, it won't take long for [the person who delivers the food] to call because [she looks at the clock], oh no, it's 10:30 and she comes at 11:30. They bring us lunch, and dinner, well ..... Just the food, it's very varied and it's food for old people. There is no fat, little salt and ..... At least at noon we eat and we are both better off. Because as they are rations...you eat it and that's it. In the other way, you throw to stew and there is always something left over [refers to food they can't eat] and you eat it because you don't want to leave it or throw it away. Because with this [menjar a casa] there is no leftover. As to have (...) but neither and even if you do not want, but here as they are already fair rations, moreover, that we take a salad and today I have not looked at what we have, but let's say it says paella, because they bring some disposable tubs that you have to heat it and if you do not have microwave they bring it to you. But today, it is rare the house that does not have a microwave. With the same taper you heat it and with that and the fruit we already have enough. And the second course, one day it's fish, another day it's meat, another day it's a French omelette or Spanish omelette, another day it's sausage and so on.... different kinds of fish, meat, another day they bring stew, well with the stew soup, which the hospital director says "if it's noodles, add some chickpeas" because they found us a little weak, and so we do 19'19 we are a little low on dairy products, dairy products don't do much for me, cheese yes, but yogurts, milk and so on. XXX, the milk has to be skimmed and so on because if not, as she has a stomach operation, it doesn't curdle and she dances and it makes her feel bad... cheese she tolerates it. And now since we were in the hospital she says "no, all the flavored yogurts, leave them" look, natural yogurt but Nestlé yogurt, it seems to me that ..... seems to tolerate them. According to him [referring to the doctor] we are fine nutritionally.

Mar- Of course they are supposed to bring a balanced meal.

E2- And it says how many calories it has. XXX! please, [calls the assistant] can you bring me the food sheet? Well, today we have cream of spinach soup, it's good.

Mar. They are specified every day, it says everything they have to eat, calories, proteins, everything. I didn't know it.... well, I knew about the program menjar a casa, but....

Esposa- You didn't know about it?

Mar- No

E2- You can see the variety there is

Mar- Yes, yes

E2- Rice

Mar- E2, when you were working, Wife was ....?

E2- So she could handle herself, because since she fell and broke her femur.

Mar- I had understood that it was from before

E2 Then a lady also came every day and helped her make the food and so on, but, as there were times when I was a little late and arrived just in time, she got nervous. At the end of the day, I would stay for lunch where I worked. The lady did the cleaning, lunch and some dinner. She was still walking, going out and .... was when she broke her femur, I said to the boss: "hey look, see if you can throw me out on the street, because you pay me a woman? I'm going to work, but I'm not going to work and be left without money and without..... You pay me a woman and I come to work, because now I have to stay at home". He took a pre-retirement and since then ....ella walked, but since the femur, not with the chair. Well, she changed from the chair to the other chair and so on, we continued cooking, but now, well, we also have the bathroom adapted, and they also gave money to adapt it and ..... what a coincidence, when I went, they didn't... Besides, they had to come and look at the kitchen and the bathroom and since it was already adapted I thought they wouldn't give me anything. We removed the sink that was in the bathroom, we put a plate, she holds on to the railing and with a garden chair, I put her, we bathe her or the lady bathes her, I dry her and 24'55

Mar- So you do everything, except during the time that the lady who helps them is there?

E2- When she doesn't have a lot of work to do, she says: "well, I'll bathe her"; that's fine.

Mar- Well, but this is now; until now it has been the other way.

E2- Well, but at the end of the day she is also the one who receives the dependency law.

Mar- How long have you had this lady?

E2- Six years...only. The previous wine....yo who was....

Mar- But 2 hours, the truth is that.... Well, I was thinking that you are really the caregiver.

E2- Yes, now, for example, they bring us food and when it is a nice day we say: "ok, let's go downstairs for a while or, look, we have to go to buy this or that and with the "convertible"...

Mar- Sure, why do they go out?

E2 -Not as much as we should but, yes, around here [in the neighborhood] or at the most we go to the garden that we call "aviation", the XXX garden...

Mar- Yes

E2- Since we have two gardens around here, what we are interested in is that the sun shines on us and when we finish [the interview] and they bring the food, we go down. If we have to buy something, we go down before, if we have to buy something from the fridge, we go to places that are closer.

Mar- Of course, it is organized.

E2- Or to the doctor, who is now closer to us.

Mar- Yes, now it is in XXX and before in XXX.

E2- We are halfway there.

Mar- And it shows.

E2- Not to the one who walks well, because hey, not for you, but, for me it's a walk to go downtown.

Mar- Of course, of course it shows.

E2- That it costs us a little more, well, that's good. Besides, I go more calmly when we go "the whole house", we go at the pace we feel like. The other way [only] I think "she will want something or not and so on", I don't have to run, but I go at a faster pace. Let's both go,...let the time go by, let it go by, nobody calls us, nobody waits for us, otherwise, I go more nervous and more ......

Mar- Have you noticed that taking care of yourself has affected your health, your relationships?

E2- No, well...in relationships, it depends on how you look at it, because now, I know I'm going too far, but it's like that "spurring her on", because before, she was more lively and stuff... and now I don't know, it seems that she has become a "Carthusian nun" [looks at Wife], right? Right. It's not that of "XXXXX", but I would like that if it had happened to me, they would also do it to me, you know. But she won't say that I have never complained about anything, no.

Mar- They have managed well, they have complemented each other.

E2- I have a way of thinking that if I don't want something for me, then I don't want it for others either. It's the same as... since she has been known so much by her friends, that I have never set an appointment time or day, I have always wanted others to set it, if I have arrived at the appointment half an hour earlier it has been because I wanted to. Now, a quarter of an hour after the time, don't look for me because they couldn't find me. Well, I think that what I don't want to have done to me, well, why should I do it to the others.

Mar- Yes

E2 -Yes, there are those who do it better. .... Because when she got this 30'26 in XXX, someone we knew also got, well, .... that one, a year later, or a little more than a year, "there you are, goodbye, I'm leaving".

Mar- There are people for everything

E2 Now is when we go out less because, before we had or we have the car, which is rotting and we used to go to the beach, every Saturday, Sunday and holidays. Because we knew we could park there and since we always went to the same place, we were already part of the family, and you had someone to talk to, because you already knew everyone and so on. They went for a walk, we would leave the car and XXX would say: "I want to walk", he would get up from his chair and I would sit down. Those who knew us, said: "eh animal, poor XXX walked and you in the chair, eh," and those who didn't, "poor things, look, she is walking and he is in the chair"....

Laughter from both (E2 and Wife)....

A- You know.

Mar- Which beach were they going to?

E2 '-To XXX, next to XXX.

Wife- To XXX

Mar- Where were the little pink houses?

E2- There is nothing left.

Mar- Nothing, that is now unrecognizable.

E2 -There were times when I had to remove the car because the water reached the wheels of the car.

Mar-Yes sir

E2 and ...Since we already knew each other: "Hey, how was your week? How about this and that.

Mar- Now do you have any friendships?

E2 -Well, friends we have

Mar- Do you go out with them?

E2 No and even though we're fine. We have been of a way of being ... Because friends, we didn't want apartments or that, because they were all year tightening their belts and then.... If you do not go to the "bacchanals" "goes these miserable" "goes these that I do not know what...that I do not know how many". Yes friends all, but each one in his own house....

Mar- Friendships, that's what I say

E2- Here everyone had villas and so on. Now, I say "what do I need" and immediately everyone. They are all widows now. When they were younger, when (Wife) fell and broke her hip, by the time I arrived, when I was at work, they had already lifted her up and....

Mar- She had help.

E2- Yes, well I was then, I arrived and "if you want something, you know where we are" right away everyone and then everyone went home. It was like the town, you say "eh" and then everybody was there and then everybody went home.

Mar- Yes, a family relationship.

E2- Yes, but let's say that you don't have friendships like that and then you get into fights. That way you always have friends. I remember Cantinflas, when the English with their "phlegm" asked him "Hey, have you ever been proposed to be president of Mexico?", "Well, yes, more than twice" he says "but I have many friends and if I am president I will stay with them". We (XXX and he) preferred to have many friends, let's call them friends, but without reaching that friendship that later, we know that they start quarreling.

Mar- He told me that they had put a crane on him?

E2- No, they don't put a crane on it.

Wife- We bought it 35'36

E2-They don't give it, it's the same as the chairs, they give it.

Mar- But you pay a percentage

E2- No, they only give it when they give you air to breathe, they give you an allowance for the device. But like the crane if it is electric, but you charge them once every three or four times.

Mar- I thought that the crane was the same as the chairs, the crutches and everything....

E2- Now also, you pay for the crutches, since we are in a recession.

Mar- Yes, yes

E2- I think they still pay for the chairs, but not the crane. That is the same thing I ask XXX for, I tell him boots, they pay me for the iron if he puts shoes, but not the shoe. What he puts on boots is orthopedic, even if it is an orthopedic shoe.

Mar- Yes, of course, and what is your relationship with the family?

E2-Well, with the family. As she (XXX) has a sister and she is not very "buoyant" either, well, we have her right here but we can't get help. According to her, all the doctors find her very well (she has had a breast operation for cancer) and she says she is going to be the healthiest one in the cemetery.

Wife laughs

A- She can't eat dairy products, they give her I don't know what, she can't go downstairs to the garden, she has

Wife- Allergies

Mar- Sure, in the spring.

E2- You can't count on her either. One day, she was waiting for the bus, there was a gust of wind, a piece of paper fell on the sidewalk, she was going to put her foot down and she fell into a small sinkhole, she twisted her leg and her knee went to pieces.

Mar- Oh my God.

E2- So, yes, good relationship, but you can't count on anyone.

Mar- How do you manage?

E2 - In the mornings when I get up I say to myself "a la robotito, you've arrived". Because my knees are not mine either.

Mar- Are you operated?

E2- Yes, as my feet had also had some of this, although they wanted to leave my leg straight, well, my feet don't feel good either. The knees, sitting down they hurt me a lot and I have two prostheses and this XXX says "no". In the hospital that knows me they say "hey, they look really bad" and they all prefer that XXX (he is very good) do it to you, so much so that I have not even gone to rehab. The guy from RHB comes, looks at me and says "oops" I say "what's wrong?" he says "I've made the trip for nothing" why? he says "let's see, stretch your leg" I say "like this? "And you want to come to RHB, if we are not going to do anything to you" I said "man, I can't even come to RHB" he said "well, if you want to lose a month of coming every morning, then lose it". When the second one (knee), I had to go there and she also looks at me and says "I would say" I say "yes, we know each other, right" she looks at me and says "I think I made you come for nothing, well, let's see" the same thing, she says "why" and when I leave as she went at least three or four months to RHB when the femur, they knew her and I meet the head of the gym and he says "what are you doing here?" I tell him and he says "no, why do I want to come". But there was a big, strong guy who was always messing with XXX "hey, what are you doing here" I said "well, I came" 41'16 I said "hey" (because they went up to the room until they put the cast on me to make me RHB) "hey, I have to tell you something, when you give reports of a patient, he looks at me and saw that the head of the gym was laughing his head off and said "I'll give you a kick" and I told him "no, you don't need to come to RHB". 41'53

Mar- Did you do the RHB?

E2 -No, I would come home and, maybe, I would be sitting and I would be like this [does the exercises] putting in, taking out, stretching, but hey, he says to me. "if you're going to come here to waste time, if you go to RHB, you more than bend it". XXX was also going to a gym, of course. Then the girl who came, who was there for a long time, took her and picked her up.... near where they have now put the new ambulatory, where there are some gardens, a farmhouse... and there were... Well, some people went there and as they saw me bent, [the knee] they said: "the husband of this lady (all operated in private clinics). One day, I don't know how it happened, the girl couldn't go and I said "I'll go", I went and said "today I'm the one who comes for her", she said "man, how are you doing?", "How am I doing, you see, look" and the other one couldn't even put his knee like that [gesticulates] and when he went, by the way, he had a store 43¨32 in the tunnel in XXX street, what is the name of the other street where the passageway was...?

Wife- XXX

E2- XXX? it was. He had been operated on in a private clinic by a good friend and so on and he said [to his doctor], "hey, what have you done to me, you, who have been operated on in the hospital, three days later they have thrown him out on the street and he bends his leg as he wants and as he feels like it", "no, it's just that look, it seems that the prosthesis is a little bigger than what you had" Excuses. But, he couldn't bend, he couldn't bend his knee like that [bends his knee].

Mar- Also, it makes a lot to be a vitalist

E2- I don't know what it is to take a good step in this life.

Mar- I don't know, but you.

E2- Because, I was born with equino baros feet, at forty days I made my first trip from XXX to XXX, to the "XXX", which is a saga... brothers, uncles, cousins. Here in the hospital there are one, two. One sister had an orthopedic shop, the others, there on XXX street, were with two or three others, for the feet. Complaining? Or not complaining, I could see and cast them, because I know what it is like to walk with a cast over the snow, you know, I still have bruises from that, look [she shows a scar on her leg], this is from a grain of wheat that got between the cast and ...... You know?

Mar- Mother, you will agree with me that you are a strong and vital person.

E2- I have no choice, when they operated on both my feet, the surgeon at the bullring, now I don't remember his name....

Wife- XXX

E2- That XXX, he wasn't XXX's doctor, he was a surgeon, he was always in the bullring 46'31. Because according to that, he said "no, no, I'll help you and let's do it because if they take him away I have to uncover him right away and come back in case...". He put my feet, I was almost walking on my ankle. Since then I have been walking on the sole of my foot. Well, it was a pure effort and they sent me to a gymnasium. Then, the kids would stare at me and those who worked there would tell them "you see, if you do what I tell you to do, then when you grow up you will see what kind of chest you will have and so on". The kids would kill themselves to do what he told them to do. ....

Mar- You can tell he was a very strong person.

A.- There was nothing else. Doing what we could.

Mar- It is not easy.

E2 -My mother died, I never loved her, because she was one of those women who liked to be under the skirts. I don't know if she felt guilty that I was born like that, or if she was ..... And then always under the skirts. My father, my father, no, my father was one of those who said "look, you have to do this and you'll manage", of course....

Mar- Yes, it was to motivate him so that if he had a problem he could overcome it, is that what you mean?

E2 -In the village, he would go to work and back then you still had to do the "harvest camps". My great-great-grandfather was dedicated to buying land everywhere until one of his sons died and since then the daughters shared it out.

ASSISTANT - Goodbye, have a good time.

Mar- Goodbye

Wife: Goodbye.

AIDE - I'll come earlier tomorrow.

E2 -Yes, because I have to go into town.... Y....mira, "I'll leave you the horse and tomorrow you'll go to town and do the camp for four kilometers around". He (his father) said, "hey this is not right, tomorrow you come back", and the embarrassment I was going through...was more than if my father had.... me. So. My mother would have come with me, she would have done such....

Mar: Or would she have done it?

E2 -Here, we came to XXX 49'29. She "there's we will have to..." My father "let her look for the academy she wants, then she can't tell me that it's my fault she didn't study, not that...it's your fault" that is, between how independent I have been and my father's fault too....

Mar- Has encouraged in you....

E2 -Well, my mother says that I didn't love her, because of course, "where have you been, what have you been doing" huy, if I went to the park I would tell her that I had gone to XXX....

Mar- Do you have siblings?

E2-No

Mar- Only child

E2 -The one with the woe, which is worse than having siblings. Man, while I was in town I didn't need siblings, because to cross the street I had the family and we have always been together. A moment ago we spoke with the cousins who are still in the village and I told them "but you can do it yourselves, rent" and they said "no, you come, it's better" They will guide me but... and they know that what they do is enough, but, you can see that they have had a stumble with some family member.

Mar- It is good that you are there and you know about things. 51¨55

E2- No, I don't miss the brothers. Then I came here, as well, I don't know how it was but I became part of the XXX, all of them from Maños and a "peara" of friends "hey look, we are going to such and such a place, but we are waiting for you". The July fair arrived, the XXX put up a pavilion "hey you have to go out", "but, if I don't dance", "then you take the fresh air". With them, at the fair, I didn't dance and I stayed at a table, but, the parties we had after and before. So, because of my character or whatever, I haven't found myself alone either.

Mar- How do you see the future E2?

E2- Well, why not? I think that Spaniards are like this... "we have and we eat, we have less, we eat less and we don't have and we look for ourselves and we adapt ourselves, even if it's to herbs, I think". Maybe, now with the youth, as they have found everything already more done. Because I tell them "if we went back to those times, you would go hungrier than me, because I would go to the countryside and as I know all the edible herbs and all those of that.... I would at least eat vegetables".

Mar- It's a different way of living now, we live differently.

E2- I don't want my children to go through what I have gone through, they have been raised without being taught what it costs each one of them....

Mar- And that everything has been easier, E2

E2- Even if it is easier. Because you see, we here [in XXX], we were the parents of a cuadrilla. They were all university students, both girls and boys, and those also "hey, come on, come on, come with us here or there", but boys if we are.... Their parents did not go. There were also those who struggled to earn a "pela", there were some who did. There was one who had two careers; he had been a translator in XXX, he was a waiter in the bar where we used to go; others were maybe substituting as doormen, others as postmen....

Mar- To make money.

E2 -Girls that .... There was one who passed by every day where I worked and she looked at me and said "if you tell me mom, I'll kill you", she went with a child to walk him and maybe I told her: "bye mom", that is, we have dealt with people who could be our children, who have had us as one of them. Commenting to you, "that they have slept together" well, you are older and we have never said "hey, that is not right, that so and so" hey, you will manage yourselves. You are older, and with more education than us, you already know what you have to do. No reprimanding them, no....

Wife- Preventing them from doing it.

E2- So, they were friends, and I tell you, we could be their parents. I said, "hey, why don't you come tonight to the Malvarrosa to see this or that" but, you know how many people .... "ah but you are not in a hurry". Well, they were already waiting for you somewhere, all of them, boys and girls "come on, let's go, we can go", maybe it was to see a castle, as they all knew each other, because they were from XXX, so "from here we will see it perfectly and this way we are not in the fuss" 56'21. So we were not alone either.

Mar- And do you feel strong enough to continue taking care of them?

E2- As long as I can, yes, because right now, it would have been different if we had had to lift her by weight [he refers to the care of his wife, especially to the mobilization of his wife, who is obese], then you need a lifter from ..... She would have suffered more, because to lift her you would have had to hold her and even if you don't want to squeeze. As now she puts on the harness.....

Mar- Sure, with the crane?

E2 -You press the little button and [simulates the noise of the crane working], it's a huge facility, don't you know the crane?

Mar- No

E2- Come in and I'll show it to you.

Mar- We leave her alone for a while [We go to Wife's bedroom and there she explains to me how the crane works. The window is open and it is justified... by the smell that Wife makes].

E2- It is placed over here (the fastening straps), she doesn't have the trouble of hugging her, everything more....

Mar- It's an advance.

E2 -I put her pants, shoes and top on, take her out, let her sit down, put that on [points to the bindings]. Before there were other [cranes] that you had to anchor them to the wall. This one has ....

Mar- I find it comfortable

E2- And with a harness, well, that and with the straps that you put on the back. Then you pass them under here, of course, you cross it because that way the legs. Because if you don't, even if you don't want to, the legs open and you can... that part [tape] you bring it here and it goes up as if it were a capacito ale because of the heights. 58'59. She can go to the toilet, I take her with the chair, she holds on to the sink, she stands up and little by little... now, when we did the toilet, we raised it at least 12 centimeters, now with the bar, she puts her hand and helps her to get up. Since he has been in the hospital with a urine infection caused by a small stone that he had in his duct, which did not allow him to urinate, it seems that it was very difficult for him to urinate. I went and bought a supplement [for the toilet] and she is almost sitting down. I put my arm under her armpit and without any effort, she stands up, I wipe her, because before she could, but now, with the insert she can't put her hand in and she can't wipe herself. I pull up her pants, pull up her panties, pants and so on, she sits on the chair and then, come on, let's go....We have to conform, we are of the "look, there is a wall, we have to cross. Take a run and boom, bye-bye wall". Otherwise, we wouldn't leave the house. It's my life.... And now this.... No. Now is when we go out the least, then, the weather is so crazy.

Mar- Of course, the time

E2- Saturday all day, well, and then, at night, what a downpour.

Mar- The temperatures dropped a lot.

E2- According to yesterday [weather news] here 6ºC and in other parts of the CV, up to 10ºC.

Mar- Yes, it was noticeable.

E2- On Sunday, we were 6ºC lower than on Saturday.

SILENCE

Mar- Do you want to tell me anything else?

E2 No

Mar- You seem to me to be a very strong person. Will you also have your down moments?

E2- Since XXX had this disease that I call fashionable.

Wife- Depression.

A- Eh, the doctors told him "either he would get rid of it or he would manage", because if they gave him ...

Wife- Antidepressants.

E2- They would be detrimental to his illness, and if not... he would be fine. Well, if I find myself like this .... a little bit .... ... and what do I do? Who do I cry to?

Mar- And what do you do in those moments?

E2-Well, who do I cry to? Well, listen, I put myself there [points to a work table] and I entertain myself a lot, because I'm not Valencian, but I'm almost more than others...I have a lot of books in XXX and, fortunately or unfortunately, as I like, well, we both like, everything that has "manchitas de mosca" [books], well...When they put XXX and started to do theater, sainetes, Escalante's so and so. I was recording it. Then I saw that, in the press, they came out and I started buying them all. I thought "what a pity, my cousins from the village can't read this" and I amused myself translating. The translation may not be very well done, but....

Mar- You enjoy it.

E2 -I have a great time. 1'03

Mar- You're translating, it's fun

E2 -I have a great time. There is still that thing like in the villages "hey, you don't know who I am", people who wanted to live beyond their means and lived that they owed more than... and of course, on the stairs "hey, you don't know who I am" and maybe they started "look, my uncle's cousin's niece, she had an uncle who was ....". And what do I care if my father is called "coke" if I'm starving. And of course, I see that as so appropriate...Like now "La alquería blanca", I see it as the same as what I have known in the village, in XXX. There have been times when I have arrived in the village and I could hardly speak Spanish, because of course, it was winter, I had to come to the doctors... there I had an aunt, my mother's sister. Snowing and all that, well, I stayed here. Then, when the summer came, when it was harvest time and so on, and I had to come to the doctors, I stayed here. And I found it so, well, everyone was acquaintances and family. The villages were smaller, because maybe my uncle would say "ale let's go to..." from the age of 7 until he retired, he worked in the same place, in a factory that made pots and pans, clay pots. He worked in the factory and at the same time he worked cultivating the fields of the masters. He was also one of those who was told "ye XXX see if you can come with the horse" he answered "talk to the master" 1'05. He did what he wanted. I see him as the people were. There was the custom, which was, at that time, to put a small table on the sidewalk of the street and when you passed "bona nit, sopant, si, vols ...no que aprofite" to the other "che que soparot, vols ....no, que aprofite".

LAUGHTER

E2 -Then, in Canal9, it is more, mixed, because those sainetes that are from 1860-1870, then, there was not yet the "catalanisme".... That first it was the Catalan and then the Valencian and now this translator, whenever he went there, he always said: "ye XXX, parlem el valençià que tu parles un valencià bonico, no como yo que ya no sé lo que parle" there he gets in every .....

Esposa- We speak Valencian from the Vall d'Albaida region.

Mar- I don't speak Valencian, my mother was Basque, my father was from here, but, at home we didn't speak Valencian...

E2- In my house neither, but, as I have been a long time in XXX and now when I go with my cousin I tell her "as you speak to me in Spanish...

Wife- My grandparents didn't know how to speak anything but Valencian, and my mother didn't know much more.

E2 -Ah, I enjoyed it when there were the two XXX ace, who spoke Valencian, I did, I spoke Spanish, but I enjoyed it and now when I go with my cousin, who is from XXX, I tell her "if you talk to me in Spanish, the little nose you have left, I'll give you a flat nose, eh".

LAUGHTER

E2 -I like it and it is something that should not be lost and at home, my parents, Castilian, and in the village Castilian. But, I.... While she watches the novels, I come here for a while....

Mar- And she does her things, she has her hobbies, her entertainment and her activities.

E2 -And if not, crossword puzzles, because I'm afraid that this [she points to her head] won't "carbure". Yes, it can happen to me, because I used to say that nuns were the ones who had more Alzheimer's, because, as no....but now, people with a lot of studies also get Alzheimer's, so it's not only because they don't make the brain work.

Mar- It is an epidemic

E2 -I don't know if it comes later if you make it work or not, but I try to be active. Reading, if you read aloud you get tired .... and if you don't, I don't think the brain is exercised ......, so I try, one line, again, a little more than one line, of course, if there are rare words, then even that rare word. Then, I take my dictionary and see. Because, there are some who write, for example, Escalante, the "ñ" does not exist in Valencian, however, he wrote the word with "ñ", because he wrote the same as he spoke and so anyone could read it. What he did write as it was supposed to be written was "roig" instead of "roch" and such....., if they did not have many "lights" they would say "aixo que voldra dir"..... He wrote badly because that is not admitted...... no? But, he wrote for the people....

Mar- That way people understood him when they read him.

E2- It's not like now, that more than less they have more....although now with this amount of new words they have brought out, you have to carry a dictionary in your pocket.

Mar-Everything is changing.

E2 -But back then, the one who could read was already ...

SILENCE

Mar-All right E2.

SILENCE

E2-What I can't, is, between how bad TV is and that, to be like this [crosses her arms].

SILENCE

Mar- Do you need help? Or have you needed it and not had it in relation to care?

E2- Man, no se....no, because.

Mar- Have more resources

E2- Well, we have "aviao" as we could, but without the crane, I would not have been able to. One, for not being able to make efforts and the other for her [Wife]'s sake. Hoping that she could manage better and so on....First I had a rented one (crane), then I thought, if this is going to continue, well, I prefer to have it, to buy it, yes, I will spend some money, but I prefer it. For example, to go to the orthopedics where I always go and pay for it in installments, but it is already mine and not, for example, to have to pay ........, I think it was five euros a day, a lot of money .....

Mar- Is your pension less or more than 1000 euros?

E2 -Let's say in the 1'13 range.

Mar- Man, it is a very important factor

E2 -I had the bad luck that I was two years away from retirement, when XXX happened, then, unemployment for two years, but then there were eight months until retirement. If I kept on paying so that I could have a pension as it should be, I had to spend I don't know how many years to retire. In the other way, they took eight points from me, so hey, eight points, well ...., we have gone ...., as we do not have big vices or big talents ...., well, we have been adapting. The first thing has been what we need to live, because you see...the house is not...., but we have adapted it to our needs.

Mar- Adapted to their needs.

E2- And there is another room that until recently was a gym. On the door there's a carriage, on the foot a weight ...., there's also a bar, which is where she started to walk. As her leg was moving towards this other foot, I put two strips of adhesive tape so that with skill we could solve it......1'15.

Mar- What has it meant to you to take care of your wife?

E2- Well, a duty.

SILENCE

E2 -I have already told you before, if I would have liked you to do it for me too. I don't have to be "sorry".

Mar- If I ask you, why do you take care of her? Why do you take care of your wife?

E2- Because, if I didn't, I wouldn't have anyone to fight with.

LAUGHTER

Mar- Well, it's an answer.

E2- To complain... everything. Look, we have played with people who said "there's honey, there's love, whatever you say honey, whatever you want my love" but, you could see that, with one hand like that and in the other was the dagger in the back. I don't know how to say nice things, I prefer to do actions than to be told those things; a word comes out... and goes away, action does not.

SILENCE

Mar- Do you love your wife very much?

E2- I don't know, whether I love her or not.

Mar- Do I ask her?

E2- Since I start because I would like to have it done to me, well, that's why I do it.

Mar- But, look, if something happens to you in the future, Wife won't be able to help you.

E2- Too much grief.

Mar- So maybe what you are doing is based on love. 1'17

E2 -Because right now, when I had my stomach operation and so on, I had a woman here 24 hours a day.

Mar- Yes but now, you don't have a lady 24 hours, you do everything.

E2 -But now that I have to have a hernia operation, I have already told her "hey, you two or three days" according to Dr. Puche, that's it, operate me, spend the night as the one who says, if there are no complications and the next day home "can you stay with XXX?" Because now you look for someone and until she settles in and gets with her.

Mar- It's a problem

E2- It's a problem, then I prefer to pay her a little more, if she can stay, eh, because she already knows how to treat her, as such....

Mar- Then let them take care of you

E2 -Me?

Mar- Man

E2- Yes

Mar- Man, from the hospital you will get out and you will have to rest.

LAUGHTER

Mar- He won't be able to be up and down.

E2 -No, no

Mar- He will also have to learn to be still.

E2 -No

Mar- No?

E2 -Because like that one...I went out, I didn't make any effort.....

LAUGHTER

E2- No, when the stomach thing, at the time... "I'm going shopping". I had a slight heart attack, the doctor said "well, look, if you promise me that you are not going to get nervous or anything that might upset you, go to work" because here at home, we painted all these pictures together to keep me entertained, but now... staying at home, I think it's counterproductive... go to work or do whatever you want, because staying at home is counterproductive 1'19. So she could walk, she could... couldn't she? So much so that one day I was in the car and someone hit me from behind and... as soon as I got out I heard him start: "calm down, calm down, don't worry, nothing has happened here, calm down", I thought, why is this guy telling me so much, calm down, calm down, calm down, calm down, calm down, calm down, calm down, calm down, calm down, calm down, calm down, calm down, calm down, calm down, calm down". We looked at each other and I said: "Oh, he was one of the workers who had been with me in the hospital, they filled you with daisies and we had a TV".

Mar- The daisies are the electrodes?

E2- Those electrodes and when .....

LAUGHTER

E2 -I moved a little bit or pressed them, it started "pi, pi, pi".

Mar- To beep

E2- What's wrong? What's wrong? Nothing, I thought you were sleeping, I told him... He was more scared than I was. He thought "now he's going to have another heart attack".

LAUGHTER

Mar- Do you have a date for the hernia operation?

E2- No, not yet, no because I have to go .... this week, to the anesthesiologist.

Mar- Sign the consent form

E2- No, no way, that.... 1'20. To the surgeon and that they give us a date for the operation. I'm doing great, as long as it doesn't come out, I don't have any pain. I told him about it because....

Mar- He would be worried.

E2 -When he saw her he said: "we have to operate" Again, and if I do not operate, "of course you will operate, you want to come to the emergency room with a strangulated hernia and what you are going to suffer". Immediately the papers and .... like that one, even if you don't want to be 1'22. She leaves the hairdresser's and everything... she does not leave me alone. When it came to the operation, she swallowed it all.

Mar- This will also give her some peace of mind, to know that there is someone she can count on.

E2- Right now, I tell her "let's see what day..." and she says "well, Tuesday, since I'm not working". I tell him Mother Teresa of Calcutta, it is like that with everyone. At the hospital when we go to .....

Mar- They know her...

E2 -She is like one of the cousins in town. He is also a Samaritan

They ring the bell and bring the food, they have the resource of "menjar a casa" 1'25

A- Look at the food

Mar- When do they eat?

Wife- At two o'clock

**E3**

There is an initial conversation about telecare and the treatment you receive from its operators.

E3- They (telecare operators), they tell me: "you call us so that the batteries don't run out", so and so....

M- Of course, you have to take those things into account.

E3-They are very attentive in everything. Well then, let's see what you ask me that is not too big.

M-I just have to ask you, what is your caregiving experience? What do you do every day? Is it your husband that you take care of?

E3- Yes, he is my husband

M-What is your husband's name?

E3- My experience so far, woman, I get angry from time to time, to see that he doesn't do. I know it's wrong, but, that he makes so little effort, sometimes I yell at him eh..., my daughter also, she says "Dad, that can't be" .... now, my daughter doesn't work. She has been a lot in XXX, because she is... I don't remember now what career she has, she is from XXX. But she didn't want school, what she wanted was an office. And she has been in XXX a lot. Now she's in XXXX, sometimes in XXXXX and sometimes in whatever, they call her from time to time or send her home.

M- What does she have, 3 daughters? 2'28

E3- 3 daughters, another one is XXXX and the other one didn't want to study, she did ...., she is in an office.

M-All right

E3 -Eh, I don't know what they do now, she only did two years....

M-Very good

E3 -And this one, she never wanted to go abroad and of course if it was XXXX or XXX she didn't want to, it's not that she had children very early, but. Then, when she had children she never wanted to leave here. So her position is..., wherever she went she did not approve, I mean, she could be in the Generalitat or..., but she did not want to, she did not accept it. And my husband, well, sometimes I get angry, it makes me feel bad.

M-What happened to XXX?

E3- A stroke, well that's what they say, a cerebral infarction. At first it wasn't very big, he still had a little, a little bit, but after repeating it, well, less. Also RHB, patatán, patatín..., but I tell you. I think that... and we all think it, it's not just me, my daughters and my sons-in-law and we all think that he hasn't made any effort and of course, he goes in a wheelchair, no, we have to carry him, we have to sit him down, and so on and so forth.

M-Every day What do you do? 4'23

E3 -I have to help him get up, I bring him breakfast, I bring it to his room, he takes it and I put him back to bed.

M-He can he take it?

E3 -Yes, that's right, if I gave it to him it would be better, but okay. Now, now, I can't take it anymore and now, since this is happening to me, it's a little more uncomfortable. Since my daughter doesn't work, she comes in the morning, but I manage everything else.

M- Your daughter is coming now, why did this fall happen to her?

E3- No...., well, she comes since she doesn't work, she told me "Mom, I'll come in the morning and I'll help you to lift it", we left it on the chair. Now you'll see if you want to ...., and I'll manage. When I take him out, he grabs it from here (points to the table), he holds it, and I change his diaper and back to the chair, here I bring him his food and so on until dinner time, but, when this happens to me (a fall), a girl also comes on weekends; my daughter has already told me "mom, I can't come on Friday" well, when you can't, don't come, what am I going to say (hurt, resentful), if you come it's great. If I have to thank someone else, I try to thank them and that's how we get by. I don't know what else I can tell you?

M-How do you feel? 6'03

E3- Well, there are times, now for example, this morning I was crying (cries), because it is very painful, even if you do your own thing, you have to do it, but of course, I have the other thing (looks to where her husband is). My daughter did not come as soon as she wanted. She has to take the kids to school now that she doesn't work, otherwise she has to make do with her in-laws, who are saints. I tell her that she has neither words nor deeds to thank them for. It's just that I'm a bit far away. She lives in Sagunto Street, the others live in XXX. If I call them, they come right away, but if I don't call them, well, then, well, .....

M-Well, they are not coming.

E3- Well, they don't come (desolate, sad).

M-They don't come and you have to do it?

E3- I feel bad crying...

M- You don't have to worry, XXX

E3 -Does XXX know about this?

M-These things...., XXX, this is not going to listen to it, this is for me.

E3- I don't care, eh

M-Besides XXX, I want her to understand that I understand her, because she lives a very hard situation.

E3- But since I'm defending myself for the moment, that's it. Of course, you can't pay for everything you want or need, and of course..., and I have no complaints from anyone, neither from doctors, nor from anything else. When I say something, they immediately come to me and everything, no, I have no complaints about anything. But of course, these days I don't dare to go out, because of fear, because of this (she points to her arm) and because of that (she looks at the room where her husband is), well..., I feel a little bit more suffocated, but well (she gets over it), between one and the other we are solving the problem.

M- Sure, but it is very hard for you to have to be in this situation every day 8'21

E3- Yes, but

M- Why do you think that XXX does not react?

E3- I, now I think it's almost normal, he should have reacted before, eh. After we were going many days to the hospital for RHB, but, "you have to do this every day at home" they told him. He did nothing.

M-He nothing

E3- I bought him one of those devices (he makes movements with his legs and arms), he gets tired and says no "Ja estem desocupats"...., Anyway we are solving it and then, what else can I say?........................SILENCE

M-And he is getting worse and worse

E3 -Neither better nor worse eh. Because, however, sometimes my daughters celebrate their birthdays and they want us to go. Once when I didn't want to go, especially this one (the one who helps in the morning), who is a bit more than ....., she got mad at me "and why don't you come, tata, tata, tata", "but woman, don't you see that we are not ready to leave the house". I, even I can go out and before I could go out better "as the father is, I think we are better at home than outside".

M-But, why do you say that you can't go out? Because it costs you a lot to prepare it

E3- Woman, it's a little hard for me, because if he goes in his pajamas, or if he has peed, normally you have to change him, but if you have to leave the house, it's always a bit of a challenge ...., I, this Easter, this had never happened before, I wanted to go to the village. We have a house in my parents' town and I really like to go to the village. We used to go there a lot, when this had not happened, but now, we don't go almost at all.

M-Where are you from? 10'16

E3- From XXX, from XXX, well, close to XXX, it's very close. I always say XXX, which seems to be better known than XXX, although the town has become quite big and all that..., and I like to go there a lot. Why? ..... (Sobbing)

M- Do you have friends there?

E3 -Many, many, and a brother who is a sunshine (cries) and now, being.... I feel bad crying.

M- He doesn't have to feel bad.

E3- And now, he would come and help me lift him up and kick and kick and.... "you do what you want". This (the arm) happened to me the first day I went and of course, I was better, now I'm a little sicker, since I came (from town), and of course, this takes a lot of courage away from me because I thought that if I didn't have anything, unless I had something else from the blow, because of course, it almost always happens.....

M- Are you afraid of having something else in your arm?

E3- Woman, what do I know, if this pain doesn't go away, that it will pass here or that my ass hurts. LAUGHTER

M-But, the punch you took, how long ago was it?

E3-It's been almost a month, it was Holy Tuesday, I almost don't remember.

M-Yes, a month ago

E3- That's right, a month and some time ago.

M-You would like to be better, do you think that...?

E3-I think I won't recover, that's what I think, that I won't recover. Three or four years ago, I also fell in the market, I broke my shoulder, it seems that I did so much what I did, .....

(He looks for a handkerchief and cannot find it), the doctor said: "you have a broken shoulder, if you do this, let's see if it is not necessary to operate". (I give him a handkerchief) Thanks.

M- We have one handkerchief left

E3- Yes, you can see with a handkerchief LAUGHTER. I feel bad

M- No, no, no, no

E3- Anyway, I ....

M-The arm did recover.

E3- Yes, they didn't operate on me or anything. I did do what I was told (looking towards her husband's room). When I had RHB, everything. I said "don't hurt me so much, even if I have to come more" because of course, they make you a little bit of ..... The specialist told me that I had to fasten my bra and fasten my apron in the back. And I said "I don't wear an apron and I can fasten my bra in the front" LAUGHTER. He would say, "no, you have to fasten it in the back, "total....".

M- Of course, they told him so that you would do that exercise.

E3- Yeah, yeah, that's probably what it was. I mean, I'm different (looks towards XXX's room), I'm able to, hey, they tell me gymnastics...... I, I don't go to the gym around here because apart from the weird things they do, I don't want to do them and they tell me sometimes.... because I don't walk very fast, because, well, before I was lighter, now less and that's what I tell myself "I'm in a hurry" LAUGHS

E3- In an excursion we went on, even when we were young, when we arrived at the hotel, there were other ladies who were from XXX and they were very funny, they said: "Oh, we are incarcophas" and that made me laugh a lot.

M- What has your life with XXX been like? 14'42

E3- Woman, there is everything, in the family, there is everything. As far as I can tell, it's been pretty good. Well, I think there are always things in marriages. There are almost more now than there have been. LAUGHTER

M- They last less time.

E3- And that.... Because of course, I'm also three years younger than him, not this time, but sometimes, I had my mother who died here, I brought her from town. My brother and I agreed to bring her to me and then my mother-in-law, my sister-in-law and I had her, she also died here.

M- So you have a lot of experience in taking care of her?

E3- And my father, he died in the hospital, because he had some little thing and died in the hospital. My mother couldn't and of course I was far away and I couldn't go to all the things they needed at that time and I don't know....

M- Have you seen any differences in yourself from when you took care of your mother or your mother-in-law to when you take care of your husband?

E3- Sure

M-Where would those differences be?

E3 -Those differences? I had a different strength then. When my husband retired, he retired at the age of 60, he worked at the Bank, well, they made him some proposals, he thought they were good and he retired and then when he had his grandmother, he sometimes told her "you do it, she is your mother", I took care of mine. And she would say to me "oh, well, you used to do it before" "but now you are at home, hey, help a little bit".

M-Yes, yes

E3- That's why, I think she doesn't have much willpower and of course, my mother-in-law, like...well, my mother wasn't here too long, bad in bed and all that. I don't know what else to explain to you. I've already explained half my life to you.

M- They are complicated situations

E3-Yes, yes, yes and when we talk about it sometimes with the neighbors or that, of course, then you were 20 or 25 years old.

M-Then you think that age and aging is fundamental because you don't do things in the same way.

E3- That is

M-You don't have the same strength

E3 -You don't have the same lightness and of course. I have had until not long ago, you know, because I like to go out shopping, to everything that is needed. But I really like music. And the concerts, to think that now concerts are starting in June at the XXX, in the gardens, I mean, he was not as bad as he is now, or if we, if I go somewhere, we have put him (today, my daughter did not open it for him because he is not very clear) 18'02 a railing, and I do not ..... Now, he is up on a chair. I mean, he left it and I went away, so I'm ................

M- Yes, yes, he had his moments of respite.

E3 -That's....apart, the doctor told me so. Because before, I used to go to town every week because he wanted to go and since I liked going to town so much..., we used to go on Fridays and come back on Sunday afternoons, to get less people on the road. I have always driven the car, because he said he couldn't see the colors well, I don't know what, I don't know how many ...., well; maybe it was a little bit of fear, or whatever. But I, because of the girls, because we took them far away, to "XXX" school, well, I got my license and I drove the car and...I still drive it.

M- Do you still drive?

E3 -I still drive

M- How old are you?

E3- 75. We went to town and we came back in the car too, my daughter and son-in-law came. They are behind me and they have adapted to the way I am going. We went to the spa a few years ago and we also did it that way. I would take the car and they would come, but when we came back, we would come back alone. Well, that was 5, 6 or 7 years ago.

M- How long has he been like this?

E3- Well, well I don't remember, but 7 or 8 years for sure, not that bad eh....

M- sure

E3 -So he's been like this for 3 or 4 years or something like that, and in the wheelchair I think it's been 2 years, not yet....how time goes by so fast, but, maybe, because I think it was around Christmas and it wasn't this Christmas, I think that's when we thought about the chair and that and of course... I manage pretty well; when I have him sitting up (and so far) he's been holding me pretty well, (gestures to the neck) I have changed him, I have lifted him up and sat him down.

M- Does he wear a diaper?

E3 -Yes, yes, yes, that's been going on for years.

M- And to wash him, to shower him 20'35 does he also manage well?

E3- Well, no. Some daughter or some other person has to be there to shower him (the truth is that I don't shower him very often), because it's not good for everyone. ....

M- Of course, if a person is washed in parts....

E3- Well, that's what we do. I, from here upwards (points to the waist) and when I have him up, I put a towel on him, so and so... of course I lift that (points to the genitals) more if... but I don't dare to shower him in the whole, I don't dare. And, well, about the doctor, he told me: that we should go to town anyway, because if he had wanted to go before, now (he was better, it wasn't like this now), and look, I still felt like going, of course, now it will be a little bit more difficult, if I don't recover a little bit more.

M- Yes, you will recover, what happens is that things need time.

E3- The doctor told me that: of course, between the arthrosis, it is older and all that, it will take longer, it is logical, he told me 10 or 12 days ago when he saw the .....

M- X-ray?

E3 -That and so on.

M- There are processes that are slower 22'19

E3- It worries me so much, as if it was already "pa" forever. You also said that with the neighbors, that we commented, you also thought that you would not recover and I am in the habit of saying: "not if I have to die before he does" (laughs).

M- Things of life

E3- Anyway, sometimes, some barbarity, (well, that is not barbarity) because there are many cases that are like that. That was bad or one or one that are sisters even....

M- Yes, yes

E3- The good one has died and the sick one has stayed and she has managed eh.

M- Things come out...

E3 -That's it.... And so we are, (she looks at the portraits) those are my daughters and my grandchildren.

M- How many grandchildren do you have?

E3- Four. If you turn around, you'll see, the oldest ones, look here behind .......

M- Ah, you have ....

E3- They are the same, the ones above and the ones below, as they know I like them (the pictures), that I take care of them myself. They did it to me in a Christmas this first one and in another Christmas those in kings. They know that it makes me a lot of fun and... they even tell me that I have too many, too many photos, and so on and so forth.... "Damunt que m'ho faig jo" some of them have been given to me, but others I have done them myself, they leave them to me, I do them to myself and I say: "they are to your children and on top of that ....". Those are the baby of this daughter who was here and who took communion last year and the other little one will take it next year, God willing.

M-He has a big family.

E3- All good yes, I have 3 daughters, the husbands. The two older ones only have one daughter each and this one (she points to the daughter who comes in the mornings) when she came out of childbirth she already wanted another one, yes, yes I say: "Mare meua que vergonya".

M- Wow 24'42

LAUGHTER

E3 -Woman, why do you come out a little bit "bitter"?

M- Well, yes

LAUGHTER

M- Do you have help with the family?

E3- Woman, if I need them, they come right away, but of course, since they are going to work ..... I have been very relieved about the .... button.

M- Teleassistance

E3 -That has relieved me a lot, because, first I call them (the Telecare) and they solve it right away, of course, the others (daughters) also come, if I call them. Yesterday or the day before yesterday I had been very sick for two days, so I called, in case they would send me to the doctor. Anyway, it took a while, it was diarrhea or something like that. It didn't happen many times, but, it made me so bad. I'm also a little "pachucha" (sensitive), because I got a little choked up from so much changing and so much .... (cries). A girl from the staircase came, I told her and she immediately went upstairs.

M- And do you have help at home?

E3- Yes, a girl comes once a week or every 15 days, because I care a little less (she refers to the cleanliness of the house) if it is neater or a little less. Before, it was more organized (looks around), but, if I take off (put away) the bags, I can't find the money or the keys, if I take off (put away) this (points to some diapers on the dining room table) "Mom, where is this or that?"......

M- You are adapting things to your needs.

E3 -That's right, and I feel bad (for her daughters) and I tell them "when it doesn't seem right to you, you fix it for me", then they don't find anything and I have it like this....

M- Have you always devoted yourself to your family? 26'37

E3 Always, yes, yes, yes, I haven't gone to work anywhere.

M- Well, at home.

E3- I think more so. It is another obligation to have to leave home than not to have to leave home, but ..... Work at home never ends. Now, their husbands, I see that they are different. Mine was a different way, and if something was missing on the table, "ro que falta una cullera, que falta açó un altre" (laughs).

M- I have lived this with .................. Things have changed a lot 28

E3 -Before, that's what you got married for, to stay at home and have children. Because I, my mother. My first daughter was born to me 25 months after my marriage, I had an abortion in between and I had already cried as much as I had to cry, because I didn't have children. And she was born 25 months after I got married. Then, I wanted (more children) and they didn't come. Well, then I wanted to stop and it couldn't be either (LAUGHS), because at 21 months one was born and 2 years after the second one, the third one was born. And, although my mother-in-law and my sister-in-law who lived nearby came a lot, even though the little girls were less than 4 years old, I had a hard time too, but well, you're younger and everything seems different...

M- It looks different

E3- Yes, it looks different. And, so we are 29'10

M- And you, now that you have your husband like this, what does it mean to you to take care of him? Why do you take care of him? Why would you say you take care of him?

E3 It seems to me that it is an obligation, the same as for the parents, it also seemed to me an obligation, it seems to me that it is an obligation. I told you, well, I started talking about you.

M- Yes, yes ma'am.

E3- Because when I see young people, it seems to me that I am younger.

M- You are doing very well

E3- AND .... And that.

M- We were talking about why do you care?

E3- I think it is an obligation and sometimes I do get angry a lot.... And I mean sometimes....

M- Why do you get angry?

E3- Well, what do I know, because I am like that, what am I going to do... (irritated) I don't want to get angry.

M- Will you get angry about something?

E3 -Oh, of course I get angry about something, because I can't move or I can't resist or I can't do it that much, eh....

M- Those situations, huh....

E3- I always think that of course, before, we used to do it. With him, we don't comment much because he is not very grateful, because, at least he would have a word....

M- Doesn't he recognize the work you do? You mean,

E3- He, not like now, but, he has always been a little bit kinder, more affectionate (teary eyes) and more of everything.......................

M- And now it all comes together.

E3 -It's all over (with anger) SEE

M- And you notice this

E3- So, this hurts you a little bit. But well....

M-.Maybe...

E3 -I'm taking away so much, so much, so much... I don't want to say the word....

M- And no recognition, not a word of encouragement....

E3- That's...

M- It's difficult.

E3- So, then... being quite well married, there are always little things, it has happened to me, eh, it has happened to me. But,... Some people tell me: "you have always done what you wanted". Practically yes... in the eyes of others. I have always gone out, he has never said anything to me. To go out to eat, if I had wanted to, I would have gone out practically every day. On Sundays, at least, if I didn't want to go out, because it made me very angry, to go out to eat and come back right away. "Xic, estem donant una volta" "Tu saps que no m'agrada mirar aparadors" "Però home, a mi si".

M- Has he always done what he wanted?

E3- Yes, yes, yes, yes

M- A little or a lot, whatever.

E3- A lot, but well, I've managed it pretty well 32'07. Because with other things I've had freedom.

M- And it has compensated you.

E3- And it has compensated me, so I, if I wanted to go where... he never gave me any problems, he never said, why are you doing this or why? He was a bit of a mess, yes, because I had to buy him clothes and everything, everything, everything, and I am a bit more conceited...... not to say much.

M- Well, maybe now you can understand why he "lets himself". Maybe he has always been like that and you haven't noticed him.

E3- That, as I practically did a little bit...Woman, now it is heavier and if you at least said that .... there are times, in conversations with a friend who also has a husband in town, she has Alzheimer's, well, you have to see. She is very much inclined to him, she puts the words before him. Because when I used to go to the village, my brother, this friend and another one would come to have lunch at my house and they had a great time. I would make a "cafenet" or whatever it was and we were all very happy and she would say to me "almenys Antonio de tant en tant em diu: si no fóra per tú, jo que faria reina" (Antonio from time to time says to me: if it weren't for you, what would I do as queen).

SILENCE

M - And that is appreciated

E3- That's right, that's appreciated and that he still doesn't have it so hard; God will tell eh. He wants to go to the countryside because we are from the countryside, almost everyone in town has a countryside. I'm not making too much of a fuss.

M- No ma'am, I am very interested in him.

E3- Yes? LAUGHTER I think I already talked to you about other things, people.

M- No, no, no

E3- That's why I'm telling you

M- No, because these things are related and I understand you perfectly well.

E3 -Yes, yes

M- And besides, it is very interesting what you are telling me because this way I can also understand what you are telling me.

E3- Of course. It's true, I don't add anything... ..... Maybe...

SILENCE

M- You don't finish saying everything, which is what I would like.

LAUGHTER

E3- No, but that's marriage stuff and you can't say it all....

M- You are right

E3- That we've had a few kicks, kicks, kicks eh, quatre crits, and... that's the end of it, in bed it's all over.

M- You're right, now you go out with these friends? 35'18

E3- When I go to town yes, and they come a lot. This Easter we haven't been able to go out. Before at Easter we used to get together, there was also another couple, and the husband also fell down and got hurt a lot, but he has a ..... Being a hut, not being a chalet, they have a very well arranged house, and last year we even went, we made a paella: the three days of Easter; one day paella, another gazpachos and the third, each one brought what he wanted. This year, between this one, that the wife already notices that she is a little bit ahead, she can't find the words; mine, that she gets like this (she gesticulates angrily), and that the weather has been bad... Because if the weather had been good, my brother was determined to take us all to ..... My brother is 6 years younger than me and we've always gotten along very well, very well.

SILENCE

M- There are also fewer occasions to....

E3- Fewer occasions of what?

M- For you to go there or for them to come.

E3- Of course, they also have it difficult. His (brother's) wife is very ill with pain, they have 4 children. The children go but, also, one girl lives in Valencia, another one... well, they work abroad and of course. I have been told many times: "you like coming here so much, why don't you come and live here", more people have told me that.

M- Have you ever thought about that?

E3- No, because I have my daughters here.

M- Would I be more lonely there, more helpless? ....

E3- That's right, I have a lot of relief with my brother, but ...., here I have. Now, with this (points to the telecare) this also works there.

M- Yes, of course

E3 -Other way of course, it doesn't work for me there.

M- Yes, yes, I would have to hire

E3- That's it. 37'46. But, I think that being here....even if I get angry with my daughters I say: "even if I died I wouldn't tell you anything" LAUGHTER, but of course, for sure, if I died I wouldn't be able to tell them.

M- Well, yes

LAUGHTER

M- Surely it is also difficult to ask sometimes for the help a person needs.

E3- Woman, I still have a little more freedom here, we get along very well with the neighbors. If I need something, I call the neighbors to take me to the hospital.

M- She has that resource which is also important

E3- Yes, yes and yes, my daughters come right away if something happens......although it seems that as we are solving the problem, I think they don't care a little bit, but well, I also see it as normal.

M- They are complicated situations. Do you call them or do they come during the day?

E3- No. If I need them, I call them and they come right away, but if the children have so many activities, they don't have enough time, and they have one each, both of them live in Xirivella. This one (the daughter who comes in the mornings) has 2...........pues more: "És que jo tincena mamà": Si jo ja él se....tu no vinges quan no pugues, no vinges...¿Dius tu de vindre?...... a mi de categoria....¿no véns?, ja m'ho buscarè jo per un altre costat.....

M- Do you miss that relationship with your daughters?

E3 -Weekends

M- With the grandchildren, what about them?

E3- The grandchildren...quite well, but ...... the grandchildren we took them. In the morning the parents to the nursery, at noon my husband and I, one in each cart, which took them next to the hospital, behind the hospital....

M- A school ....... The XXX?

E3 No, it was a kindergarten, but I don't know if it was....

M- I just don't know the area....

E3- Well, there was a kindergarten nearby...

M- You were in charge of...

E3- Of course, to pick them up at 12 noon, come, eat and then the parents would take them and in the afternoon they would also go 40'23. In the afternoon, one daughter would come, and they stopped working part time to take them, because if you say: "I don't see my daughter" "I have until 7 o'clock". The other one, she finished a little earlier, she is a teacher and she is in XXX, well, as long as she did not have children ...... 40'47

Conversation at the margin of the interview ...............................41'27.

E3- I have gone quite a few times (to her daughter's school, the teacher) because when the girls went out for something, for costumes, while they were little "Tots per a allí". The other one goes to La XXX and these others (she points to the photo) go, these two, the boy and the girl, go to XXX, which is on XXX street, as my daughter lives over there, so that's it. The in-laws live close by, the in-laws are the ones who pay the price.......

SILENCE

E3 -I, I have not been able to help them much, because of course, I was far away and this, well. One of the daughters, the one who is not the teacher, wanted to take her to another place, but I told her: "woman, since it is a school, what you want and it is similar to what you want, well, here". Well, and we have also been going for her while ....... Now, as she is older, she is in a bit of a hurry to go and she comes one day a week, because the other days she doesn't have enough time. From 1h she goes out, at 3h, to come to eat, being so close ........but no, because I don't know what she does from 1h to 2h or from 2h to 3h, whether it's English, gymnastics or I don't know what..... And the other one, she didn't go to the same school as her mother. Now she goes to high school, she is 15 years old in June.

M-They are older now

E3-This one, she still goes here to XXX, I think she can go until she's 18. And that's how we are. I don't know what else to say. 43'30

M- She doesn't think that everything she has told me is too little.

LAUGHTER

E3- No...

M- It's more than an interview, it's a chat.

E3- Sometimes these (telecare) girls tell me: "E3, when you are lonely or sad, call us and we'll talk for a while" and I don't know what and I don't know how many......

M- And do you do it?

E3- No

M- Why?

E3- Hey, because, I have enough to entertain me........ well look. Woman, I think that these things are not going to be told to everyone, and maybe they won't even have that much time....

M- Well, maybe when a person is like that...we don't wake up every day the same way, maybe chatting with someone....

E3- I'm a bit of a chatterbox, that's the truth.

M- Imagine that you need to chat

E3- Well....My husband doesn't talk at all, nothing, nothing, nothing "do you want this? yes, how are you? no, regular, or this" he doesn't talk at all...........

M- How do you deal with the fact that he doesn't talk at all?

E3-Oh, what do I have to do? (annoyed) if he doesn't talk at all? (irritated)

M- And you........ it's terrible

E3-I guess I've gotten used to it... he's always been a poor talker. My mother-in-law too. I've always gotten along very well with my mother-in-law, she almost told me more things than she told her children, for whatever reason. SILENCE: But they haven't been very talkative, they haven't picked on anyone either LAUGHTER.

M- Yes, yes, one thing leads to another: maybe you're right. But seeing you being so extroverted, you are so animated....

E3 -I, I'm saying it's something.... I have friends from the village and one day a week we also go out. Sometimes I spend some time chatting on the phone and when they tell me: "ro, tin paciència, açò o allò altre". It makes me feel bad because sometimes I get angry with XXX. I say: "tin paciència".......diuen, but, if I don't have it?..... My friends are also excellent, especially one of them, with an education and a thing that I think I have learned a lot from them.

M- How nice is that

E3- Yes, it is very nice

M- To be able to say that you learn from a person.

E3- Yes, yes, yes, yes, yes, because I see that I don't have that patience, I don't take it like they do, sometimes....

M- Does that help you?

E3- As I tell it, you can see that I calm down, I calm down a little bit and of course, I say: "that, it's very easy to say patience, eh" but if I don't have it or I've run out of it o....

M- Or things get over

E3- Of course

M- Things, from the outside, it is already said, that it looks very easy.

E3- Sure, it is very different

M- One has to go through it

E3- That's it

M- That's why I have told you that throughout your life you have had to take care of other sick people, your mother....

E3- Yes, yes. not that it was too much, not that so, in sickness they lasted too long. My mother-in-law died at 92; my mother at 84 and my father at 73, who died here in the hospital and it hurt me a lot that he died in a hospital, but then, of course, he caught something and was in the hospital and of course. My mother in the village, she was quite alone, although my brother went every day and so on. I think, although he did the same or better than me, but of course, he couldn't always be there. When she wanted to, she wanted to, she would come, she was there for short periods of time, first better and the last month, she didn't touch me, but my brother: "take her away, take her away, take her away". The doctor said: "it may last, but it may not last", in fact, it did not last long. After I brought her here and everything. It was not very heavy and it was not very long. And my mother-in-law, as it was my sister-in-law and I who carried it, well, neither. Besides, my mother-in-law was always delicate, but she managed, eh. What she needed was company, that's why we didn't leave her alone.

M- Sure 48'49 You think you have, not everything, because that is impossible, but do you have what you need to be taking care of your husband?

E3- What would I need? I, the only thing I need is a little bit of health, and that's all. ....

M- Do you have the strength to continue taking care of him?

E3- I, I have adapted to what I have always had and I have managed very well, always, before and now everything, I have managed very well. The situation is a bit heavy for me and not being able to go out LAUGHTER, when this happens (her fall) I think I won't be able to go out anymore.

M- Man, don't say that Rosa.

E3- Woman, I hope not

M- Even now you will go out, even if you are afraid, do you go out?

E3 -I haven't been going out for a few days now. My daughter brings me what I need, if I need it, the neighbors....

M- But why don't you go out? Out of fear, out of pain

E3- Because I am afraid. Woman, for example, if I'm lying down, in bed or sitting, it doesn't hurt...

M- It's fine

E3- Of course, that's what I think, but when I walk, it bothers me a little and of course, what I have to do, I would like the Lord to give me strength, so that at least I can go out even if I have to.

M- And you notice this lack

E3- I've always gone a lot and I really like going to the Virgin and all that.... And now I'm afraid to take buses, all that.

M- The truth is that when .... There are people, well, it is written that when people fall or feel that they could fall, that fear makes them not to go out.

E3 -That they don't go out, yes, and besides, I think that sometimes people say "Look how good it was, if it hadn't happened because of the fall".

M-. Yes, but this can happen to anyone.

E3- That is, well, as I see that it lasts so long, because although this (another fall) also lasted, but well, I did go out, although I didn't leave the house for 15 days when this happened, they told me: to do absolute rest, not to move my arm, to put ice on it, and now... as I see that I am not better, if I want I go to bed for a while, I put the TV on and that's it, and between the scholarship and scholarship...

M- Do you rest well at night?

E3- Quite well

M- That is also important

E3- I wake up very early, go to the toilet and that; I go back to bed, I don't want to get up early because I'm better there (in bed) and since my daughter or the girl is coming, I don't get up early.

M- Very good....... 52'39

Farewell conversation with no interest for the interview.

**E4**

E4 -I was born in 1921.

M: My goodness, my goodness. Well, you look great for a 90 year old man.

E4- From Cuenca. My wife is 83, she's an invalid...

M - Yes, XXX told me. Is she always in bed?

E4 -No. We lift her, she can't walk, nothing, nothing, nothing, nothing. We have a crane to lift her and we lift her every day, but she doesn't do anything... or anything.

M: How long is your wife like this?

E4- Well ..., like this, as she is now, because she has "been" with crutches at the beginning, with carts and we have a junk storage room ...... So, she has been like this for 6 or 7 years. And now, at the end, he can no longer walk, nothing, nothing, nothing, nothing, nothing, nothing, nothing, nothing. She is always in the wheelchair; we lift her up and she is here all day long (in the dining room where she receives me) in the chair and then she lies down .... and she is there. You can't have it, we have "probao". I sometimes tell her "let's see" (with her head she gestures denial).

M - And there is no way

E4 - And there's no way he can stand upright 2'13

M: Do his legs fail him or does he have no strength?

E4 -The legs, they don't have it?

M - Yes, yes

E4 - They can't hold her up...

M - Do you have help to lift her....?

E4- Well.... are, my son and my daughter. 2'42 .I have this lady who lifts her, she comes until noon this lady and lifts her. That I pay her 400 euros and she lifts her every day. Then my son comes to put her to bed this week; my daughter comes the other week....

M - They take turns...

E4 -Of course, to put her to bed, because they are all working, and they have their own .... it can't be. They can't take care of her. They can't take care of her like that, just come at night to put her to bed. He is here this week and the other week, the other week and nothing else, they cannot be here like this lady is here all day long, for all the things that have to be done, cleaning, food, everything; I have to have a woman, which is this lady I have here 3'15".

M: Does the lady do the cooking?

E4 -Yes, yes

M - Does she also feed your wife?

E4- She eats all by herself, yes, she eats all by herself and ..... She wants to eat a lot, thank goodness, otherwise she would be already.... But she really wants to eat, that's what she wants to eat, otherwise I don't know where she would be already.... Va.... well, what can I say. Me, I'm already "bored" of so many years that we have been....

M - It's just that it's very hard

E4- Ya, ya, ya (resigned attitude)

M - Do you go out? He goes out of the house, he gets distracted

E4 -No, nothing, I can't go out. Well, I go out every day to buy bread, things, to the doctor, to the pharmacy. To the doctor and to the pharmacy half the time....

M - Every day

E4- And then, in the afternoons, since my wife is alone, well, I have to be here with her, that is, I don't go out. I haven't been to the center of Valencia for 3 years.

M: And do you miss it?

E4 -I used to, of course. Besides, I had a little "pequeñusa" dog that we both used to go out. We used to go everywhere, downtown and I ran around a lot with her, but .... Now I haven't been able to go out for a few years, no, nothing, I don't go out of here (points to the street) 5'55

M- What happened to the dog?

E4 -It died, it was 17 years old.

M: Ah... well. Because for dogs that's a lot of age.

E4-Man, of course, she died very old. She died here .... -It's been 2 years since she died.

M: Besides, they keep you company, don't they?

E4- Me, I didn't know how to go around alone.

M - Sure, I would notice the lack and you haven't thought of having another one?

E4- No, because I'm already old and... (He gets up to take some things from the table).

M: Are you going to eat now?

E4- No, no, it is soon....

M - Since there are people who eat early ...., what kind of work have you done E4?

E4- I worked as a carpenter

M - Always, as a carpenter

E4 -Always in woodworking. In my town, I worked as a sawyer, always in wood and then, here in the carpentry shop, because I was also a carpenter in my town. As I had a friend there who had a sawmill, so: "you can come and saw with us". He was my wife's uncle, and then, of course, I married his niece. So, I have always worked in wood and then when I came here I went straight to a carpentry shop. And I've been here in the carpentry shop for 18 years.

M: Wow 7'54, is your wife also from XXX, from the same area?

E4-Yes, from my town

M - Ah from the same town

E4- Of course, we got married 60 years ago.

M - My goodness, E4, of course that's a lot of years. LAUGHTER

E4- Sure, 60....61 years "married" to the same woman.

M - Well, that's good

E4- Not like now. Well, I've been married for 61 years and nothing happens.

M - Have you always lived here, in this neighborhood?

E4- Well, no. We have always lived there, on XXX street, it's there (points to the address).

M: In this neighborhood?

E4 -Yes, yes. Then, it turned out that we couldn't get out with our wheelchairs because the elevator was at the bottom of the floor, there were stairs we couldn't go down. We were always stuck there.

M - So, before she was 6 years old, your wife was already in a wheelchair?

E4- Yes.... I'm already lost

M - She was telling me that they lived on XXX street and they couldn't get out of there because she was in a wheelchair....

E4 -Yes. We lived here for 3 years. I couldn't get them to put the elevator all the way down because the people downstairs didn't want it...

M - Problems with neighbors

E4- The landlady downstairs, which is where there is an ambulance park. In XXX street, there is an ambulance park .... there, well, she was not to blame; it was the fault of whoever was renting, in the end, the property did not want the elevator to go down. As you can see, they didn't want it. And there were some steps that had no way, because if there was a way to put a ramp...nothing, there was no way. I took several of those who put the devices there to go up and they told me it was impossible.

M - There was no way

E4- There is no way to put it, no way at all.

M - And then what did they do? Because of course with the wheelchair, they couldn't get out, couldn't they?

E4 -No, no, we couldn't go out 11'29. When we had to go to the doctor sometime, my children had to come and together we would take her out. It was a big mess. So, we said, nothing, we had to look for a place where we could be. It was nowhere to be found, no....but we found this. My daughter lives across the street.

M - On the same landing?

E4 -Yes, yes

M: Oh great.

E4 -There was an old lady who died. This apartment was going to be sold and my daughter told the owner and he said: "since you're not going to keep the apartment, anything you want" and, have you seen that there is a ramp? Great.

M - Yes

E4- That's why we came, of course.

M - After that experience. When you change, it is to improve if you can.

E4- Otherwise I would not have changed.

M - Of course

E4- That apartment is mine and this one is rented.

M - Ah, it is rented

E4- Of course, this one is rented, but I have an apartment there, 40 years.

M - Without inhabiting it

E4- No, I have it "alquilao", at least it helps me to pay for this one.

M - Yes, yes

E4- I bought the apartment when I came here, I have been in XXX for 42 years.

M - That's years.

E4- And I was 45 when I came here, I was already old.

M - Yes, ...., you were not young when you came.

E4 - Of course, I'm 90 years old....

M - So when you were working, was your wife already sick? Or did it happen when you were working and your wife was sick?

E4 -My wife, at that time she was ill, but she would go with her crutches, she would make me lunch and so on... and we would go out. But when things got so bad, the ....... We didn't put her in a wheelchair until 1 or 2 years before she came here; when she was in a wheelchair and couldn't .... she was sick for at least 10 years, or more. But, with the crutches she was fine and with the walker, they sent us a walker there in the hospital and she walked and did things....food and so on. He defended himself that way.

M: Of course.

E4- Then it was already the thing that could not be....

M - What happened to him?

E4 -He has... everything you ask for. They do an analysis and nothing is right.

M - There are times when so many things come together.

E4 -He has everything, he has a test and nothing is right. Well, she's had two angina pectoris, well....

M - She is a strong woman...

E4 -She has .... I don't remember....

M - Don't worry

E4- What's her name....

M - Don't worry, the most important thing is that she can't move and it's all on you....

E4- You have.... I don't remember, it's the worst thing, I don't remember now

M - Well, don't worry

E4- My memory fails me a lot, it's the only thing that fails me the most.

M - It will come later

E4 -Memory fails me.... arthrosis. You have a horse arthrosis.

M: Sure, and arthrosis gets worse and worse with time, and if the person doesn't move, it's even worse, it's a vicious circle.

E4- I don't know how she is in this world, I tell you, she has had 2 angina pectoris, she has had .......

M: Is she conscious?

E4-Yes, yes, she is failing already (points to her head).

M - Well, but I mean, ....

E4 - Because if she doesn't go out anymore....

M - What is your relationship with her like? Do you talk to her?

E4 -Yes, a little, but yes. Now afterwards, we pick her up and I am here. This lady picks her up, gives her the food and leaves. She leaves the food ready for me and leaves. I stay here with her all afternoon, until my son arrives to put her to bed at night... and we are talking, but she goes out of her mind.

M - Yes

E4-She's gone deaf and her eyesight is failing her a lot, anyway....18'12

M - How are you doing?

E4 -I am

M: How are you handling the whole situation?

E4 - I'm fine...

M - But, well... What do you mean?

E4- Man well....(irritated) I'm not handling it well, I'm handling it well because I have no other choice.

M - I understand perfectly well

E4 -There is no other solution, but, I don't take it well, of course.... if it could be better, or in another way....When we were 30 years younger and we lived so well, but now....then....of course, but....I take it well.... And so here we are.

M - Do you have help, apart from your children or, do you think you have enough help with your children and this lady?

E4 -Yes, for help yes, for what she has to do yes, because this woman fixes my house well every day, cleaning, food, everything and...she washes, in short. She is a very good person and very clean, very hardworking and... this is very good, I don't mind... Since she is leaving here there is nothing else to do.....

M - That's when you need to go out more....

E4- Sure, sure...

M - The other thing seems to be more taken care of...but, the afternoon....Additionally, the afternoons are long.

E4 -Well, here every afternoon with my wife.... I tell you, it's been at least 3 years since I've been to the center, at least 3 years since I've been to the center....

M - And friends, do you have friends?

E4- Yes, of course we do

M - And... do you see them, do you have a relationship?

E4 -Yes, yes, yes, yes........ Yes, lots of friends....

M - That's good too...

E4- Sure, and in my town....

M - Still going to the village...

E4- Well, we haven't been going there for a year now. I've been going to my town all my life, because I really like going to my town, I like the town. Every year I have gone, all, all, all 21'01. Every summer. We haven't been going for 2 years now....

M - Of course, moving his wife and everything is...

E4- But to go we have to take that machine with us, the crane, how can we take it? We can't go anymore. I really want to go to my hometown. ....

M - At the moment there is no possibility?

E4 - In my town I have...man, family...yes, I have nephews and nieces. Here I have a sister and I have a brother in XXX. My wife has a sister there.

M: There in the village?

E4- She is 91 years old and she is better off than she is.

M: You are long-lived people, mother of mine.

E4- She is better than her... I like to go there. She has a family there because my wife grew up with an uncle, she had no mother when she was 2 years old and she was raised by an aunt from the village who .... was a lumberman and they only had one child. So she took her and raised her like a daughter until she married me, that is, they live there. The children, because the older ones are dead, the children live there, well the son because they only had one child, but they have very good relations.

M: Is the relationship still going on?

E4 - Yes, yes, yes, yes, even though they, of course, they have a lot of money, .... They have a son who is a general of the civil guard... who was here the other day and so on, We have very good friendships, from always, and we keep them. We have very good friendships, from always, and we keep them, friendships that appreciate us a lot, by the way. Of that we are well ....

M - Very good 23'38

E4 -So...

M - Do you have grandchildren?

E4 -I have great-grandchildren....3 great-grandchildren

M - Of course, I have to ask you about the great-grandchildren, because... the grandchildren... LAUGHTER

E4- Three great-grandchildren, for the moment

M: Are they coming this way?

E4-Yes, of course they're coming. Yesterday afternoon we were all here. I have a granddaughter who has 2, she is a dental mechanic, she and he (her husband), they are both dental mechanics, they have a laboratory here in XXX Street.

M - Yes, yes

E4 -She has 2 girls. I was with them yesterday. And I have another grandson who was also here yesterday. They come a lot, a lot

M - This is also a joy, her face lit up when she was telling me about the great-granddaughters....

E4- Man, of course

M - It is a luxury

E4- I have 5 grandchildren. My son has 2 daughters, none of them are married yet. One is in the XXX and the other is finishing nursing 25'25

Conversation not of interest to the target, enjoys talking about great-granddaughters and showing pictures.

E4 -I could not give to studies, (to the two children) then they said that it was better the man than the woman, because....

M -Women stayed at home to take care of the children....

E4- In those times it was like that... we are talking about 40 or more years ago. My daughter also conformed, then her brother studied and she was there, at home. She has a degree in hairdressing, tailoring and dressmaking....

M - The things that were done.

E4- Then, here in Valencia she was in charge of a very big store, all the time, until she got married. I was here.... At Almacenes XXX....

M - Yes, I remember Almacenes XXXa....

E4- Of course, XXX's son-in-law was a partner of my wife's uncle, the lumber dealer, he was a partner and he was there for 3 years, in my town, there with him in the business. He had a lot of business and I came for him. What happened is that I didn't come here before, I should have come here before....

M: Why?

E4 -I was fine there. I was living well with the salary I had, I was living very well there....

M - Sure, it wasn't coming to the city to look for a job....

E4- No

M - And why did you come?

E4- Because my children did not want to stay there

M - Ya.... children drag and the possibilities for the children...

E4- But well, I, if it hadn't been for my daughter who is the one who....Was in XXX, with a niece, well my wife's nephew. She was a teacher, and she took her to take care of the kids...she didn't earn anything, they didn't pay her and she was there in XXX for more than a year. So, she also went there..... But I didn't want to come and then..... She didn't want to be there 40'21

LAUGHTER. In town, she had her friends, well, and she was thrilled....

M - But.... When you meet other things...

E4- Of course, when she got to know that, already... and that's what happened, people started to leave the town.

M - Of course...

E4- And the town was left alone.

M - I think that this has happened in many towns. Young people have been leaving.

E4- And I didn't want them to be alone and that's why I came, because of them.

M - And they came

E4- If they didn't, I wouldn't come. I was wonderful there, I was fine. I didn't miss any work, how could we miss it?

M: Often in the village there are more resources than in the big cities.

E4 -Of course

M: People know each other, it's a different way of life.

E4 -I was very good there. I had very good 41'33 friends, and in short, we were very well, me and them.

M - A big change

E4 -I never wanted to leave before. They were looking for me, they often came from XXX 2 or 3 times.

M - To work

E4- Yes, to work at XXX. They looked for me 2 or 3 times. Here (in XXX) 4 or 5 times. They insisted "you have to come, so and so, "we are going to give you a house", they gave me everything.

M: And he didn't leave.

E4 -I never came, neither here, nor to XXX. In XXX I had a relative who had a very big carpentry workshop, he used to say "you should have come here with me, I need you here" He had others too...

M - Yes, of course, I can imagine, but he loved you, of course.

E4- There was trust, we were relatives

M - Of course, and because his work would be good for him.

E4 -Others, they were not relatives or "na", but they insisted "you have to come here". I was in XXX for 28 months, with the military service. It was very cold

M - Now that the military service is no longer done 43'10

E4-I spent 3 and a half years in the military service.

M: That's a lot, well, at that time you would do those years, wouldn't you?

E4 -I don't know. That was at the end of the war

M: Of course, we are talking about a long time ago.

E4- Sure. The year 42.

M - I was thinking .... I wasn't born yet. I am 55 years old RISAS, mother of mine......

E4 -That's my son's age.

M - And you're talking about when I was in the military in '42....

E4 -3 and a half years of military service, mother of mine. I was in XXX for 28 months. Then, they took us to the border, because there were the maquis, there was a thing.... You see, we were in the mountains, they took us there. I was very well (in XXX), I was wonderful, the bad thing, so long, but I was very well, well, well. I was also well "prepared", of course, not only in carpentry, I was well "prepared".

M: From life? Experience?

SILENCE

E4 - Of culture...

M - That is very important

E4- Man, of course. I (before the war) was also studying, but during the war, everything was screwed up... I didn't get anything, nothing. The teacher told me "you're going to get 2 years in one", that is, I was well "prepared". In XXX, (militia) as soon as I arrived I was already a corporal, not because they knew... but they made me a corporal.

M: You could tell

E4- Of course it was noticeable. If I had stayed in the military, as they wanted me to stay, I would have "made it" to....

M - Of course, by moving up... military career.

E4 -I had a career, but I was very good there. I became a Corporal, I didn't want to be more; I could be a Corporal in the carpentry shop and I had no service at all, nothing....

M: And what did you do there?

E4- In the carpentry shop, I was working, but without working. I was the Corporal and I had 5....

M- You were in charge

E4- Sure, I had 5 carpenters under my command LAUGHS

M - Of course

E4 -I worked because I liked it. So, I had to go to the academy to become a corporal. First, it makes you an acting corporal, then you have to go to the academy. And I went to the academy, little time....joder, there was a bunch of clumsy people there...I knew more than...and the Captain who was the teacher told me "come on" that I couldn't "yes, yes, how can you not, of course you can, you leave everything". I went one day and I told him "Commander, why am I coming here, tell me why am I coming here? What are they going to teach me here...if they are all a bunch of sheep...forgive me, but, poor things, they didn't even know, not even .... when he said "look, don't come here" 6 months at the academy.

M - That was a waste of time

E4- When they gave me the exams, of course, I got number 1, without having gone to the academy or anything.

M - Of course, I was above. Are you a conformist?

E4-Yes

M -Because you say "it was good" "in the village it was good".

E4-Yes 49'02

M - When you were talking, in relation to the care of your wife, you say: "well, it was fine".

E4- Then, I was also a practitioner.... when they took us out of XXX....

M: And that, did you also master it?

E4 - Man, I was a trainee for 6 months RISAS

M - And did you work

E4- No, I didn't work

M - Well, did you do things as a trainee?

E4 How? of course, every day. There was no one there, I was a first-aid corporal and I had 4 trainees under me. I always, of course, to the chiefs, the others (who needed the service of a medical officer) could not demand, that one had to be pricked, or do something, they would say "let the Corporal come".

M - They wanted you to go there LAUGHTER

E4 -I was really good at it, it seemed impossible to me. When the sergeant sent me to the hospital for tests. They had everything there and my sergeant said: "when you come, you will prick me" LAUGHTER. The first one I pricked, besides, it was intravenous, the injection I gave him.

M: Yes, yes, damn it.

E4- One, I had put, intravenous. Because, in the hospital (internship) they took us every day to see them put in and then, we did the tests in a cotton package....

M - Of course

E4 -People were never left. And me, an IV, "na más" que llegue. Four trunks full of stuff LAUGHTER ...... I had a wonderful time.

M - Also the things you have done if you have liked them... that is important, if you have been recognized...

E4 - Yes, yes, of course

M - It is fundamental that a person is valued.

E4 -So, I did everything in the military..............

M - Now, when you have to do something to your wife, you don't make much effort, or what?

E4 -No, no

M: You, do you master it, or not so much?

E4 -Now, I take the sugar, the tension..............

M: What is more difficult for you, are the strength things, because you can't, of course?

E4 -Yes, we can't with her.

M - But, it is not new for you to do other things, with the experience you have.... What does caring mean to you. Taking care of your wife?

E4-Well, it means a duty that I have to do and nothing else and I do it, because she is my wife and I have to take care of her because she is my wife. And, I'm comfortable with that

M: Do you take it well?

E4 Well, how can I not, if she is my wife?

M - Well, but maybe these things. You seem to be a very cheerful and optimistic person.

E4- Precisely....... me before going to the military if I saw a needle prick I would faint...and then..... as I got used to it....

M - That could be, people get used to it in life..................

E4- Of course, then, you had to do that because otherwise you had to "pringar" a lot, there in the military. You had to "pringar" and before that, with all the fear that it gave me.... I did it. Because of course, there I lost the assignment of XXX, I was a carpenter, but the lieutenant said: "the first assignment I'm going to give you" and then one day he came and said: "I already have an assignment for you, I don't know if you'll like it or not".

M: And did he like me?

E4 - I liked it, because I liked anything, as long as I wasn't in the military service.

M - There must be something in people and you have many years to know it. There are things that maybe we don't like to do and we do them.

E4 - He says to me: "practitioner" LAUGHTER "you are going to take 4 soldiers and you and you go to the hospital to do the course, you have to be there for 6 months" I say: that's what I like the most...... What could I like! I didn't like it.

M - But you did it

E4 - I didn't like it, but I had to do it, because if I didn't ..... I had to "pringar" and there, there were things that seemed to me impossible that could happen....

M - What can there be inside, (in this case of you) to overcome that? Because, all people are not the same. What do you think it is that you have, that has made you overcome problems (you must have had a thousand problems, like all people), what has made you overcome them? Or difficult situations that you have overcome? Because I am watching you and the feeling you are giving me is that things have been going well and you have been adapting, adapting. But, there has to be something inside you to overcome How do you overcome these things?

E4 - I ..... with willpower

M - Willpower? 56'21

E4 - A lot of willpower

M - There must be something in people

E4 - I had a lot of willpower

M - Why do you say I had? LAUGHTER

E4 - I had and I have

M - Ah, I don't know

E4 - I still have it, yes

M - You must have something, because you have a complicated situation and you overcome it, that's because there is something....

E4 - Yes, yes, yes, yes

M: Because, I don't know if you remember, but the first thing I asked him was, "How are you doing, how are you doing? And he said "fine, oh, how am I doing? fine".

E4 - Of course

M - There must be something there

E4 - Yes, I mean, I'm doing badly, very badly, very badly. ....

M - Well, you could have said it and still be the same, of course. A person can say: "I'm doing very badly, but I have to do it", you know what I mean. On the other hand, you seem to be optimistic

E4 - Yes, yes, I have a lot of willpower.

M: That willpower is what has made you overcome the things in your life.

E4 - And it has always made me live well. For example, I was here in the workshop, they threw a lot of people out because there were 50 of us in the workshop.... then the system changed, they brought in a lot of machines.... they threw a lot of people out, they didn't need so many people.... I was the last one who had "entered" and they threw people out, people, people, people, I already had another workshop, the boss was a friend of his (workshop boss) and he told me: "if they throw you out, tomorrow with me". They threw people, people, people...they threw everyone who was there before me and they didn't throw me....

M - And maybe you never thought they were going to throw you....

E4 - No, I didn't think so....

M - Many times being an optimist or having that vision of life helps.

E4 - I, well..... was calm, because as I said, there was another one who had a very big workshop and had many workers and he also told me: "don't worry, if they throw you out, tomorrow you'll be back.....".

M - You already have a job....that also gives a lot of security....

E4 - But how could that one throw me away....?

M - Of course not, because the other one also knew what you were worth, of course, he didn't throw you, how could he throw you!....

E4 - When I retired, he (your boss) didn't want me to retire at all .....

M - Did you retire because of your age?

E4 - Yes

M - At what age did you retire? At the age of 65.

E4 - Yes.... and he would tell me "you are here until whatever you want, 70, 80, until whatever you want".

M - And .... Why did you retire?

E4 - Because my son and my daughter told me "you already have enough to live on, why do you need to be working". ....

M - The pension you receive is more or less 1000€.

E4 - Less than 1000 €. Well, I retired at the age of 64 and they took 8% from me. I wanted to, because they are very heavy chores .... that are not .... if it were another type of work, but that XXXXX and with my machine that I carried it like "na", but my children, they insisted "that you already have enough to eat, man. With what you have left, you already have enough".

M - He says "for what he has left" and he is 95 years old LAUGHS

E4 - Sure, they thought I was going to die right away LAUGHTER. The boss didn't want me to retire at all, "with the mess you're going to make for me", "but how, they're going to retire you, but you're better off than me and you want to retire". .....

M: So, you have also done many things for others. For your children, they came here and for your children you are retiring.... and now for your wife, also taking care of her....

E4 - Ea

M - That's right

E4 - Of course...

M - That's life eh....

E4 - But come on...me, life has always been good for me. I haven't had any problems, well, yes, problems, but...not about work, about earning more money than others...because I've always been a first officer, in all parts .... I wasn't a laborer, I had, besides, this one, the one at La Cadena warehouses, paid me more, that is, as a tip than as a salary. When I came to the office he had at .... he was always in the office: "E4 pass by here", he passed by there, his name was D XXX. I would say to him: "D XXX I have already "cobrao" RISAS and he would say to me "here you have this" he was giving me for 3 years that I was there. He was giving me tips. In other words, I have always been fine. I've "been" very well "mirao" and well cared for, in short, I've "been" well, well, well, well, I'm not complaining about life. 1'03

M - You are grateful

E4 - Here, too (there were two brothers) and one... left, they didn't get along. While he was there, the same thing happened to me as in XXX, we would arrive to get paid and he would say to the clerk "give 1000 pesetas to E4" SILENCE and I did nothing at all for ........................

M: You think I did nothing and I was a fundamental person where you were.

E4 - So, yes, nothing, I did nothing. Now, I did what I had to do, my job, nothing more, nothing less.

M - Well, I would do something different from the others. Because if people love a person, that's for a reason.

E4 I remember one day the boss came down to scold me, because I was taking too long, I was sorting the wood.....y came down one day to scold me, he went with the officer, both of them, he said "E4 you are wasting your time" because, because you have to sort the wood because otherwise it is a mess, one color, another color. I don't do something wrong knowingly, I don't do it, I don't do it; when I do something wrong it's because I don't know how to do it. That's how it is, it is classified because if I have to make a mold, I have a ribbon of one color and the other of another color. And he said to the carpenter "E4 is scary" and the other one said "everyone has a way of working". Well, when he left, he said "you know what I'm telling you, that you 1'06..............

M - In the end E4, you are the master.

E4 - I, I have not pretended....

M - No, but they have gone like this....

E4 - I, I wasn't looking for, they came out like that. Here, like in the military, I wasn't looking for anything. In my town the same way, all the people wanted me to go to work in XXX to XXX........ and I didn't do anything.

M - Yes, but you as a person with your way of being....

E4 - Yes, they informed. In XXX, a man came and told me that I had to go with him "but well, who told this man who I am....". And of course, they told me "they asked us"....

M - Yes, yes, yes, yes.

E4 - And we told him ..... I mean, I didn't care about anything.

M - Or it seems that you didn't worry, but, you did things as you thought you had to do them and that was good for you, your character, the way you related to others....

E4 - I was fine, fine, fine with everybody. I have been several times in charge, also in town, quite a long time. I was there, as a woodworker and things.... they sent me. Anyway, but I wasn't looking for anything, I didn't say I was going to look for this so that ..... would find things. Some people are looking and can't find.

M - All day long in a bad mood, angry with everybody.

E4 - And I, however, they came to me without looking for them.

M - Maybe the key is that the way we are living, we have to be satisfied with what we have. Maybe that is also important, I don't know.

E4 - Anyway, and I am living well until today, to say the least, I....

M: Are you satisfied with the way you are now?

E4 - Yes, because I must be ..... with some things, others not, but....

M - How do you see the future?

E4 - That.....RISAS with 90 years RISAS

M - Don't tell me you don't see the future.... with the age you are and the way you are, don't tell me.

E4 - Me, I don't worry about anything anymore.

M - Live in the present.

E4 – Yes

M - And try to live the best

E4 - Sure, I can live it......pues I live it.

M - Of course you can.

E4 -I live without problems, just the way things are..........pues me, I live without problems. I don't have any problems.

M - And the more everyday things are also fairly under control.

E4 - Nothing, I hardly think about anything. From here, any day I'll have a syncope and I'll go "shitting my pants".

M - I don't know, if you are going to have a syncope....yo from the outside I see you very well.

E4 - I don't think about anything, I'm 90 years old, I haven't turned 90 yet....I'm going to turn 90 on the day of E4, look what I'm going to miss.

M - Nothing

E4 - Since I was born on E4's day, I was named after the saint.

M - Yes, it was customary

E4 - Anyway, my brother who died last year was already 94 years old?

M - Your parents died very old?

E4 - My mother died when she was 92 years old, my father died earlier. My mother, she was here with me several times.....because she was with her other children. There were three of us here, my sister, another brother and me; and in XXX, there were two, we were five siblings, four brothers and one sister 1'13 She was all over the place. She was better than my wife. I used to go out with my wife and she had to take her, instead of my wife taking her....otherwise, she took her.

M: Her mother drove her wife. There are people who have very fragile health and others....

E4 - that's when I didn't think my mother was going to die. How she died.....si was fine. I don't remember what happened, nothing, she died.

M - So, suddenly, very quickly?.....

E4 - Yes, very fast, without illness.

His wife calls him from the bedroom

E4 - She is already calling for us to clean her up and get her up...... she is deaf too..... we get her up together (the housekeeper and him).

He calls again, the housekeeper comes, E4 answers but his wife does not hear him.

E4 - Now we get him up between the two of us, this woman (the housekeeper) does all the things, the food and so on, and after getting him up and so on, she leaves and ......... and I stay here with her (his wife).

M: And then, what do you think when you stay with your wife? 1'15

SILENCE

E4 I..., well, nothing. This lady (the housekeeper) is a very good person.

M - She is an important support for you

E4 - Yes, she is

M - The housekeeper is not a resource.......... you pay her....

E4 - I pay her 400 euros every month

ASSISTANT - How long until the survey?

E4 - No

ASSISTANT - It's just that I finish the dining room and then we clean her up and get her up.....1'16

At this point it seems that we are done but E4 continues talking and provides some information that I pick up. She wants to keep talking, she comes and goes from the bedroom without stopping or being able to say goodbye.

E4 - My memory is failing me and I think this is going to end soon. Anyway, we are here for whatever comes up. 1'18

1'20 He comes back to his life experience.

1'21

M - Would you say that you are alone?

E4 - No... I am not alone, with my friends, my children and everything, no, I am not alone. I'm well considered, well cared for, so I'm not lonely, no. I think it's because I have no friends, no children, no "na", I think that's what it is, or that I'm lonely, I think. ....

M: Don't you find yourself alone?

E4 - No, even with neighbors, I mean, I have no complaints from any of the neighbors here. Where I was for so long, they appreciated me, all of them. Besides, just being the father of XXX.... (XXX is his daughter who lives in the same farm).

M - XXX, who does he look like?

E4 - LAUGHTER, He looks more like my wife's family.

M - I mean, let's see if he looks like you.

E4 - Man, he must have something

The maid comes to ask for help, they go to clean and lift E4's wife 1'23. When she returns, she continues to talk about her life, repeats many experiences I do not turn off the recorder 1'26. She has no chronic disease, she tells about her state of health and the interventions she has undergone for cataracts and prostate. 1'29 talks about his wife's health. 1'34. 1'53 The woman enters in a wheelchair accompanied by the assistant and E4, a conversation is held that is not important for the purposes of the interview.

**E5**

M - You retired to take care of....

E5 - I retired at the age of 64, but of course .... I retired because .... I would have continued for another year or two, but I couldn't because of her. I took her to a center, later, because she, first it was Alzheimer's, after Alzheimer's, she was already in bad shape. It was already the second or third year of Alzheimer's and she had a general cerebral thrombosis and then we had to bring her home. They told us to bring her home or take her to XXX...so XXX...... how to abandon her....well, not abandon her, but.... how to leave a sick person there, so far away, do you know where XXX is?

M - Yes, yes

E5- Taking her so far away would have been a problem also for us and for her, because he "took care" more or less the same. So, we decided to bring her home and here we are taking care of her, we are doing what we can. Now she has a fever, it is probably the sores that are causing it, because they have become infected. So they are causing her fever. Or that she is constipated, we don't know.....constipated we don't know if she is or what....

M - How long has it been....?

E5 - It's been four years. Now at the beginning of the year, it will be four years. 1'52

M - Gee, so the reason you stopped working was so you could take care of her?

E5 - Sure. 2'03

M - Do you have children?

E5- Yes, two daughters. They both work, they are civil servants. Well, one is a teacher and the other one works at the INS and of course, they are not going to leave their jobs to take care of her, that's for sure ......

M - Yes, yes

E5- I, I admit it, they are not going to stop working, just because of... and of course it has cost them a lot to get....

M - Of course

E5- And there we are, so that's why ........ That's why I have so many obligations, because, normally in the morning I only have two or three hours and ....... one day I have, for example, to go to the bank...... I have to do some business, so of course I take advantage of it......... (To do that business) in the afternoons I am more free, I am at home because my daughters are not at home either, they leave....... they leave, they practically leave it to me and that's it. The older one has to leave precisely, the younger one, she is married, of course with two children (a daughter and a son), how?..... she cannot take care of this house; she has a ..... she works too, so she cannot, impossible....

M - Do you have domestic help?

E5- I have a lady...... I pay her 3'26 eh...she does not come from any center.

M - What does she do every day?

E5- She's there in the morning, I can't afford to pay a lady all day long...

M - Sure, you have to value everything. What is a typical day like for you? What tasks do you do?

E5- The tasks are, worrying about giving him water, medication, dinner...in the afternoons of course. In the morning, she takes care of Mrs. ...., the snack, dinner and putting her to bed because she is catheterized too, she has a catheter (she has a nasogastric and bladder catheter).

M - Is she mobile?

E5- Nothing, nothing and so I am.... I am, because if she has fever, if she has no fever, if her blood sugar goes up, because she has a lot of sugar....now her blood sugar has shot up, we don't know what to do eh....

M - Of course, the body becomes unbalanced....

E5 - Last night, she was at 244 (blood glucose), what do we do, I prick her, I don't prick her.....I don't know what to do anymore. I can give her a rapid insulin "Humanol", I think it is, well..... "Novo Rapid" the Humanol is the other one.....I have a doctor's order that from 250, I gave her a shot. But, of course, it's 254..........but let's face the night. She had a terrible night, surely, surely not... at 2:30 in the morning I tested her blood glucose again and she had 230 or thereabouts, I can't remember exactly, I didn't prick her. He was sweating all night, complaining .... and so on. I don't sleep, I practically don't sleep, half the night I don't sleep, I don't rest.

M - And then during the day

E5- During the day it is quieter, yes. From 2 p.m. onwards, we fix her, this lady, gives her food, medication, gives her everything and from 2 p.m. onwards she sleeps...and that's when I take advantage of it, I lie in bed for a while... and I rest, I sleep that ..... At 5 pm we give her a snack, from 5 pm onwards we have to give her water, medication, we have to check if she has sugar, if she has a fever, that's how we take care of her, but ....... we have to be here .... if she has mucus, because she has mucus. She had the habit of swallowing it and lately she does not swallow it and it accumulates, of course, that is not good, from time to time it dries out, you cannot get it out and you have to put your finger with a gauze (I wet it with a liquid I have) and I take out all the dry stuff she has in there and ......that is the chore our ....

M - The task is yours as far as I can see.........6'50

E5- No, my daughters too. My eldest daughter helps me a little bit, she comes and gives her dinner. Tonight she's leaving and tomorrow she's already told me. Of course, seeing me alone, too... what do I do, call the SAMU, something else...

M - He can't do anything else and that's probably what he has to do.

E5- It has already happened to me, because there are nights when she has had a sugar level of 380, so I prick her and then I do it again and do the test again and if I see that it has gone down, then fine, I leave it there, we already know more or less what is going to happen, we have done it several times already.

SILENCE

M - How are you doing?

E5 - I am taking it because.....yo I am a person who has been encouraged and that's it, that's how it is and outside......pero, it's bad to take it eh, it's very bad 7'50

M - Because it is a sacrifice

E5- In fact, if I have to do something, it's impossible, I can't do it....

M - Sure, have you tried to ask for any kind of help? Or you can't.

E5 - No, because I have worked a lot in this life and I have been able to build a small patrimony, what happens? That now, as I have this small patrimony, I have no rights....

M - What have you worked for? 8'27

E5 - I have been a cabinetmaker.... in wood. I know elderly people who stay at home all day long, all day long, because they cannot. They have the minimum pension, eh

M - Do you get more or less than 1000€ of pension?

SILENCE

M - More than 1000 euros?

E5 - That's about it. Yes

M - Man, it is an important factor, the resources, that a person has.

E5 - My daughters have told me "if you can't we'll help you", but of course, I don't care, anyway, as long as I don't lack.... 9'26

M - If you can solve the issues for the time being, that is very important.

E5 - Of course, it is the most essential thing, otherwise you would have to be (taking care of your wife) all day long. Because you can't have a wife. Well, then, maybe you are entitled to assistance, but you don't know to what extent the assistance is of interest.

M - Well, aid for people who do not have any resources, well, it is good for them, because even if it is only two or three hours, well, for a person who does not have any kind of aid, it is good, but of course, as long as a person has a job, it is good for them. .....

E5 - Anything that comes, so that the person who is here can escape a little bit, just going to have a coffee at the bar, that's enough. At least she gets out of the house.

M - And at least you have that respite.

E5 - I had to learn to cook, to clean, to...of course ..... That was my job and she .....

M - She was in charge of the house....

E5 - She, she was at home. She has never worked. Of course, then you have to learn everything. She was brought here.... that's another one.... They brought her here from the hospital when the thrombosis happened to her, well, let's start from the beginning 10'53 From the day center they called me (I was taking her to a day center) they told me and said: "she is very ill and what if you come and take her to the hospital" I said; "well", I was ignorant of all this (today, I know), but then, I didn't know anything, I was, working and working....

M - Many times things happen that we don't know about....

E5 - I arrive and they tell me: "she is vomiting", well, very well, I'll go.... I take her to the hospital and from there, of course. They (at the day center) already knew what she had, I........ SILENCE

M - Do you think they knew what had happened to your wife?

E5 - I think they already knew what she had. So, I went there, I picked her up and I saw that it was not a normal treatment (at the day care center).... it was a treatment of this kind of...... more pity (I don't know if you understand me).

M - Yes, yes.

E5 - A little bit weird, well..... anyway, that's my assumptions. I brought her to the hospital, the hospital was full, full: "What's wrong with her?", well I don't know, she was vomiting, so and so. "So they took her nine hours later....Allí vomiting and I told them "please, do a blood glucose test, to see if it is that or what is wrong with her"...... It was already thrombosis, coming in the car to the hospital, she already did it, she already vomited...I think that if they knew (at the day center) they should have told me: "E5, we think we have a cerebral thrombosis, what do you think if we take an ambulance and take her to the hospital? I go to the hospital and that's it. When she arrives: "What do you have? A cerebral thrombosis. This doesn't mean that she was "cured", she had it there.....

M - Do you think that if the performance had been quicker? ....

E5 - It would have been something else. In any case, you are an ATS and you know that when it happens, it happens a lot or not at all......

M - Yes, yes

E5 - Eh, well, they took her nine hours later 13'14 Of course, after nine hours I realized: (ah, she was discharged) and I realized and said, "she had a thrombosis, you can't see her, with her tongue like that and everything, such......" they told me "no, no, you have to take her out and bring her back in" I said "no, no, you have discharged her and she is not ready for that". Of course, we were there for nine hours, tired of being there.... we took her and I brought her to me.......

M - And you came home?

E5 - It was already a threat, it was nothing else "you take her home and admit her again" No sir, she has a thrombosis, I admit her here, she has been discharged, but that's no good to me. Discussion, mess and mess, that we never win (that's clear), you can discuss that.... No, to the street....and they throw me to the street, eh. Well anyway, I took her up the stairs and it took me God and help to get her up, all by myself....she fell down of course, I couldn't pick her up anywhere.....so, I took her home, we put her to bed. Then, they were not capable...... when he took her to the hospital, the doctor, the neurologist, told me "she is very bad, she is terrible" Because he repeated eh. Well, he was at the hospital and he told me that we had to take her to XXX or at home. But of course, to take her home, they said "they will come from the intensive care unit such and such....". Four days later they came, four days later, they didn't even leave me a written paper with what I had to give her, yes, I had the discharge paper, a discharge report with the medication I had to give her, but not how I had to take care of her.......

M - Nobody told her anything? ....

E5 - Nothing, nothing, nothing. They left her there, in bed, what do we do?...my daughter there looking at me and .... What do we do? What do we feed her? Sure....

M - She didn't eat...

E5 - No nothing, we had to feed her, I started to make her very light soups and things, whatever I knew....bueno.... I put in food and things, meat, we were feeding her. After that, of course, after 16'29 we had to take her back to the hospital because of the wounds. The second time we admitted her, she came from intensive care, but we had to take her because she was very ill and had anemia and things, of course. That's when they gave her the .... these shakes that give....

M - Yes, something to feed her?

E5 - Envelopes of.... We just give her this (gets up to bring me the bottles of food supplements) and we feed, we put a little cheese, we give her an egg for a week....

M - Yes, these are foods that people who can't swallow take....

E5 - It is this, what we give him....

M - Yes, there are different brands....

E5 - So, this is very good eh.

M - They are supplements that are perfectly nourished people....

E5 - This is very good.... Apart from this, we give him a little cheese from "Burgos de Arias" every day, once a week we give him an egg, we beat it, from time to time we give him four or five macaroni, he mashes everything and we give it to him, well.....but now, we cannot give him all this, as he has a fever again....yo, I guess it is the sores, but............

M - Well, now you do have contact with the health center....

E5 - Yes but, the health center, it happens....

M - And is the doctor who sees her there...?

E5 - The doctor?

M - Is it the one at the hospital?

E5 - No, the family doctor, since we are here, let's say it all.... has come twice, because I have asked him, twice, in four years. Well, the cure, from there "What's wrong with E5?" Well, he has a fever, so what is it? "Well, it's the sores".....That's the doctor's cure...and the nurses..... I don't mean XXX or anybody else eh....

M - Anyway, I have to tell you to be calm, because neither XXX nor anyone else will listen to you...

E5 - They are saturated eh, they are like this (gestures with hand) saturated eh. This center has 59,000 people, this center alone, what happens? They can't go to everything......... SILENCE

M - Well, but you say that and I understand you....

E5 - Well, I say it this way or I see it this way. For Easter, Thursday, Friday, Saturday, Sunday and Monday, five days......... came here for one day to cure her. I imagine that from then on, those wounds are not taken care of. Yesterday, I said something to XXX, but I don't like to say anything to anyone, everyone knows what they have to do. But, I had to tell her, she heals her one day and the next day I cure her I.....but of course, what I told her: "the hand of a professional is not the same as the hand of an "amateur" like me, you, leave me the note there and I do it, but, it's not the same, it's not the same, it's very different XXX. So, I am going to cure her, but if it continues to be this bad, we will have to do something." ....

M - Does she have an anti-scalp mattress?

E5 - Yes, she has it, but now, either it has been punctured or it has broken down, because there is one of the parts that is not ....

M - That is not padded....

E5 - That is not filled and of course. XXX (XXX, she is the doctor), she feels so bad about prescribing, that I had to tell her one day: "XXX, I don't want anything you don't need, I don't want it. Why do I want a diaper, if I'm not going to use it, I don't want it, am I going to throw it away, no, I'm not one to throw away, no, but, whatever she needs, I want it.....".

M - It is what he needs.

E5 - What do we do with him? Eh, he seems to take care of me, but, no, not in principle.

M - Do you notice that you have to be the one who.......

E5 - Yes, yes, now, of course, I will have to go and tell him "XXX, the mattress is no longer good, it's bad".

M - It is fundamental, it is fundamental, it is one of the important things....

E5 - Because the compressor makes a lot of noise and half of the mattress is already empty.

M - There is a leak.

E5 - Or the return, something that does not work well....That is four years, 24 hours a day it is working.

M - These devices have a life...that's for sure 21'35

E5 - Well that and now, well yes, this woman is behaving very well, now she is washing and fixing it, because, between the sugar, the fever and everything, she is sweating non-stop, so you have to be on top of it with the cleaning, because if not....

M: And you haven't thought about her being admitted to XXX? I say XXX because it is a chronic center.

E5 - XXX, it's a hospital, very good...

M - It's a hospital for chronically ill patients, the problem is that it's far away...

E5 - It's very good, but, admitting her there would be....

M - Haven't you thought about it?

E5 - Yes, yes I have considered it, but, admitting her there, I know she wouldn't.... the "care" she has here, she wouldn't take it. Here, she has everything, the medication, she has, we have everything and if something happens, I have the doctor, the hospital.

M - Do you think that in a center like XXX she would not be as well cared for?

E5 - No

M - You don't think so

E5 - No

M - It is an important reason, for....

E5 - I don't think so....

M - To compensate for the burden you carry............

E5 - For us, it would be worse, because we would have to be there all day long. The 24 hours, they get used to it, if the relatives are not there, it seems that nothing gets done.... and of course, the 24 hours, who can stand it there? The trip, being there and Saturdays and Sundays, with my daughters.........

M - Seen this way, of course it doesn't make sense. You are calmer at home taking care of her than if you have to go, to come, thinking that....

E5 - Here, if I feel like taking a nap, I go to bed and that's it, if I feel like having a snack, I have a snack and that's it. If I feel like something, I do it...not there, I can't do anything there.

M: And you need to be comfortable and be fairly calm.

E5 - Yes, I already had problems at the hospital, because one of the times we admitted her, she started sweating and she was sweating and I know her, I know what it's all about, I said "please do a blood glucose test" They didn't want to...

M - Is it that you have had bad experiences E5, or not?

E5 - No, it is not that.... I had to take it, we are here discussing and it costs less to take it...I say "I am going to take it" because I had the little device, but, I, being in the hospital I do not take it, that is what the nurses are for. I had it, but I can't do the test and be busy there with them. Well, he says "no, no, we will do it" Well, tell me why "I don't have to give you explanations" well, fine, you don't do it, I will do it, but now, make sure you don't have any sugar....". And that, things like that, quite a lot.....Ah another one. When she had Alzheimer's, she was still walking around, she used to say "tiparraca" to everybody, it was a word she picked up and "tiparraca" even to the doctors.

M - Yes, yes

E5 - And...well "tiparraca" and "tiparraca". And one of the caregivers, the ones that clean them, wash them....

M - Assistants...

E5 - Yes, auxiliaries, I was in the hallway and I asked her what is this for? She says "this, for "tiparraca", like that.... I say: this bitch has no head and she is very sick and you have a head and you are not sick, so please respect her....

M - The truth is that she has had a bad time, the experiences have not been good....26'19

E5 - No, no, I've had a bad time in the hospital....

M - They have not been good experiences....

E5 - A question of doctors and that well.... I can't complain, but, sometimes... She was admitted in neurology and it was terrible there; but look where, one of the times, there were no beds in neurology and she was admitted in urology and there was a staff there, impeccable, impeccable: And it was right next door eh.

M: Fortunately...

E5 - Wonderfully. Here, another day, I told her "please do her a favor and take her blood pressure, she looks bad" and she said "And how do you know? I said "I know, because I know her, I have known her for a long time at home and I know what happens" .... They didn't take it; that was in neurology.

M: Nicolasa, does she communicate?

E5 - She doesn't speak, she doesn't swim....si wants to go to see her now. That's her (pointing to a photo) in 2001, when my daughter got married. That's when she started with Alzheimer's

M - It's a fast process and with the thrombosis problem....

E5 - She, here at home.... we had to hide the key, she would go to the street. One day I dropped it, it hit the table, she cut herself like this (points to the length)... a blood stream.... what do we do? If I call the SAMU when they come, he has bled to death, in short, he had those band-aids that serve as stitches, I made him a band-aid. I removed the hair and that, I fixed him well and .... first I put a gauze to stop the blood a little, I squeezed and then, I put the band-aids, I covered him and that .... disinfected and that's how he stayed. When the SAMU came, they said: "you did it impeccably, we are not going to touch it, it is fine, we will leave it like that" LAUGHTER....

M - Alzheimer's disease is very complicated.

E5 - Very bad. Here in front of me there is a woman who has a sister and she says to me: "E5, I am ....". I tell her: "Well, take it easy because that's how it is....".

M - What has your wife's illness meant to you?

E5 - A change from heaven to earth. Because today we have a little house in her town, and thank goodness my brother-in-law is there, otherwise it would have collapsed.....

M: Where are you from?

E5 - I am from XXX and she is from XXX. And that...it would be like going there, coming and living like two retired people, living at ease...., that's nothing, that's it, not being able to go out anywhere......

M - The truth is that we don't know the things that can happen in life....

E5 - Oh, I could imagine it in my life, in my life...........

M - If we could imagine it, I don't think we would be able to live......

E5 - Food, now 30'06 I'm already defending myself, I'm going....

M - Of course, he had to learn everything

E5 - Yes, yes, yes, yes, everything I know, I had to learn. I've already made the food, so.... If I want to go out, I have to do it like this. Because if I wait until eleven or twelve o'clock my morning is already gone. We finish with her at ten or ten-thirty, because we give her breakfast, medication, wash her a little bit and lift her into a wheelchair. We leave her here for a couple of hours or three. Even so.....then the lady puts her to bed and gives her food, we change her if she has made the bed; and my thing, well that...... the food, the washing machine LAUGHTER, a disaster....

M - A disaster because?

E5 - Because everything changes, from top to bottom and she is there, we have to take care of her too....es, apart from the chores of a house, this to take care of her....

M - Yes, yes

E5 - When she was...on Tuesday, on Wednesday it would be..... we changed her, we cured her, I cured her sacrum and that and just cured her..... she had diarrhea.... thank goodness my daughter was there, if not, I would die...We cleaned her, everything, sheets, everything, from top to bottom, everything, everything..... I put the washing machine to rinse her well and then with detergent and so on, she came out great....that's my........

M - You have to learn as things come up....

E5 - Then there is the...... going shopping, everything, everything. At the beginning, it was a problem, what do I buy? What do I do? Now, I am defending myself, I am learning. This interests me, this doesn't interest me, this is like this, this is not because....

M - Yes, yes.

E5 - I am learning....

M - This gives you a break.........

E5 - No, don't believe it, because I go shopping on Saturday. So, on Saturday I go to the market, I go to the market here in XXX. What happens, I arrive on Saturday, between preparing and arranging it (the shopping) I spend the morning, I run all morning. And there, of course, I get there and I'm not the first one, I have to wait. ....

M - And on Saturday, isn't the lady there?

E5 - No, on Saturday my daughter is there. She takes care of it, fixes a little, makes the food.... Saturday she takes that out of my way.

M - Man, you have to have a break...even if it means spending the whole morning at the market, it is a relief, it is what you were saying before, to go out and clear your head.

E5 - I have the habit of buying and freezing, I freeze everything and during the week, I take it out. The day before I think about what to do for tomorrow... so I take it out.

M - Like a perfect housewife

E5 - If not, I would be enslaved all day long, because if not, whatever food, you go and buy chicken, meat or whatever, one day and the next day I take it out. .............

M - Of course

E5 - So, I buy, freeze it and take it out, mine and my daughter's food. She is working and arrives at three-thirty almost at 3:30 ....

M - In the afternoon, are you alone? 34'38

E5 - In the afternoon, I am alone because she is not very well either....she has a disease that makes her lose muscle, so one day she goes to yoga, another day to the gym, another day.....in the afternoons of course and.....I can't say no, it's her health too................... SILENCE

M - It is not easy, do you have grandchildren?

E5 - Two, ah yes you told me before, a boy and a girl, there they are (points to the pictures).

M - Are they small?

E5 - Yes, (brings the photos) one is going to be six years old now in June and the other is 14 months old, now she has started walking....

M - They are beautiful...

E5 - Now they are all pretty, they are all handsome....

M - That too for....

E5 - This afternoon they will come, we are going to pick her up at school, at XXX....because my daughter the little one is a teacher at XXX, so she takes her there to class, but, on Fridays she has no class in the afternoons and we go to pick her up. It's a bundle of nerves........... LAUGHTER

M - But, that's also good.... when the children are lively, they give a lot of joy... LAUGHTER although grandfathers and grandmothers when they have to take care of the grandchildren say that "they are a people killer"....

E5 - They start jumping on the beds, he, with the age he is, I picked him up the other day and let him loose on the bed and he had some laughs......

M - Of course, the joy....

E5 - And he cut us all off, come on, me, everything....

M - A situation like your wife's must be..........SILENCE

E5 - Now, that's what I say, if she is well, then: "let's go shopping, well, let's go here or let's go there" and we take advantage of the retirement, that's why we have worked....

M - But so, besides, you are apparently very well....... how old are you?

E5 - I'm going to be 73, I do a lot of sports.

M - Have you always played sports?

E5 - Yes, yes almost always, I have left it a little bit but I have continued again, yes. I have not smoked, I have taken care of myself and that shows and Ella, too, apart from the fact that she was diabetic, she was very healthy, she was very well and the diabetes was very well controlled, she was fine, the same as now, she has everything impeccable, heart, liver, everything, impeccable...... well, before falling..... now she is a little "sickly" we do not know what will happen to her, I imagine that it will be the sores............

M - It is possible.

E5 - That's why she is holding up, if she is well cared for and has everything healthy, she is holding up with everything...taking care of her, giving her medication and everything, she is holding up with...everything.

M: Why do you take care of her? 38'36

E5 - You have to take care of her, she is there, I think she would have done it to me, right? .....

M - But, do you think that... "I do it because I think she would do it to me too" And why do you think this?

E5 - Well, I think .... when they told me to take her to XXX, I say: "no, in XXX, they'll kill her, no, I don't take her, I prefer to keep her at home. If something has to happen, it will happen in front of me" and she takes care of herself until it's her turn. We know what is there, eh, he is a sick person who has no cure whatsoever, but anyway, there he is. He doesn't enjoy it either. We don't know if we are doing good or bad, we don't know this. Sometimes I think about it, am I right to take care of her or am I wrong?

M - Do you think that the things you do to her are right or wrong, or why do you think that if you didn't take care of her, it would be over sooner and that's it? ...........

E5 - Of course, of course, thinking that if you didn't take care of her, she would be gone.....vale, but.......

M - There is death......

E5 - What would she prefer more, to leave or to stay?...She doesn't prefer anything, because she doesn't think..............

M - It's a dilemma........

E5 - But hey, we have to take care of her, she is there...well, I see it that way, she is there........SILENCE I think we have to take care of her, until God wants to take her away....

M - You think it's a duty, or an obligation, or out of love........ Responsibility

E5 - It is the three things, one that we have lived 50 years together, since we met, our whole life, it is our whole life...since we met, until today, 50 years have passed. It's a person, you say love...well, it exists or it doesn't exist...yes, you love her; then responsibility, duty, everything, everything is there. It's all, it's a whole, isn't it?

M - Yes, I think like you....

E5 - A person...if we met when we were 18, until today...look at the years that have passed....

M - What year did you get married?

E5 - We got married in '62 and we dated for 4, 5 or 6 years at least...all our life, yes

M - All my life and of course, when you need the other person...

E5 - That's why the nurse said to me "and how do you know?" when I told her about... How can I not know, eh? (thinks for the other person). She became diabetic when she had her second child, that's when she was declared diabetic SILENCE

M - Have you always been living here? Is the apartment yours?

E5 - Yes, yes.

M - To tell you the truth, it's complicated...

E5 - A lot...he who doesn't know, doesn't know what this is...I, I saw once in the newspaper "an old man, who killed his wife and then he killed himself, she had Alzheimer's" and I thought (all this before coming to me), this man is not right in the head, eh, because she is sick.........and today I say...LAUGHTER "........."

M - Can you understand?

E5 - Of course I can understand it, that man was fed up, he had no help or he couldn't afford it and he said "well, what do we do here, well nothing, both of us out and that's it, I'm done suffering and so is she", yes.

M - I understand it because nowadays many times (I am a teacher and I am in the university) and many times, they talk about gender violence, when an elderly man has killed his wife and I ........ when I talk to the students and the subject comes up, I tell them: "always, you have to wait before saying anything (labeling), wait, ask, look at what happened, how they lived, what they did. Because, that is not gender violence, that is .............

E5 - We have reached the end....

M - The limit has been reached... a person says, "What should I do, maybe, of all the things I can do, this is the best...maybe, this is the best...it is hard, it is not understood, very well, but...we have to find out.

E5 - When they tell me "this man killed the woman, so and so... I say, let's see, how was the woman? She was sick, but sick of what? Alzheimer's...it's very heavy, eh, very hard. At the beginning, it's something I didn't...Here, at home, I was sitting there...she would sit there and I would sit there, to keep an eye on her, because the moment I saw her close her eyes, she would get up and look for the keys, and if she didn't find them, she would come and pinch me...of course, these are things that make you nervous. Here, a lady who (was chubby) would come and pinch me...LAUGHTER, I said "tell XXX, don't think I'm giving them to you"....

M - How many years was your wife like that?

E5 - With Alzheimer's? Four years too. My daughter 44'51 couldn't rest, on Saturday afternoons when she goes to bed, she couldn't, she (his wife) went and opened the door, pinched her...she couldn't see anyone sleeping, impossible, looking for keys, stirring things. One day, I dropped her (because she had already had thrombosis, but she was walking), eh. Well, that's when she stumbled, she fell, I had to pick her up at three in the afternoon (when my daughter went to bed), pick her up and take her to a garden, she was walking around, until five o'clock, seeing what she was doing "don't go so far, come here", the same thing. It's a tragedy....

M - As they are not aware of it, it doesn't matter one thing or the other...

E5 - One day, my daughter says to me: let's go to Carrefour and buy. I say: look, Carrefour is very big and you can't go shopping with her in a wheelchair, because you have to carry the wheelchair and I have to carry the cart. Well... In the market, it is not the same, I manage, I have everything close by and if I need something I go to Mercadona, I know it, I go, I turn around and get everything... Well, no, let's go there, okay let's go... what happened, she had the habit of pinching and she saw someone and XXXXXXXX........... LAUGHTER I would say, excuse me, but it's not right and so on...of course, people would shut up, they would see her, but, if there is someone, some evil shadow who doesn't understand "hey, don't take her out of the house" or something like that, a bad answer, are we going to argue, eh? Are we going to argue? No, you leave her at home, there in the wheelchair, let her rest and I go to the market, I buy and come back. We could not, we could not....

M - Besides, it's a stressful situation...

E5 - It makes you nervous, you arrive here nervous, arguing with her and with my daughter, because of her....

M - It is not only what it has meant for you... but for the family relationship....

A - The family relationship is ...... very tense. I am not saying it is bad, but tense, because my daughter, the eldest, she is single, what happens? That this XXXXX would also like to have fun in a different way, to live her life in a different way, what happens? That many days she is aggressive, she is bad, that she falls ill... I pay for it, that there is something that does not work..... is my fault, well, my fault, I am the one to blame. So, what's wrong? Me, as I am...

M - Pressing...

E5 - Pressuring, well, I get angry, and when I get angry, we argue.........and that's it. Then, of course, I'm the one who has a bad temper, but of course, boom, boom and boom....... And this is the atmosphere of a house where there is a sick person like this. Not only mine...because the one in front of me says "I am" because XXX is like this...... He gets out of bed and goes out naked and she (his wife before) also did it and goes to the toilet and goes out naked.....

M - It's a terrible problem...

E5 - Of course, if there was someone, he went out, he had to be very "cuidao", to be with her. Showering her, it was impossible...she would fuck.... I put a handle to (with heels that big), so she would pull them out...nothing, she didn't want to, she would scream....RISAS...the neighbors...

M - Your relationship with the neighborhood, well?

E5 - Yes, 49'11 What are they doing to him, don't worry, I'm showering him, that's how it is...

SILENCE

M - And how do you see the future E5?

E5 - I don't see anything......

M - Don't you think about the future?

E5 - I don't think, because how can I think when she is like this? I, I see my future if she were to go away.....(that is, if I don't go away first) eh, for the moment I think I'm going on vacation, I'm going alone to a place.....I want to be alone, maybe after four days I get tired...... but, I want to be alone, I'm going somewhere, to a house, wherever, but, alone. That way.........and then, I don't know, I don't know what I will do. Well, I'll go to the village, I'll come and go, as the house is there alone, I'll go there to spend a few days. I really like cycling, in fact I have always done a lot of cycling, so I would take the bike and there....

M - Do you do sports now or not much?

E5 - Yes, yes I do, I go out, the thing is that I go out at 10:30 am, that's when I can go out, now, around this time and...it's hot....

M - Well, it's just that now...

E5 - But I can't leave earlier, because if I could leave today at 7 a.m., I would be here at 10 a.m., but it's the opposite, I leave at 10:30 a.m. and I arrive at 1:30 p.m., of course.

M - And you can't leave her alone?

E5 - No, not alone. I go out because this lady stays with me, otherwise, when I have a day or so, I go out for a little while.

M Of course, even if you leave her alone it would be more stressful for you. ....

E5 - No, it's impossible. Because she can drown, so she can drown.

SILENCE

M - She has to adapt, as she is doing....

E5 - Of course, I have to adapt to her rules, first her and then me.

M - And do you think she won't last long?

E5 - I don't know, the doctor gave us two and a half months...that was four years ago. Sometimes she tells me "E5, how she deceived us", well yes... I do not reproach her, eh. She came out of the thrombosis and from then on I accept it and that's it.

M - A strong and well-cared woman...

E5 - Of course. I accept the disease and there it is, we are taking care of it, taking care of it and there it is...

SILENCE

E5 - She has a (we were in town on Monday), because, it's another story.........My sister-in-law fell ill, she was bleeding inside and so on, she seems to be doing well, but, she has been in the hospital for a month, without eating and without anything and she has been very bad. I have not been able to go for not telling my daughters "come, you stay one day", I take the train on Sunday, I go quietly with the train (I do not go with the car), and I get there. The hospital station is a bit far, but well, I take a cab and it takes me there and that's it. I see it and then I take my train and I come home so calm, I have the whole day. Besides, this way I get distracted, I look out the window and I get distracted...... There was no way, one, because she has the children and the other one has such.... And on Monday, I told her: "Monday, to the village" that this lady could stay and so on. And we left, it was going and coming back 250 km, in one day, well, and we saw my sister-in-law because she has already been discharged, she is at home and so on. The logical thing to do is to see her even if it is just to comply, but I have to go....Total, that was on Monday, eh, and anyway that's it, we have already complied with that, I already have another problem outside......

M - Many times we have obligations that are put on us or that we put on ourselves....

E5 - No, it is an obligation, because if she is sick I think it is an obligation to go see her.

M - Other times I also like it, I like to go to see how she is doing.

E5 - Yes, I already said "XXX, on Monday, let's go to see you in town" " uy que detalle" .... I say "no, it's not a detail, I'm going because I want to see you, it's not a detail, otherwise I wouldn't go, I already have a good excuse to tell you that I'm not going..."

M - Well, yes. The situations are painful, it's very difficult and... Do you think you are alone? Do you feel alone?

E5 - No, no, I don't feel alone either, well, SILENCE, neither good nor bad, I'm here, I have an obligation and that's it.....not alone. There are my daughters, because, although they can't do anything and they don't do it because they trust me, but, if something happens, they are there............

M - That also happens many times......

E5 - If a problem occurred, I would say "XXX, look what....." but I have to ask, and I am not a person to ask....

M - You also have to learn how to ask....

E5 - No, I don't like to ask....

M - Because you suffer, when you don't ask you suffer and many times, sons (I don't have parents anymore, they passed away), but, many times, daughters think that parents can and that they are fine and that they are doing that because they can....and maybe it turns out that, between them not asking and us not being able to put ourselves in their shoes.... so we don't have a good connection.

E5 - My eldest daughter has very little dialogue, she is not talkative, she is very reserved, so reserved that sometimes I get angry with her, too much. She, for example, will never ask me or tell me "did you give mommy some water"... she fills the bottle and if she sees that there is half of it, I gave it to her... she is like that, but she doesn't say "did you give her water, so and so", she is very reserved.

M - Of course, and at times when maybe we all need each other for certain things, we don't realize the situations...

E5 - She knows the obligation, she comes to give her dinner, she is also obliged, eh, she is obliged....

M - Yes, yes, yes, the whole family...

E5 - We have to clean her, because she cleans her, every time we change her place, well, we look at her to see if she has done belly or not......

M - it is a complicated situation for all the members of the family, it is like that....

The doorbell rings and it is the nurse who comes to cure XXX. Recording 4(2) is of no interest for the purpose of the interview, she tells about her life as a cyclist.

**E6**

M - What I am mainly interested in is how long have you been taking care of your mother? How long have you been taking care of her?

E6 - My mother was quite well, but she came here when my brother left. She lived alone and my brother has been gone for 9 years and since then she came to live here with me. She is sick taking care of her, so .... what will I be? four or five years, I don't know, and... I am the one who takes care of her, I feed her, I wash her, everything...

M - Do you need help to put her to bed, dress her?

E6 - To pick her up. My husband, because I can't. His legs are useless, he had hip surgery and his legs are useless. My husband carries her with the "tacatá" on one arm and I carry her on the other arm and she can't do it with the "tacatá", and that's the way it is... I take care of her all day long. There comes a time when you can't take it anymore, because when the person can't take care of herself, you have to make all the effort and that's a dead weight that no one can bear. If my husband is not there, I can't lift her; the doctors have already told me not to make any effort, because I have had a thrombosis in my eye, because of the effort, and that's the way we are going.

SILENCE.

M - How old is your mother?

E6 - The same day I am 71, she is 91 years old. 2'16

M - The same day they were born.

E6 - Yes

M - She is 91 years old

E6 - In August 91

M - Can they communicate?

E6 - Yes, yes, sometimes yes and sometimes no..... There are times when her grandchildren come and she says to me: "Who are these who have come?"-"Girl, well, they are your grandchildren" - She thinks back and remembers. Some days she knows them and some days she doesn't. There are days, I wonder if it's the blood flow, isn't it?

M - Well, it's possible, there are lapses due to lack of blood flow ........

E6 - Of course. That is happening a lot now

M - Yes, yes, of course

E6 - Now I tell him "we are going to the beach" - like every year - he doesn't remember, he says "to the beach", he doesn't remember.

M - He goes with you, of course

E6 - Oops. I believe it, I believe it. I don't have a summer, because it's the same as if I were at home....... cooler, but that's all.

M: Where are you going?

E6 - To XXX, it's close by.

M - Sure, but for you, it's a change of environment....

E6 - Well, yes, I think I'm going, I don't know.... to change the environment is something else. And at the beach, my husband stays with my mother, so I go to the, I go to the beach for a little while....and we take turns 3'48

M - They take turns, of course.

E6 - And so we go....

M - How has your life changed since you have been taking care of your mother?

E6 - Well, it has changed a lot, because I have friends. We used to go out a lot, these IMSERSO trips, well, every year I went on two or three little trips and ......... you can see how well it goes......RISAS. That is over.

M: And you don't have anyone to help you?

E6 - Yes, paying....RISAS

M - Some relative....

E6 - I have a sister-in-law, a brother I had died and it's not the same anymore. If I had my brother, well, I would tell him: "I leave my mother here" .......

M - Could you share? You mean.

E6 - Sure, or I could take a trip and tell my brother: "I'm leaving", but not now. Now I don't have anyone, just me and my husband. ......

M - Do you have children?

E6 - Only one daughter and she hasn't had any children either, and I say: "daughter, what a short family", it's a shame not to have grandchildren too, because I love little children, it seems that they bring joy and so, well, look, taking care of the mother....

M: Do you go out? Have you lost your friendships?

E6 - On Sundays, come.... no, no, I don't want to lose them. On Sundays a girl comes at 12 o'clock and leaves at 5:30, every Sunday 5:21 and I go out with my friends to eat. I have no other distraction. The four or five hours that this girl comes, I meet them where they are going to eat and we go to eat with them and that's it. .........

M: Are you going with your husband?

E6 With my husband and friends.... Now that I see my mother better and more "lively", I take her out. Last Sunday I took her to my brother's house, to my nephews' house.....we spent the whole Sunday there, in the house. She was sitting there on the sofa watching TV, well, not watching TV either, because she doesn't see. She sees a little bit out of the corner of her eye, she has macular degeneration and can't see. Well, she had a good time. He has seven grandchildren and of course, the kids are always around and seeing so many of them, my nephews, my sister-in-law, he had a great time on Sunday. And the Sunday before, we went to spend the day at XXX and we also took her.......es that I, five hours, I do not enjoy almost Sunday. It goes by very quickly, I say "I'd rather take her with me and spend the whole day until the sun goes down, than come at 5:30". I have already done it two Sundays, the Sunday of the chalet and the Sunday I took her to the XXX. You leave in the morning at 11 or 12 o'clock and come at 8 or 9 o'clock and you have the whole Sunday, even if she is there. It is that the other way you do not enjoy anything, five hours if she arrives and that's it. Waiting for Sunday to come all week long 7'02

M: Who drives them?

E6'- My husband, he drives ......

M - How old is your husband?

E6 - 78, but you see him and he doesn't look it.

M - Neither do you, eh

E6 - Now I'll tell him to come out and see............

M - You don't look how old you are either.

E6 - I do, I see that I look it, but he, he is young....

M - If he still drives, they can go out with the car and that's a lot of autonomy.

E6 - That's everything... we put my mother in front, next to my husband, because that way she can stretch her legs, and that's wonderful. Then, the wheelchair, he has it there, you take it, she grabs his neck and he does the strength and then, to the wheelchair, and from there, to the apartment, what can we do now? There we go for a walk in the afternoons, I go there and it's something else. Here we also have a good river, but you change the environment... LAUGHTER

M - It is very important ........

E6 - And my back... from here (points to the cervicals) a pain, the eye "cracked", putting on the XXXXXXXX, but bad, bad eh.

M: And what has all this meant?

E6 - Well, it could be because of the efforts or because... but I think it could be because of the efforts. Besides, a cousin of mine says to me: "wait, when the years go by, you will have to put on the girdle and all day long you will not take it off because of the pain that stays in your back", 8'15 because that happened to her with her father and she said to me: "it is very heavy and then everything comes out", it is coming out, it is coming out, because right now I have a pain behind here, I have taken a thermalgin and there it is, without leaving, without the pain going away. I don't know, but, I'm not worth a penny.......

M - The burden you are carrying is not just any old thing.......

E6 You know what happens, you have to have it because even if a woman comes once a week....

M - Do you have domestic help?

E6 - Only one day and then the whole week.....then you have to take care of everything.......very heavy.

M - Does she eat everything?

E6 - She eats only purees. Today, the cleaning lady was there and I said "oh my God, I'm late and I don't know what I'm going to give my mother for lunch" and I said yes, I had boiled cauliflower for us, I put two potatoes and....

Husband appears 9'58 and a greeting conversation takes place ...... 11'44

E6 - We are talking about everything we do with the mother, which is very heavy, you have to do all the strength, lift her up and that is very heavy.

HUSBAND - It is that, they say to bring a lady, but I say "if I am here, why do we need a lady" if she is at home, it is another story, do you understand?

M - Man, I couldn't be alone ......

E6 - All day long, a lady......

HUSBAND - But, anyway, we're holding on and she behaves very well...

E6 - Between all of us... we'll get by.

HUSBAND - And then, Carmen has had the stumble she's had and so we go on.

E6 - The thrombosis 12'26

The husband sits on a sofa. I continue...

M: Carmen, have you always devoted yourself to the family? Or have you worked outside the home?

E6 - 25 years working in a grocery store, 25 years.

M - Have you worked outside the home

E6 - No, no, I used to own the grocery stores, I was there all day long. Before, it was all day long. In the store we had two sales assistants, because before there were no supermarkets and the grocery stores sold whatever you wanted. We took that time when there were no supermarkets and we had two girls and we couldn't keep up with what we sold, a lot, a lot. And my daughter went to school at XXX. I have been working there for 25 years, as a black woman.

M: Do you both receive a pension?

E6 - No, my husband didn't want to, he says: "why do you have to pay anything" 13'45 he didn't want to, and now he feels bad about it, and I feel even worse, because now I would receive

M - They only charge for his.

E6 - Of course, only his and look, mine has not been worth anything. ....

M - Before these things were not thought of....

E6 - Of course they were not thought of and that was a big mistake, because thank God we are doing very well, but, now that little money would come in handy....

M - Man, we don't know what can happen to us........... 14'31 How are you living with your mother?

E6 - Well, I... I'm not living it badly at all. I am very close to my mother. Because all my life... my daughter says: "you don't live, daughter", I, it's passion that I have for my mother, it's one thing. I love my husband and my daughter, but I love my mother... It has been my whole life. I got married, I had the grocery store, I had my daughter, I had my apartment... and my mother said "as you have to go with the little girl now, or at night or with the cold", we already had the room there to stay. And I have been all my life with my mother, I have not had that with my husband. I have had, of course, what one must have, but I have been very devoted to my mother and now it is that, that I love her so much, well, I take it quite well, I think and say "I love her so much" I would never want her to leave me, it is that, what I also think and say "I'm missing 15'34 trips with my friends. We are six or seven couples and we had such a good time and I am missing all that, of course, all my youth, not all my youth, this age, so good that you are not completely old, that you can be worth it, it is now when you can enjoy yourself, it is now and I am missing that. For four or five years I have had nothing else to do but to take care of my mother.... I have nothing else 16'04

M: Why do you take care of her? Why would you say you take care of her?

E6 I...because I love her, I love her very much.

M - Because you love her.

E6 - It's an exaggerated thing, it's true. The other daughters will be...but my mother deserves it, they are people who are loved because there are older people who are unfriendly and "nagging" as I say, but not my mother, my mother is very sweet, if she treats her she is sweet, she has a sweetness about her that you have to love her very much...that's my mother, and of course, I take care of her as much as I can and more.

M - Have you taken care of other people?

E6 - Not me

M - Apart from taking care of her daughter.......

E6 – Nothing

M - To his father either....

E6 - My father, that already.....he also me 16'57 some nights, because he had a disease.....now I don't remember, of the bones...he started to get old, old and at night we stayed, one son, another night, my mother and I have been with my father, but not so much....

M - Was he in the hospital?

E6 - He was in the hospital for a few days, I don't know if eight or ten days, he came home and then we admitted him again and so he made two or three trips to the hospital, going back and forth. He died at home, I was alone when he left. It was something else, of course, he could manage, with a "gayato", he went around, around the house, but my mother could not. My mother can't manage, my mother can't walk, nothing, nothing, nothing, nothing, if you don't help her, nothing, and it's very hard, you have to be with her, you can't leave her.

SILENCE

M - Do you rest at night?

E6 - The only good thing she has, she doesn't open her mouth all night, thank God.

M - She rests and you ....

E6 - Yes, if the night was busy I wouldn't be able to stand it, because sleeping is everything for me. When you sleep, you rest and she doesn't even say "oh, my poor thing," all night long, and maybe she is awake. There are times when she talks to herself and I am listening to her and I am going to see what she says "I will walk". Perquè nosaltres parlem en valencià, you understand it, don't you?

M - Perfectly.... 18'44

E6 - "És que no puc caminar" says "si no em porten, jo no puc caminar". Before, with the "tacatá", she could, but now, not even with the "tacatá"...she has some conversations on her own....y look, she doesn't call me or anything and she keeps hitting her head and talking whatever comes to her mind, and look at.....Pero resting at night, all night long, how good that is. Sometimes, he has gone out of his mind and said some things, he spent the night saying that he had a room full of people and they stuck their tongues out at him and...he was living it as if it were real, and he told me "xica, dis-los que se'n vagen ! Look, they are right in front of me" and she was telling it as if she was living it, other times "chiqueta agafa a la chiqueta que es va a caure, agafa-la" Then "que tenia la casa llena de lladres que li estaven robant". Oh, some things have happened......... and now thank God............

M - That situation has not happened again.

E6 - With the medication he has been taking, since then he has not missed a day, he is very well. He doesn't say anything all night long. But he has spent some bad, very bad nights. The window, she still remembers and there is no night that she doesn't ask me "has tancat la finestra" every night of the year, but "mare, no li he dit que si, que l'he tancat" she repeats again "la finestra esta tancada" siiii, esta cerrada.... 20'36. It is the fear she has, it is a horrible fear and when her friends are down here and I see her so sleepy and I say: "I'm going down....", sleepy?....

She is very suspicious of me getting down. "Carmensin, Carmensin"......RISAS...........siempre gritando. One day, last year we were having dinner here under all our friends, I had left her sleeping. She got up, went to the terrace (she could walk then, this was before she broke her hip), and "XXX, look you've got a house full of thieves, you're stealing everything" and one of my friends said: "Uy, there, a lady is shouting, I think it's your mother", I hear that, I turn up and say "uy, it's my mother" and I run up and tell her "però tu que fas mare" and she says "tens la casa plena de lladres i t'ho estan robant tot".

M - Of course, she didn't realize it, she was disoriented............

E6 - They all found out that my mother was, well look, her head was not working. And now, she seems to be calm, that doesn't happen anymore, we'll see. When she goes she is not as calm as she is here, but it is because I...........

M - You will notice a different atmosphere....

E6 - The atmosphere, that I am suspicious to go downstairs, of course, it is normal, all day long she is locked up there and I know that they are downstairs and....RISAS...I am suspicious, LAUGHING and she is always with her ear.......... "on, on esta XXX, on esta la meua filla" 22'19 to XXX to my husband, she is always asking.

M - Do you have help there?

E6 - No either, but I have told this girl to come one day a week, because I am .... and I tell you, from here, from the neck, today I'm worn out, and one day a week, she cleans me better and then it's just mopping and dusting, that's it, there are three of us, there are no kids .... who dirty are the kids. They have never come there, but this year I can't, I am in a lot of pain, and today I have already told the girl "look, until next Monday we will be there" and that's how we have arranged it.

M - Very well

E6 - All this is what happens here in this house.

M - But she is doing well.

E6 - I'm doing well ....? And now, I'll tell you how I'm doing (with irony) having fun......

M - It seems from what you say, that you're doing fine....

E6 - Sure, (it seems that the husband's presence intimidates you) with my pains... What am I going to do? I have no choice.... and the eye, which I have had a lot..... I say, my goodness, with this eye I can't see, just like with this other one, it happens to me with this one How could I have had it? I thought I was going to go blind.......

M - No wonder.

E6 - I was desperate, I couldn't handle my soul (teary eyes) and this was because of the insurance. They said, "if you go to a private one, it seems to be better 23'55 you should have gone to a private one". Oh, I trusted the insurance, as they say that good doctors are also in the insurance and the one who was going to operate on me was a professor, I don't know what from the hospital, he was the boss and I, well ...... but look, they have left me without sight and as I have been resentful of this, what happened to me, my husband said, well, let's go to a mutual insurance company that I have private and you put yourself in his hands. And I am with a private one and we went immediately, they did the tests, that you urinate yellow and everything, some very strange tests that in this one (he points to the other eye), they did not do all those tests at all and after all they put laser, some lights that they give you there and after the results, they told me that everything was very good, but that now I have to be always in revision so as not to leave that eye ever. Of course, if I don't have any sight in this one. ....

M - The other one has to be taken care of....

E6 - That's why they don't want me to make efforts, I have to take care of this eye, I have to take my blood pressure every week, they have given me a sheet with an eye and every week I have to look through it.... if the squares are always fine, I will be fine in that eye, but if those squares dance, immediately, I should call him. A lot of things so that we can maintain that eye.

M - It is an important concern.

E6 - Very important so that's what happens to me 25'54 which is enough.

M - Sight, it's so important....

E6 - If you are missing an arm or a leg, how bad it is....but, the sight, there is the sight.....you close your eyes and you don't see anything, that's horrible. I see a blind person and.... it makes me sad, because, oh if I could see myself like that, now I look more closely.

M - Of course, as he is now in this situation, going through this process....

E6 - Yes, I pay attention, I pay attention, now, it's true.

M: And how do you manage to cook and all that?

E6 - I, with this eye I see perfectly, I see very well, very well. I paint myself and everything.....RISAS....because I like to paint myself and I see very well, yes, with one eye. But I didn't realize that I had lost this eye, what a strange thing, eh?

M: How is it possible E6?

E6 - Well, nothing, I went, because I saw cobwebs. First it started like that, before I lost my eye. I went to the insurance, to XXXX, which is where I go and I told her: "hey, I see like cobwebs" she started to look at me, but you know, I didn't like her at all, nothing, she was a bit stunned, I don't know, like the one I have now, I see her as a calmer lady and more interested in me (we have to go and tell her everything that has happened to me) (she says to her husband) because of the interest she showed in me..... and not that one, the opposite, she says "But you don't have cataracts or anything" I say "Oh, I don't have cataracts, but I have something", she says... "Well, come back in 15 or 20 days" The thing is that when I came back, she goes and says "now, look at those letters" she covers my eye. I said, "tell me what the letters are" oh, I can't see anything, and she said, "you can't see anything" I said, nothing, nothing, absolutely nothing "you can't see anything" I said "no, no" and she couldn't believe it, she said "immediately you go to the emergency room" she went with my daughter "or you go to XXX, but immediately" and my daughter and I went to XXXX. I arrived, they took fluid out of my eye, tests, things "you have to go to XXXX hospital, that is where you belong" the next day to XXXX, there, doctors and more doctors and in the end ..... you have to have an operation, they operated on me and they believed that by operating in 8 days I would see ..... And I never saw again.

M - She has not recovered vision

E6 - They said "well, we are going to operate again" again, two operations, nothing, nothing, nothing.....And I have not seen any more. They say that the retina, there was I don't know what.... I don't know...what....

M - Detachment....

E6 - Retinal detachment. There were more complications, I lost my eye and I have always said that it was my fault...it was here. When I went there and said that I could see things, then she should have shown interest and told me "well, let's see, let's see what this is all about", but, go and come back after 20 days. In those days, that's when everything that happened to my eye happened to me. When they threw away my hand, there was nothing more to do, 29'49 and so on, that's how it went.

M: My God, E6 .......... Grandchildren, he told me that they don't have any.

E6 - No, another misfortune

M: Well, for the moment

E6 - No, no, my daughter is 48 years old, on Saturday she made them, I always say she is 49 (her husband looks at her) oh yes she is, my husband is there, it's true....

M - She is already 49 years old

E6 - And I always say 48

M - That's a year less

E6 - Look at 49 years old, they want to take out, but they say they are older, to take out a child.

M - Adopt?

E6 - If they want to, they have to go to XXX or XXX, abroad. Here, there are a lot of things to adopt, a lot of obstacles.

**E7**

The interview starts 0'25

M - How old is José?

E7 - 84 years old and she (his wife who is presenting, WOMAN) is 82 years old.

WOMAN - That's a lot of years, .......

E7 - I'm fine as far as I can be, even though I've had heart surgery, but she's very ill. Apart from the fact that her bones are very bad (for many years), she has had surgery on both knees, her hips, her womb and now she can't hear, you have to shout at her and even so, she doesn't understand much. But anyway, you have to put up with what comes............

M - Well, that's what this is about, how long have you been taking care of women?

E7 - Well, taking care practically, I don't know... since the first operation, about 14 or 15 years and she has a bad spine for more than 50 years and she has always been told that she was going to have a bad time with her spine, that she doesn't have an operation and so on... and she is very upset. We had a little house in Liria and we had to sell it because she did not come many weekends and I did not come alone either, where I was going to go...I was going to water, but anyway. As she has a lot of things, well... I have always liked cooking, she doesn't like me to go, and she tells me "nothing, go on, go away". ....

M - But does she cook?

E7- She doesn't like some things anymore...the fire, she thinks she has put it out, but she hasn't. This morning, she had some broth and we were in the kitchen. This morning, she had some broth and we were there and she told me "I don't know if I turned it off or not", it was in the fridge, very bad, very bad, very bad 2'27 She asks many times about her parents, about her grandfather "where is grandfather", what grandfather, the only grandfather is me.....RISAS

M - Does she know you?

E7 - Yes, yes, she knows almost everyone, man if she is a person who is not seen....but yes, she knows, she knows. She has times when she looks good, but other times she doesn't. She has a 17 year old granddaughter. He has a 17 year old granddaughter, ¡¡¡¡. He will name her at the end of the day !!!!!!!. 200 times "the girl is up" because no, today she doesn't go to school or she left two hours ago. She arrives at three o'clock and at twelve o'clock it starts "what time is it and she hasn't come yet"...but if they come at three....

M - He gets disoriented

E7 - Yes

M - Does your granddaughter live with you? 3'29

E7 - Yes, she and her parents have been here. They live here too, we are, them and us, we all live here, although they have the apartment, they go to sleep at their house (the daughter with her family has an apartment on the same landing) but we're here

M - You, you have help...........

E7 - Help is that.....We are all "touched". My daughter, with infantile paralysis at the age of four months, did not do well and then... what happens to young people. I used to work in one of these kidney centers, she had an affair with another guy, she wasn't well either. She has had two operations on her leg, her hand... And he is also a bit of a "psychic.

M - Yes, some mental illness...

E7 - XXX, both of them and they are bad, but we manage 4'28

M - We could say that you are the one who takes care of woman

E7 - We could say no, it's me who takes care of everyone.

M - Well and the house or have domestic help ....

E7 - Help, no, nothing .... my daughter when she is here, she scrubs the floor once a week, makes the beds, but her chores, my wife's and mine nothing, well I do.

M - What chores do you do, and your wife? 5'06

E7 - I give her a shower, dress some clothes, but.... her memory, she has nothing, nothing.

M - Is she diagnosed with dementia or hasn't she been assessed?

E7 - They said that she has the...that thing that is mentioned so much now, the....

M - Alzheimer's?

E7 - That, Alzheimer's, we went to the center of...the dementia specialist....

M - A service they have in the neighborhood?

E7 - No, it's on XXX Street .......

M - Do you go every day?

E7 - No, we went..........We have been to the psychiatrist, the psychologist and the family doctor says: "they have to see her" and we have an appointment for September 22...

M - A check-up?

E7 - Yes, it is convenient, they say she has Alzheimer's 6'44 There are days when she sees her and...Then, she gets very nervous, she is, she is very upset. In spite of having surgery, my head is fine, my leg sometimes...LAUGHTER

M - What did you do when you were young?

E7 - First in the fields, I worked in the coal, I went around to mow the fields, to the grape harvest, everything, and since I had an aunt here (my mother's sister), we are from XXX, do you know where she is?

M - Yes, man XXX.

E7 - We are from XXX, well, the first time I came down here, I fell in love with XXX, and after that... I did my military service (I also had bad luck) because XXX and the doctor told me "what do you do for a living?" in the fields "well, you can't work, because your lung is not visible" when they saw the x-rays, the heart and the lung were not visible. He gave me treatment and I did everything he told me to do because I had the illusion of getting well (young then). So, I came back and the doctor told me, "if it were not because I could see it I would not believe it". The change I had made "you can see what is there, before you could not see it, now you can do something, but do not work too much and remember that you have been very bad", those words have not been forgotten. So, little by little I started to work again in the field. But I had the habit of coming here. I told my aunt, as soon as there is some work, I'll get off and nothing, I started working in the XXX soda factory. Then I went with a truck inside XXX 9'24 good, I went through the towns, here to XXX, to XXX, to XXX. I had one of my own motorcycles. Not one, but three, and that's how I spent my life. Then, when I turned fifty-something, I said, this is about getting into a company, to retire... I did that, but the boss called me and said "XXX, look, I thought I'm going to close, keep the truck and what I'm going to give you at ........" I said "look, no because I was driving the truck but it was old" he said "give it a squeeze ...." I said "I'm sorry, but no...". I bought the motorcycle truck, I started working and I did quite well. When I turned fifty I said "out with everything" and went into XXX in a sanitation factory and I've retired there.

M - What a brave XXX because to start looking for a job was so different from what I had done when I was fifty-something years old. ....

E7 - When I came here, I was declared totally useless, first temporarily, I passed the medical tribunal and after three or four years, I was no longer renewed. They called me to go for a check-up. So, I didn't even pass..... and that's how we started the march, until today.

M: Do you receive a pension?

E7 - Yes

M: Do you receive less than 1,000 euros or more?

E7 - Less, not to lie, I get 703 euros.

M - And that is the pension, she has no other income?

E7 - She has a non-contributory pension, she receives 285€ and that is our story 11'49

The daughter arrives and joins the conversation. I continue I DON'T KNOW IF IT'S RIGHT.... E7 explains that he has liked cooking since he was a child. 13'77

E7 - In the post-war period my brother, my father and I would go and I would put on a pot with potatoes and salt and I liked to cook. Now, I don't trust her, especially with the fire. This morning she says "look at the fire", she wasn't on .........

M - You have to keep an eye on it

E7 - And so is the marcheta....

M - Has your life changed since your wife has been like this?

E7 - Man, it has changed, because I don't go out of the house, (looks at his daughter), man, I go out, but, very little, very little and I liked to go out and walk around, but now, for fear that she might fall. She fell in the corner and what happens; she hasn't fallen hard, not hard at all, but falling on her butt in the bathroom, in the little room we have there, in the kitchen... well....

M - What you notice most is that you go out much less.

E7 - In the morning, my daughter comes in and I go shopping ........

M - You take the opportunity to go out and get distracted.

E7 - Distract myself, I would like to go to the doctor's office 15'40

Woman intervenes... 15'58

E7 - The other day when she came, this one, the colleague, XXX, asked us some questions...

Intervention of the daughter 16'04

E7 - She told us "in three days she will be ready" 16'15

M - I would give them an assessment for the dependency law, to see if they can ask for any benefit.

E7 - There is one that is signed and it is full (the daughter takes out all the documents)

M - Well, if something is missing...

E7 - And this is the one that has not been signed.

M - It is necessary to try these things, in case there is some help.

The daughter intervenes 16'47. They all talk about the documents...

DAUGHTER - As I am handicapped... 17'18

E7 - I can't do it on my own because I can't make any effort because of the heart attack, I mean because of the heart attack... that is necessary. I try to get her to move because she likes to sit a lot, although the doctor told her that the less she sits, the better....

M - Let her walk and move around 17'47

E7 - Let her walk and change seats, just by moving she gets exercise. Of course, when I was operated on in San José three years ago, I came out of there looking as if I had been in the hospital. ....

M - It is a very important operation

E7 - I weighed 95 kilos and when I arrived I weighed 70.

They all speak at the same time 18'35

E7 - He looked like a dead man 18'54

M - What did they operate on him here at XXX Hospital? 19

E7 - Yes, but from the first day I left the hospital.

DAUGHTER - And a year after my father was operated on, they operated on my mother....

E7 - The first visit I went, she discharged me and told me "if there is anything to the emergency hospital" 19'13 that is the best of all, to the emergency room" and I didn't go there. One day, it seems that I noticed, of course, any little thing you have, you always think the worst. So I went with my nephew who sells bread, I told him "I'm going to go just in case" and he took me to the hospital. I called my daughter, she came, we left. I had some tests done....of the esophagus.

M - Yes, something in the esophagus, stomach....

E7 - Yes, he says the air, it pulls up, but there's nothing... 20'11

It is very complicated to follow the conversation

E7 - Oh, life....

M - You have to see how life changes 20'38

E7 - I was very comfortable in the village because I like the countryside very much........(Tells about her life) I have done everything 21'09 and I have not been afraid to do anything, that is to say that.....

M: Do you still have friends?

E7 - Yes, well, friends, especially some of them have already died 21'25. Of my fifth that we were 22 23'39 So nothing more and realize that one is old and you have to go through there. The bad thing is that she doesn't get old, she's already old "and I would do this, and I'll do this, like this, like this" 24'07

M - What feelings does she have?

E7 - I've realized that I have to go through that.

M - And why?

E7 - Oh, because we are old and because we are old we have to go through and everyone who sees me says "José, hey, you don't look as old as you are" and they ask me "how old are you?" 84. My mother died when she was 92, my father at 79...as long as I go forward 24'50, you can't realize it anymore, like when you were young. Because when I... I used to go for a walk to Nazareth and now RISAS I have a friend who used to go to the West Park a lot, we used to go there every day and little by little the man died and I... soon after I had an operation and now...

Intervention of the daughter 27'10

M - 29'43 XXX said that it is necessary to get used to.... 40'37

M - Do you think you have help?

E7 - Man, the truth is that I don't have much help....

SILENCE

E7 - Because we have a son, but the wife... and she had an operation...

DAUGHTER - A year after you had your operation....

E7 - He (the son) came to see her once, he was there for 10 minutes and he never came back. He called me on the phone and nothing else 41'08, (there is sadness in his words)

**E8**

E8. That's how we started. He fell and I was the one who picked him up, I don't know how my right bone is, I can only tell you that my arms are not mine because of the pain I have. I've had some very big downturns and I've had a very bad time, now I'm a little better. But before he was on the couch for five minutes, then I would sit him in the wheelchair or there [points to the couch], and he would say "I can't take it anymore, I can't take it anymore", I had to pick him up again and put him there [points to an armchair], all that was a burden, a burden because he didn't help at all, his legs couldn't take it, they stopped cooperating. Now, in the last fall, I said "I'm going to the procession here [in the neighborhood]" I've always liked to see the Virgin go out, period, I don't go to the procession or anywhere else and that night my daughter's friends told me "where are you going E8?" and I said "well look, I'm going to ask the Virgin to see if she can make it good for me tomorrow, or if it can be today even better" ufff..., ufff.... 1`16, what did you say, when I got home I found him lying on the floor with a broken hip. The ambulance came and they told me: "yes, yes, it's a broken hip". We took him to XXX, they operated him there. He has been two more times, [in XXX], but I don't know about the other one because it has been, the truth is that I have forgotten, but well. They operated on him and he has not put his foot on the ground anymore.

Mar. And... what do you do every day with XXX? What is a normal day like for you?

E8. I get up, wash my face, wash my hands, simply and I have breakfast first because I am diabetic and if I don't, I fall down. The first thing I do is have breakfast ..... I often say "how selfish", but he is sleeping and if something happens to him and he catches me fasting, I fall down, I can't, you know what I mean? then I prepare his food, I make him a bowl like this [points to the size] of cereal porridge with milk and I give it to him, he eats his breakfast, he drinks a glass of water, the drops, and the pills he needs, then, he stays quiet for a while and then it's a matter of washing him, getting the dirt out of his ass because he doesn't even have the strength to do it.....

Mar. Does he eat by himself?

E8. No, no, he doesn't do anything, nothing, nothing, nothing, you have to take the dirt out of his ass, you have to change him, you have to wash him, everything, everything, absolutely everything, I take the snot out of his nose, I'm sorry if it's ugly ..... I fix his ears, his hair, I do his hair, I do EVERYTHING, and then, if I feel like it, I do something [she refers to the house] and if I don't feel like it, I don't do anything, because I have had a very big downturn, I have had a very bad time [depression], now I am more peaceful because I have seen that I am leaving. The doctor came and told me "you yourself, or him or you, whatever you want" I don't go out anywhere, I don't talk to anyone and I have been like this for a long time, for a long time. XXX has given me a lot of torment, a lot of torment and "come, do this and XXX, XXX...but loudly eh ¡!!!!!!XXX¡¡¡¡

!!!!!XXX ¡¡¡¡¡¡ all the names that came out except mine, he never called me. Then, I give him the food and dessert, the pills and another time he is calm. At 6 p.m. I go back again, give him his snack and look at him [the diaper] again, if it needs to be changed, it's changed and do whatever he needs to do. I have to wash his eyes, the first thing I do every day when I go with him. And then at night I do the same thing again, so every day the same thing. If I feel like it I sew a little bit, if I can I dust and so on because I really don't feel like anything because my head hurts a lot .... I don't feel like anything, I lost my appetite and I have been terrible, in fact I am still bad because I don't eat meat, I don't eat fish, I don't eat chicken; terrible, a disaster, me, a disaster, that I am standing up because I have always said "my God, half an hour after him, if you have to take me", I don't care, I have done a lot of service to my daughters and I don't mind anything, but, that he leaves me until the last minute so that I can take care of him.

Mar. And you don't have any help?

E8: No, none.

Mar. What do you think, E8, about this situation?

E8. What do you want me to do, honey, I can't afford the luxury of where I get it [the money] from, with the pension of a pensioner, who gets crap in this day and age, much less than 1,000 euros, and my daughters, they have enough to take care of their two children. And this one [she refers to the daughter who was with her when I arrived] now goes to the office until 9 o'clock at night or whatever time it is and the other one leaves in the morning and also arrives just in time, sometimes when I have needed it, I have told her "baby don't come, I can manage on my own, don't come" because of course, with these arms I can't raise him up in bed on my own, and what is to put him in the middle of the bed, I can't do it.

Mar. Of course, the posture changes.

E8. I am having a terrible, terrible time, but let's keep going, some days better, some days worse, that is the life I lead and the one I have had, I go to the doctor, to the pharmacy and to Mercadona.

Mar. That is the life you lead every day.

E8. Every day. I feel like cleaning...yesterday I cleaned almost three parts of the kitchen because it's big, I hit my head, thank goodness I didn't hurt myself, thank goodness, because it doesn't hurt at all. But I nailed the doorknob here [points to the side] and I said "My God, thank goodness I have him [looks at the sky]", that's my life.

Mar. Does XXX leave the room?

E8. Yes, he gets up, well he doesn't, I put him in the wheelchair and he holds on for 10 minutes maximum, while I shave him, clean the room and that's it, stop counting and run back to bed because otherwise he gets tears running down his eyes, water drips from his nose, drips from his mouth [points to his mouth]; and in bed he doesn't want anything. He only wants to go to bed, that's where he's best, honestly, his head falls [on the chair], now you'll see, if it doesn't seem so, if you don't see his body, it doesn't seem so bad. 8`27

Mar. It seemed to me that you have said that he names other people but he never names you?

E8. Yes, but that was a long time ago. Yes, but he doesn't want me to leave, "the mommy". Sometimes, look, look how I wear my hair [shows the lack of dye], I can't go to the hairdresser's, or anywhere and sometimes, my little daughter tells me: "mommy, please go to the hairdresser's", she comes and stays with him. She tells me that XXX is all the time "where is mommy?" and he doesn't speak, because he doesn't speak either .....

Mar. Do you notice that he doesn't recognize your work?

E8. No, he doesn't recognize it

Mar. Was it like that before?

E8. Yes, let's say he is a very good person, very polite, you know, a very polite gentleman, very correct, but he has not been cheerful, nor talkative, nor ....., nor affectionate, no. I am much, I am the opposite. I told him, I promised him from the beginning .... because one of his sisters was put in a very nice place because she opened it for the first time, but heaven forbid, no, I said no, no and no...

Mar. Are you referring to a residence?

E8: Yes, it was new, but when I saw how it was going, I said no. I came out of there in tears. I was crying my eyes out when I went there and I told her "no, my dear, no, even if my life is at stake, you will be with me until the last minute". Everyone can think as they want. It is something that if I took him anywhere I would not live and then, it is not worth it to me, you know what I mean? I would neither sleep nor do anything, because no, no, it is something I would not do.

Mar. Are you calmer taking care of him here?

E8. And better [care], yesterday I changed his bed, I changed it today, tomorrow I might have to change it again. He wears diapers, he wears the connector and the bag, but what happens, sometimes he leaks [urine], so I prefer that he doesn't leak, because otherwise, the sore...I have had him very sore and now he is better, very well, but it turns out that he had another little sore on his dick and we left him a few days without the connector and then, his ass has been making its way again, having it wet all night long.

Mar. Earlier she told me that she had applied for some benefits, help ...... 11'33

E8. Here came the one from the dependency law.

Mar. The social worker?

E8. No, not the social worker, first they send one and then they send another one and of course they gave her the worst grade, of course, a person who does nothing....

Mar. Very dependent.

E8. Totally dependent and it is going to be 3 years now, because we have put him there since he fell, not before, eh.

Mar. Have you heard anything back?

E8. They offered me a woman for an hour and of course a woman for an hour, honestly, does not solve anything for me and on top of that I had to pay 10 Euros and I said, "I don't need those favors", so....

Mar. Do you have help from the neighborhood?

E8. No, because look, here [she points to the house next door] there is a guy living alone, and if he can say good morning, he says it and if not, nothing. I went to ask for help, because I had my husband lying on the floor and I couldn't and I know he was inside and he didn't open the door. And the others, no one, when I need someone I have no one, I go to lend a hand and I say: "no, nothing, when I need something the sky sinks with the earth".

Mar. And his daughters?

E8. My daughters are there, yes, of course, and my grandson, one of my grandsons, is ... What happens is that they are at their jobs and at universities and have hours that cannot be, but I do need it I think it's coming, well, how many times have you picked it up and put it to bed, yes. But that's it, they can't do anything else to me because they have to leave, of course. I understand it, because what are the creatures going to do if they are doing their thing? But come on, I'm not expecting anything anymore, eh, I'm not expecting any help because ... they haven't even breathed. The supervisor in XXX is a beautiful person, XXX is a beautiful person, she has insisted a lot, a lot. My daughter, as her husband was a lawyer, knows a lot about the issue 14'15 and goes many days to present papers and things, and they told her: “you know what I'm saying, don't suffocate anymore because they won't give you nothing"

BE QUIET.

Mar. How do you live this?

E8. Well, I think that if I look back and there are those who are worse, honestly. That if I know that they are not going to give it to me, they are going to give it to another person who needs it more, so far at the end of the month I arrive, but not, eh, but fair would be fair, it has worked 45 years, that this writing huh, without being written anymore huh and it is very funny that "if you pass, very good and if you do not get anything", no, then that they give me back what has been left over to him, don't you It seems?

Mar. Well yes. Have you always worked at home?

E8. I've always worked a lot, dragging, I've lived dragging like dogs, you can see that, I say, "I don't know who I've been so bad with because I don't understand." All my life since I was 8 years old I have been scrubbing, since I did not learn anything else, all my life, since I was 8 years old, that I got wet up to here [points to the neck] eh, until I marry XXX. When I married XXX, I do not know how many more years I would work because they were both single and both with that of getting married, then nothing, but since I have years left, because I worked insured for a while but the rest more than possible, I He had the keys to 11 houses and part of it went to two offices.

Mar. Sure, but when it is worked without contributions….

E8. So, if that is not a sin, huh. There are times that if I have tantrums when I am low in morale that happens very often, now I have been bad for 2 or 3 days again, I am not excited about anything, nor am I happy about anything, or anything, but like I never did. the illusion that I was going to charge it, because I am not disappointed.

Mar. Are you referring to the dependency law?

E8. Yes, I told the girl who came that I would like to remember who she was, I was going to answer her and I told her "all this mess is for nothing because they don't have to give me anything." saying?"; I say "people who have done it before me" she says "oh well tell them to buy the same" eh, now I could say "what do we buy?"

Mar. Well yes, the truth is that it is a very complicated situation.

E8. I really miss it huh, I really miss it, you know, I really miss it. You know what it is like at 2 in the morning that you fall out of bed with 82 kilos that you weighed, all peeing, all dripping, that slipped from me, of course, I could not handle it, nail it, change it ... I have spent moments One of those, I don't know how he hasn't killed himself, he was holding on to a door, he broke it, that one tore it from me, the one in the bathroom, when he wanted to catch himself, he had ripped them off, ufff… it's very cold here, it's all done , very cold and very hot and then we put a mat for the feet. Well, five years is without putting

BE QUIET.

Mar. Do you heal the sores?

E8. Yes Yes.

Mar. Has anyone taught you?

E8. Not because he knows what happens, that I am very collaborative. I am so bad, that I spent a long time with my friend's husband who fell from a third floor and has been a long time, he already died but he has been, he has lived more than 16 years and I have helped him a lot, a lot and then I know how the subject goes.

Mar. Know the techniques, how they are done. I ask them because sometimes they don't teach us. He remembers that before he told me that he learns by doing it ...

E8. Yes, yes, it is true that way you learn. Now the doctor loves me, the nurse loves me, the supervisor wants me, she loves me all Christ, I say, "Well, I won't be so bad then." I think that I have been very bad because it is that daughter I have led a life so dragging eh, really dragging, the first one went wrong, I passed those of San Amaro [blows] and it has been everything, my life has been like this My daughter had to cut both breasts, not because of cancer, eh, not because of cancer, she did not have cancer, she had surgery because her breasts were full of cysts, and the gynecologist who was taking her told her, “Teresa Didn't you want to fix your chest a bit? Well, take advantage and have the most of it taken away from you ”. She went to doctor XXX. She is standing because I think they listened to me so much [she looks at the sky], that… they cut off both of her breasts [she sighs anxiously]. She was given a prosthesis that was not appropriate and she, my daughter was ill and I said to her "baby, are you wearing that well?" 20'45 and she said "Mom, I tell the doctor and she says yes." I never go anywhere, I never go out, but look where, they invited us to a paella and my mother-in-law and I left and while eating, my son-in-law showed up there, and I said "what's up XXX?" , the one that has died, and says "nothing that we have gone to give the animals food and we have said go, because we get them off and then they don't have to get off on the train." I didn't have them all, but hey. Well, it turns out that the good lord [the doctor] was taking her, he was taking her and it got worse and worse, worse and worse until one day they exploded like two grenades, squirting pus everywhere ... something to burst … .And that day is the one that I had left. She went in search of her sister and when her sister saw her come in, she wanted to die "Tere, what's wrong?" He does this [pretends to uncover], and he saw how it was going, he said "let's go quickly to the XXX that one" he takes her and tells her that "to move on from how cumbersome it was that tomorrow she would go to the clinic that he would cure her and leave pa his house". My little girl says "I looked at him and said this guy is crazy" but since he saw her so badly, he entered a bar and made her drink a lime tree, she tells her brother-in-law and a friend who also works in XXX " ale to XXX ”. They go to XXX and they caught it right away. "My mother, who has done this to you, but by God." Well, they were stretching their hair huh. In total, they could not operate because she had taken the linden. When the necessary time passed, she had everything prepared and they put her in the operating room, and when she left the doctor said "that the operation had gone well but that both breasts had been cut off." There in her office they know because they also cured her. Well, nothing, without the two breasts, she or let's say how ... ..and the others, well I have led it like that, apart from the bad life that I have led that I have led very badly. Well, until she had it cured, they couldn't put a prosthesis on her or fix it or anything, but since they wouldn't let her, they couldn't even put a prosthesis on her. They went to operate on her, they opened her from here to here [points to her chest and abdomen] to put her meat on her chest and since he had it on her, he did not ...

Mar. Was there rejection?

E8. Yes, he is ...

Mar. Did she win?

E8. If I dont know….

Mar. Something happened.

E8. Yes, he operated on her three times, she was in the operating room for 14 hours. We were all there ... not him, he was already pachucho and totally, they told him nothing. The next morning he actually went to the operating room again, again with nothing he left XXX. When he was well healed they called her but with the disaster that he had they could not do wonders. The doctor, said "princess what they have done to you." He tried to do the best he could, but he says "my queen, as much as I've fought, I can't do anything to you." Well, a disaster. Luckily the poor thing then, she has a spirit that we are going, she will spend the time with her, but…. And so she has stayed, because she doesn't want any more surgery.

Mar. E8 What does she think about the life that she has now? As she has explained to me, with life so complicated that she has had, what feelings does she have now?

E8. Feelings? Well, I can't explain it. I have had a very bad time at first, now I take it more calmly.

Mar. Do you think she has adapted to the situation?

E8. I think I have adapted, and I do everything with a lot of affection, that is not lacking because I have plenty of affection, I thank God I have affection to give to everyone and now I take it very badly that I cannot talk to anyone.

Mar. Missing note ...?

E8. Yes, a little bit, I am not a street person, you know, I am not a street person either, but yes, a little company, to be talking for a while, or to go here or to go there, which is the only thing they recommend but, I go and leave it alone, it is not born to me

Mar. Sure, from what you've told me, it's a problem, you can't be alone.

E8. Me, not to go with joy and with that illusion that it should be, because I'm not going anywhere, because here I am calmer.

Mar. Is that tranquility important to you?

E8. Yes, of course, I need a lot of tranquility, to suffocate as little as possible because I have a very bad, very bad, very bad.

Mar. It also seemed to me that you need him to recognize his work, you need more affection.

E8. Yes, but I do not take it into account because he has bursts that maybe he says two words or bursts that he just looks at you and looks at you and that's it, I don't take it into account either, poor thing, it hurts me so much….

Mar. Why do you take care of it?

SILENCE [takes time to answer]

Mar. He could consider himself, I don't know financially or ... that someone help him.

E8. I can't financially, but even if I could, I don't want anyone.

Mar. Why do you take care of it?

E8. Because I love him, why not [a residence], because he is not born to me. The time he has been with me since I got married, 28 years we have already been, with me he has not gotten into anything, affectionate and that has not been, I am more affectionate than him and that, but with me he has not gotten into anything I I never cheated on him in my life, I told him "look, I have two daughters and my daughters come first before all things in the world", I cheat none and it is true, my daughters and I have four grandchildren that is to die for, but I, if I say that I married him because I had fallen in love, I would lie, filthy lie as it sounds, because it is many years, they are 16 years apart, eh, and that is a filthy lie that they tell me "in love very much", lie, lie . Over time I have grown fond of him and that but I did not have social security, to tell lies is to deceive yourself, I had nothing more than what I earned per day I did not ... and with him I have had these years for what less an assurance that when the end of the month comes at least to eat I have. Why am I going to tell fairy tales if that is a lie, today, that no one touches me, you know? Not today or before, eh, that is clearer than water, but to say that one falls in love; that arises once in a lifetime.

Mar. Care for gratitude?

E8. Gratitude? I believe that it is my obligation, I do it as my obligation, he is a person who has loved me and has behaved very well with my daughters and with my grandchildren and perhaps that is why no one touches me and my daughters love him with madness and my grandchildren the same, they want it madly ufff. 31`01

Mar. It is important, I believe that in order to care it is important that there is a good relationship.

E8. Yes, but look, my friend's husband, I had no relationship at all eh and I took away the dirt that he carried by the cone and…. And all the things, because this mine is not dirty, it is not a dirty person, it is a very hygienic person to be honest, but I have been with people who care for them and I have treated it and if there has been someone who has been able to lend a hand I have given it, honestly. Because I can't see a person suffer, I can't.

Mar. E8, how do you relax or distract yourself? Do you have any entertainment?

E8. For example, I like to sew, whether it be worth it or not, but my back hurts a lot, nowadays my arms I can't with them and now I do it less but I relax ... not reading, because my eyesight although I had surgery I take it very badly from the falls, I don't take it well, I have never liked reading in my life, never.

Mar. It is not something I did before and not now.

E8. I see the fat letters and that's it, I have plenty of others, I know how to read but if I haven't been to school, what do you want?

Mar. She likes sewing and cannot spend as much time as she would like.

E8. I put on and I forget about the world, let's say hey, I relax.

Mar. But now do you sew?

E8. Yes, I have my grandson there, "iaia iaia cut my pants, change a zipper" "Yes darling" The other comes "mother-in-law, I'll bring him work" and he brings me to fix his pants….

Mar. he does things you like

E8. Yes, yes, I like the truth is that yes 33`14 and that's it and stop counting.

Mar. E8 Have you learned anything from taking care of her husband? Have you discovered something about yourself that she was unaware of?

E8. What did not know? I have a lot of patience, a lot of patience, what I say is that to be so nervous that I am, however I have a lot of patience with him, he will never hear that I have yelled at him, nor that…. I have not yelled at my daughters and I am very nervous. Now, that he calls me "Jewish dog" and right away ufff [makes an angry gesture], but I'm not, I'm quite relaxed, it will be because in time, I don't know, I don't know how to explain it either. When I fall down [down, depression] we already have it involved.

Mar. She also has her low moments ...

E8. Very bad, very bad crises, I begin to feel like nothing, nothing, nothing.

Mar. What does she do in those moments or in those times?

E8. Well, I had to go to the doctors several times, for example, I went to the hairdresser and when I left, I could no longer come here, I called a lady who was passing….

Mar. What happened?

E8. She was dizzy and I was falling, it is seen that she gave me an anxiety attack and the lady brought me. They called the emergency doctor and when he came he said "ma'am, you can't take this march you're taking", I said "well, explain to me which one you want me to take, this is what there is."

Mar. The truth is that it is very complicated.

E8. She says "that march is too much march." She put a pill under my mouth and she gave me another to take, and she told me "yourself, or he or you", but it is that three doctors have told me the same thing.

Mar. What do you think ...

E8. That they are right, they are right, but what do I do? Do I throw it away? I will take it anywhere, they can tell me in pink, that I will not take it. I'm not going to take him, if not something happens to me, God forbid, as long as my life is and I can stand up, he stays here with me, well, I would not have anything else as clear as that .

Mar. The truth is that at first glance you are very young

E8. At first glance, yes, now I am 10 years older, this has ruined me

Mar. Have you noticed the passage of time?

E8. Of course I notice, now if you want, let's go there and see it, his face doesn't have a single wrinkle and yet all of him [talks about the physical body] is a skin 36'45

BE QUIET

Mar. The truth E8 is that it is a very hard life

BE QUIET

E8. Horrifying. Sometimes I think, pa what? ... pa na., Now, from now on that ... what do I have left now?

Mar. Do you think about the future?

E8. Let's see ... what remains for me, to give my daughters work? I ask you with all my heart that God forbid it because I know what it is. My mother had 15 years with Alzheimer's.

Mar. Did you take care of her?

E8. When it was my turn, the month it was my turn "and he hadn't behaved well with me eh", and I wore it that looked like a rose eh, just so you know and I sometimes say "Lord, I've done so badly, that opens done so bad, I don't know, I don't know ”.

SILENCE [teary eyes]

Mar. E8, people love her, because they have also told me about it, the supervisor, her daughters, her grandchildren, her husband, it is a very important value.

E8. Yes, to me, the other day who told me? from the office… the social worker, I love her a lot XXX and I say “it's that XXX to XXX and I say I love you very much”, they treat me very well and say “E8, take into account that sometimes it is not us, it may be the patient too "

Mar. Of course I do

E8. And I say "well it will"

Mar. It is the good thing that you have, the type of relationship and the affection.

E8. If I want them, the truth is that I love them, it seems a lie but….

Mar. And the help you have when you need it. It's very complicated.

E8. The social worker said the other day "don't hurry", because the computers had broken down and they couldn't give me the medicines for me. And the social worker told me "until the last minute, if it works out, you don't worry", and they gave it to a girl who lives next door and when she left she brought them to me, and I was grateful.

BE QUIET

Mar. Well if [E8 seems tired] 39`42, if she has nothing else to tell me?

E8. Well I can tell you all that

She wants me to come see her husband. The room is small, with lots of natural light, clear of furniture and against the wall. The bed is on some studs, it smells of NENUCO cologne (very frequent to perfume the elderly). She uncovers her husband to see how thin she is, XXX does not respond to any stimulus. She speaks to him with affection and tranquility, proud of the work done, while she covers it again, she tells him: “he deserves everything”.

**E9**

[E9 walks with a cane is overweight and mobility is compromised]

Mar How is a normal day taking care of XXX [sister-in-law]?

E9 A normal day? As I have the woman who comes [assistant], because she comes at 8:30, picks her up, fixes her and I am aware of everything, so that she does it well, washes her, fixes her. I give her breakfast, she has breakfast, I put the spray apart from all that she carries [E9 points out to her sister-in-law that she is connected to a very large portable oxygen pump]. Anyway, a normal day, later, she sits there [points to the sofa where her sister-in-law is] at lunchtime and in the afternoon at 4 o'clock. I wake her up "before she rests a little bit" I give her a snack from 4 to 4:30 a.m. because at night at 8:30 a.m. we already have it arranged there. This woman [the maid] is also waiting for the night, “now she has gone out with my husband”, my husband also has it annoying and since he does not go out because now he has a little while he goes with her to walk around. That is a normal day. [Attention to the tasks that the maid does, she is Ecuadorian, she only pays on Sunday]

Mar. Very well, then, is the person who makes the physical effort to put her to bed, accompany her, lift her up to her sister-in-law ...?

E9. She is fair with the tacatac

Mar Does her sister-in-law of hers if she can move well to lie down, get up?

E9. She moves with the tacatac. When the maid doesn't come, I fix it myself as I can.

Mar. So the person who helps you is not there every day?

E9. Yes, every day except Sundays.

Mar. On Sundays, is she not there?

E9. That, on Sundays does not come.

Mar. The food…?

E9. I make the food

Mar. For all three Do three people usually live?

E9. No, not the three of us, four of us live and the lady five, I also give her food [The son, the partner, the sister-in-law and the housekeeper]

Mar Is she internal?

E9. Not

Sea Don't you sleep here?

E9. Not

Mar. This person eats here, normally there are five people, less to sleep than the maid is not there.

E9. She comes in the morning, leaves for a while in the afternoon [she walks with her husband] and then comes again.

Mar. Very good, great. She has told me that the questions of food, you ask?

E9 At the moment, yes, because I am on the plan [waiting list] that if I have to operate on my knees and of course, I am interested in having a woman like this I have for myself to be able to use, although I also have my daughter who can throw me out a hand, although she is not at all, but anyway.

Mar What has a daughter and a son?

[Nods nods]

E9. What can I tell you? What else?

Mar. I'm also interested in knowing if the type of help she has is private or public. To the person you have Do you pay or is it a resource of dependency law? 3´31

E9. It is of the dependency law, but we have been since August that we have not collected.

Mar. As things are, it can be said that it is even “normal”, but isn't it a resource that you pay for privately?

E9. I'm not going to leave her without paying the poor woman !! [Laughs, laughs]

Mar. No, surely I have not explained myself well. You have this resource because it has been granted to you, obviously now you are not being paid and you do not stop paying your salary. But, it is a resource that has the state by the law of dependency.

E9. Of course, yes, from the law of dependency. Besides, I have my husband's pay that I want to tell him that even though we're going tight, because let's see, you have to go tight, today as you are.

Mar. Do you only have the resource of the assistant or do you have another type of help?

E9. No, no, only this woman

Mar. And this woman, does she work the hours she is telling me?

E9. She comes in the morning and there at 2:30 a.m. she leaves and then at 5:30 a.m. or a little more, she comes, she takes my husband and they go for a walk…. [Immigrant caregiver theme. I perceive that there is something that is not clear related to the assistant because as an institutional resource it is very rare that she is there all this time. In addition, she is being used for other tasks that are not those of caring for the sister-in-law. It could be a topic….]

Mar. E9, I ask you because there are times when families, caregivers are given 1, 2 or 3 hours of help by dependency law, but the caregiver needs more and then they are paid separately (privately) to be in the afternoon. Do you understand why I am asking you? To know if the resource is all the time that is helping you or is it a part and you pay the rest. That you have told me that the appeal is not complete.

E9. Man, I complete what they give me, it is not enough, that of course.

Mar. Okay, but what you have is what they give you by the law of dependency.

E9. Yes, yes.

CHILD. Can I speak and clarify? 5`46

Mar. Yes, yes of course

CHILD. It is because of the dependency law, she [her sister-in-law] has grade III [great dependent] it is 416 €, what happens now is that they are going to reduce it [she refers to the economic benefit]

Mar. Yes now it has changed.

CHILD. In the letter they have sent us, it says that according to her [sister-in-law] heritage it will be even more reduced, so I don't know what it will stay on. Now 15% less, they are € 60 or € 70 less than what she charges, she will stay at € 350.

Mar. Claro 6'11 I ask him because ...

CHILD. Besides, until now the dependency law assumed social security, that is, they paid for the girl and now they have taken her out, since September 1 they have taken it out. So, she has had to us to discharge her by contract to assume social security.

Mar. For this reason, not in this particular case, but ...

CHILD. So, I also live here and the contract is in my name, of course, what [the maid] charges we pay between the four of us.

Mar. Yes, yes

CHILD. And this [the economic resource] is a little help that comes in handy, the subsidy of the dependency law.

Mar. Claro 6`50

E9. Her [sister-in-law] pension also goes in. A little bit from here, a little bit from there and we are doing things.

Mar. Yes, the resources you have ...

CHILD. The girl only came in the mornings for 5 hours and since October 1 she also comes in the afternoons, due to my mother's knee problem.

Mar. Asking it is because there are times when the people I interview say "I have a person who comes all day" and it turns out that later ...

CHILD. Until October 1, she only worked in the morning, that is, in the afternoon she [the mother] fixed it by herself. She [the housekeeper] ate and left at 2:00 p.m. She leaves on Saturday morning at 1:00 p.m. or so and she [the mother] stays until Sunday morning. My sister who is retired due to illness with 50 years and she is also not at all.

E9. Three and a half years like this.

Mar. This is important to clarify because when analyzing the interviews it turns out that the resources provided by the dependency law are one or the other.

CHILD. I believe that with all this, the law of dependency has been charged.

Mar. Now there will be changes again.

E9. Everything is impossible, I watch TV and I am horrified, I say: "Lord, how will we stay, with a war again", I would not want to see it.

Mar. The situation we are experiencing is very complicated.

E9. Much much. 8`51

Mar. E9, you do certain things to her sister-in-law, for others, she has help. But her sister-in-law, what can she do?

E9. No, what can you do? No, you have to dress her, undress her, you have to clean her, wash her, you have to give her everything, well not the food, she eats her.

Mar. Does she eat alone?

E9. Yes, she eats alone.

Mar. On days when there is no maid, do you do it?

E9. Yes, I do it and I don't know what I can tell you more.

Mar. You supervise the house, but you don't.

E9. From the house I always do something, “look, you haven't done this well”, or “do it to me like that”. She is a good woman, she helps me to tend, of course, she must help me because I cannot do everything. Quiet, I'm not there either.

CHILD. On Sundays [the mother] picks up and mops

E9. I have a character that I can't stay still, about being 82 years old, well no, I will soon be 83, next month I will be 83, so ...

Mar. Well, it's great

[Laughs] 10`28

E9. Now what I need is to lose 15 kilos

Mar. Well that too, because of the intervention it is important that you lose weight.

E9. Well, I have a tremendous desire to eat, you can see that my nerves make me want to eat and I try but.

Mar. Losing weight is not easy. Have you told me that you have been taking care of your sister-in-law for three years?

E9. We are, we are, more, she has done the three years in June, that is, everything that goes from June to here. 11`09

Mar. Have you always had help?

E9. As much as now

Mar. What has caring for your sister-in-law meant to you?

E9. Being my sister-in-law then, having to do it.

Mar. Why?

E9. Well, because she is my sister-in-law, she is a person, that she is with us since she retired, that she worked and since she retired she is with us and I have done it at ease. What happens is that of course, because there are times that, maybe she tells me, I tell her [laughs, but they argue] and so on.

Mar. Well it seems that these situations fall within the normal coexistence

E9 Well that.

Mar. Has your sister-in-law then always lived with you?

E9. Yes, she has lived with us and I am telling you, once she retired, she was here. Later she got sick because I pricked her too, although I am not a nurse, I prick. I put insulin, I also do not know if you know it, a product that was calcium that is put in the belly, I do not know if you know it.

Mar. Not putting calcium in the belly, but there are injections that are put in the belly, yes.

E9. I don't know if it was too much what she put on him, but that's when she started to not be well, well, it was too much calcium what she had and she started to feel bad. In addition, she has had the bad luck that two by three a blow has fallen, she knows, Nothing has been done because nothing has ever been done, but if you are careless you drop it, that is, you have to be aware of it, it goes with the tacatac but you go behind it keeping watch.

Mar. If watching that she does not fall, Has she ever thought that her sister-in-law was somewhere else that she was not at home?

E9. Well, I haven't thought about it, my children have thought about it, because I don't want him to go. There we have the fight, because my children want me to take her but I do not want to because she in her right mind does not want to go.

Mar. Does she understand and understand when they speak to her?

CHILD. Her problem is hearing, she does not hear.

E9. She has everything with knowledge and she doesn't like to go to those places and I'm stupid because I don't want her to go either [laughs]

Mar. So you take care of it?

E9. Sure, that's the way I am, this way, finishing me before her [laughs]

Mar. There are tasks that this is what you have

[Laughs]

CHILD. My mother cannot go out, nothing, she has taken her life.

E9. Here I am night and day.

Mar. Do you have friends?

E9. Yes of course

Mar. And they go out?

E9. They, of course [look at her son]

Mar. They What about you?

E9. I do not, no, I do not go out at all, I am here nailed every day, Sundays and holidays, and everything.

Mar. Why doesn't it come out?

E9. Why don't I go out? Because she can't be, I can't leave her alone, as soon as you leave her alone she does something to you [Laughter] she gets up. She cannot be alone, I cannot leave her alone. They go, for example, to soccer 14'52 for my grandson, I already have a 16-year-old grandson and they go to soccer and I stay with her.

Mar. Has it been like this since she's sick?

E9. Always, since she fell ill huh. In XXX she stayed for about a month and then they told me: “either she takes it home or she goes to Porta Coeli”, they told me that there was nothing to do. This has been three years and I still have it here [laughs].

Mar. Medicine is also wrong.

E9. No, no, he was not wrong, what happens is that the care he has gives a lot of life, the care he has is different because they don't tell me that if he had gone to XXX, the care he has had he would not have had. I am aware if she is missing this, if she is sitting, if she has the feet on, pending everything, too much, that I am consuming myself.

BE QUIET.

Mar. And her husband, does he also go out?

E9. No, my husband is here too, he only goes out for a short while [he goes for a walk with the maid every day], or he [his son] goes off to soccer. He goes out more than me. Even now on Sundays I hear mass on TV, which I used to go down, but since my legs are not going well, because I have to get annoyed.

Mar. Now why does he get together in addition to having to take care of his sister-in-law, the physical problem you have with your legs, but would you go out?

E9. I would go out, but I am afraid to leave her with him alone [her son] because, it is enough for her to know that I am leaving to make her want to go to the toilet, to make her want something and "where is E9?" and "where is E9?" and “where is E9? I want to go to the toilet ”and of course so that all that does not happen to them [family] because I stay here.

Mar. There is a significant demand from her sister-in-law when you are not there, do you see this happening?

E9. Exactly 17'18 she knows that she has everything with me and that many times I deny her and tell her "little by little you give me favor because you make me talk and" well ... that

Mar. That what?

E9. That, well what happens, she tells me, I tell her and so on [Laughter]

Mar. But this would be normal

E9. Man of course, because then nothing happens, I am not capable of hurting him, so I put everything on him, the pot, everything. Do you want to see everything I give you?

Mar. No, I don't need you to tell me what pills or medication she takes

E9. I mean that if I were bad, she would not live even because I do weird things….

Mar. People who depend on others, that helplessness.

E9. What a horror because, in addition, he who does not believe is different because a person who does not believe is not afraid of God, well ...

Mar. Is religion, beliefs important to you?

E9. For me a lot, each one, exactly each one believes in his own thing and each one does what he should do, do I believe? because I even say to the lady that I have many times "you see, I deny you, for not doing this well", as a sign that I love her and I have to take care of her, so if I didn't care, She [the assistant] has done it for her, because there she….

Mar. Why do you take care of her sister-in-law?

E9. Simply because she has no one, nothing more than the family that we are and she is a person and we have to take care of her. I've always liked the old, the children and the old, but of course, now I'm getting a little fed up [Laughter] and my age.

Mar. Would it also be within normality? Logically, you will have noticed the passage of time in her body, in the way she sees life ...

E9. In everything, in everything, there are times when I think, "Oh Lord, I deserve so much Lord" but life is like that and I have to abide by it.

Mar. Do you feel that the work you do is recognized? 19`54

BE QUIET

E9. Man, I do things without looking, I don't know how to tell you.

Mar. You feel grateful from ...

E9. Of the sick person?

Mar. Of the sick person or of the people who live with you, why if you don't…?

E9. Well, I don't know what to tell you, they do recognize it, they [looking at their son] do recognize it but they are not one of those who tell me things, I speak for myself [he looks at his son again]

SON- Do we not recognize you? She, she [goes to her mother and points to her aunt] is the one who doesn't recognize you.

E9. Let's see, admit it, they know too much. Because I also have to shower my husband, he has many things, he has prostate cancer, he has a heart attack, he has Parkinson's, he has heart attacks and he is taking sintrom, well ...

Mar. So you don't just take care of her sister-in-law? She is also taking care of her husband.

E9. He stands alone [cannot perform ABVD]

Mar. Do you supervise it too?

E9. I put the socks on him …… I have a something hanging there [points to the back] [Laughs]

Mar. A ballast, a backpack like mine [I carry a backpack as a bag, when I entered the house E9 told me “what a beautiful backpack”] that has been filled with little things….

[Laughs]

E9. [Laughing], That is life, each one has something to do, there is no choice.

Mar. And how do you experience this situation? What do you think of all this?

E9. There are!! I am happy, I am not one of those who "oh, oh I'm crying, I'm not"…. There are times that my husband sits here in the morning [on the couch] and closes his eyes and is all morning with his eyes closed, I tell him “Vicente picks up a book, read or watch TV and distract you, something”…. .I don't, I can't be like he, I'll do something, or what I'll tell him or sew, whatever, something.

Mar. The things that you say something could be things that distract you, that relax you….

E9. Sure man, I like to do things. I don't like being still, I don't like it, now I have to be still because of my leg, for a while I put ice on myself. 22`12. Back here in the lumbago it also usually hurts, I have two parrot beaks on my back. Anyway, I'm here to take care of me, but I don't do it like that, I do it the other way around. As far as possible, they take care of me because my son himself takes care of me and my husband, well not him, because you have to go after him, but my son does and my daughter when he comes too.

Mar. What difference do you think there is in the way you and your husband are?

E9. How do you mean?

Mar. You have told me, if I have not misunderstood you that you are a very active person, you even seek to do things….

E9. Exactly, distract me, not be ... I don't know how to say.

Mar. Instead you see your husband who is more apathetic

E9. But I attribute that to that it will be because of the disease that he has

Mar. Do you think the difference is due to illness?

E9. Sure, for all that he has, he has many things. Before he pricked him "I don't know if you know that, it's three in three months", an injection for prostate cancer, I also gave it to him. I have prodded my children when they were little, since they were little I have prodded them and now my husband. The urologist has taken the injections and it seems that he is doing quite well because before he gave him the injections and it seemed that he was more viscous but he did not have Parkinson's, instead now he has got Parkinson's, he is worse.

Mar. How old is your husband?

E9. My husband is now 77

SON- 87

E9. 87 and I say 77, if I'm fine ...

Mar. She had taken a lot of years from him huh

E9. He had made him younger than me and they are 5 years older, we are 5 years older, so if I am 82 then he is 87 years old, on his way to 88.

Mar. From what you are telling me, has he been taking care of him all his life?

[Laughs]

Mar. Still, he has not been able to rest from caring….

E9. [Laughing], don't make me laugh.

Mar. Is that so? She has taken care of her children

CHILD. To the grandchildren ...

E9. I have raised my grandchildren too.

Mar. Take care of the husband, the grandchildren when he has touched you, I say take care, you are telling me to raise eh that this is already a higher degree, you are taking care of his sister-in-law.

E9. My daughter worked and left me the little girl "she is 22 years old" [the granddaughter]. I was 60 years old and she already had it all day, they left me in the morning and at night they picked her up. So that's my life….

Mar. She has experience of caring for her throughout her life.

E9. That's my life [laughs]. I'm totally confessing like I'm confessing huh.

[Laughs]

Mar. It is great for me that you go to confession because the more we talk, the more things I can analyze. Although I have to tell you that deep down in the interviews ...

E9. I mean that after all this nothing happens to me ...

Mar. What's going to happen to him? And if something happens to him it will be good and I learned a lot. Normally interviews with women of your age and who have had a life similar to you have similar situations ……

E9. Look, now I'm going to explain to you, now for example, we have a group on Calle Alboraya 26'02, so I told you that your face was known to me because I lived on Calle Alboraya. We had a group of friends who met in a bar once a month. Look, we would go there all afternoon, we would sit at least fourteen or fifteen, have lunch and chat, "well this, then the other", total ... But all that has been undone, because "one has died, the other does not know that "anyway. Dying, five or six of all of us who went have died, the husband of one, the husband of the other ... and that I can tell you ... Then I went out, although I had her [her sister-in-law], she was also better than now.

Mar. Now, do you have a relationship with those friends? On the phone Do you talk to them?

E9. On the phone, in the saints we congratulate each other later on Christmas too, we congratulate each other on Easter, what happens

Mar. Do you miss not having a social life? Going out, going to the movies….

E9. I am one of the people who conform to everything….

BE QUIET

Mar. What do you mean by "I settle for everything"?

E9. I am satisfied, I am satisfied. For example, my husband goes to football because they are telling me: "You have to come one day to see your grandson play, I haven't been there since the time he has been playing football" well, if I'm doing well, if I'm not going , also well…. BE QUIET.

Mar. Will he ever have to go?

E9. [Laughing] Well, I don't know.

Mar. He will have to prepare to go see him play, because of course, this cannot be ...

E9. Who stays here?

Mar. Who stays here? Well, one day they raffle it, put it here on the table and raffle it, whoever gets it or whoever gets it, stays for a little while. I think a game does not last that long ...

E9. [Laughter] The 3 hours sure, come and go.

Mar. I don't know, but how long does a game last? [I ask the son to get him involved]

CHILD. Two or two and a half hours

Mar. Well, that, three hours and so you have something there, set the three hours and have something. You will have a joy and your grandson for sure more. Sure, they can get around it.

E9. [Laughing] Anyway, what are we going to do, each one has something to do and it has happened to me like that, if I handle it badly it is worse, if I carry it with nerves it is worse. There are times that I carry them [her nerves] because of her [her sister-in-law of hers] who tells me anything but if not, I don't have the reason to be nervous.

Mar. What do you do when those moments arise? When is he bad or down? When is he sad

E9. What happens is that I, I don't know how you think, but I always praise God, and everything passes.

Mar. For you it is important and gives you strength

E9. Each one has his thing .... I praise myself to God and also to the Virgin

Mar. Comforts him

E9. That's right, I do. Each one is as it is. I don't have to hide from my thoughts [Laughter] I'm telling you all this and I don't know you the way you think.

Mar. Well, I'm not religious, but I respect and understand that there are people who share religiosity, a certain spirituality, it helps them

E9. Do you know what I'm saying?

Mar. This is the beauty, that there are people who have different beliefs and see life differently

E9. Well look, I for me, if I did not believe, my mother!

Mar. Would it be a problem?

E9. If she came and [her sister-in-law] were on the ground, I would not care, if she did not believe, it would be the same to me.

Mar. God and the Virgin comfort you and give you strength

E9. Me

Mar. Do they help you in times when you are unwell?

E9. If I scream, everything will also go away eh…. [Laughing]

Mar. It is also another way

E9. Anyway

let's do

Mar. Do you think that the passage of the years has made you adapt to the situation or is your character the same now as 30 years ago?

E9. I always, what will I tell you, I do not know how to explain it, it seems to me that they have loved me where I have been because apart from knowing how to DJ and all that, I have been a hairdresser and when I went to comb well, they loved me everywhere.

Mar. Was she going to comb the houses?

E9. Yes, but this before getting married huh and I have already done the golden wedding anniversary [Laughter] I want to say look if they are years…. Now we have done 56, way of 57 years.

Mar. Geez it's been years, already

E9. In other words, we have made the golden wedding anniversary, we did it, we did it. All right, your mass, with everything, everything.

Mar. Did you remarry?

E9. Three times when I first got married, the silver anniversary and the golden anniversary

Mar. Very good, very beautiful. So do you think it is your character or have you been adapting to circumstances?

E9. Well, even if you don't want something, I have had to adapt, that is very natural because otherwise I would have said "today you stay here and I'm going ...." and no, I prefer to stay and let nothing happen and that's it, because one day when I left early, it happened [he goes to his son] that you came to call me running ...

CHILD. I dont know…

Mar. Something would happen.

E9. What did he want ... as soon as he knows I'm not here, he has to go pee or poop, something.

Mar. There is much written about the type of dependency that is established between the sick person and the caregiver….

E9. Right now I was in La Fe a month ago, it turns out that I got dizzy and I was returning, they gave me a bag and I returned, I had a terrible time, all because of the leg pain, which took me a pain that was dying, it had not happened to me never, I say it for this, because the very kind nurses all the truth. But what can I tell you, I was not like another ... they put me in some apartments that are meant to be a few hours, a time, three or four hours. And the other lady next door, who by the way, there are curtains eh, everyone has her apartment, come scream, come scream ... either it would not be right in the head or it could not bear what I can bear. The nurses used to do this to me [she gestures with her face], as if to say: "What neighbor do you have?" Well, that, I didn't scream far from it, I just said: "what a war I'm giving you so much to give back", they told me: "don't worry woman, don't worry" ... I was the other way around, suffering.

Mar. You do not like to work?

E9. I try not to give it [laughing], they tell me that if they have to do what I say, [laughs], well.

Mar. What do you plan to do with this situation more when you have to have surgery? Do you already know for sure that you are going to have surgery?

E9. No, I still have to go to the doctor ...

Mar. They have to do some test.

E9. If they still have to do tests and I have to go to D. Luis….

CHILD. To the street XXX

E9. I have to go to the bone one yet.

Mar. To the traumatologist

E9. To XXX Street. I was about to have an operation in XXX, but the good man who took me the last one screwed him up, that of the team, you take one today and tomorrow you take another, that's the worst for me. Then, the second he caught me, because the first one did an analysis of everything, X-rays, in short all the tests, he even did the metal allergy test, I told him: “if I wear bad earrings, I won't resist them. ", And he told me:" that's enough "and he made me do some tests at XXX 35'07 They told me that no [I was not allergic] that they could operate on me. Then he took another and when he saw me thick, the man told me that he was not operating on me, that at the moment he was not operating on me, that it was a back problem. So I did have to go to the "I'll say it if I can because I already lack a bit of clarity", let's say like this, to the office and the family doctor told me to go to rehabilitation. The rehab doctor gave me a girdle to wear and it went well.

Mar. Holds him.

E9. Yes, well, but the legs are the same. Now my back is better wearing that girdle… .All this, how much do I have to tell you huh.

Mar. There are so many things ... I was asking you before that if you had to have surgery, what was going to happen at home?

E9. Well, nothing will happen if God wants and the Virgin, nothing will happen because my daughter is there, there is my son, there is the lady who takes care of me who already knows all the tricks that I am bringing her up to date, I believe that nothing will happen and Besides, I am not so complaining about being in a clinic and in short, working hard. I'll settle for being alone for many hours or whatever.

Mar. You have already thought about it for the future, somehow you have it under control

E9. I have thought it all over and also God will provide, God will provide. I have friends who may stay with me, well, a lot of things.

Mar. I don't think I asked you because we haven't commented on it. Have you learned anything from taking care of your sister-in-law?

E9. What if I have learned?

Mar. Yes, as a person and then who has taught you to care? For example to click…. 37`33

E9. Well, to DJ? Look, I had a brother-in-law, God keep him in glory, he was a lot of DJing, he was a practitioner, I saw him because I have learned and also I already know that in the popis you have to look at the line of the popis where you click to know the site and also from I have punctured my children small. My husband held it between my legs and I kicked it, and now my husband, who needs it, is also prodding it. Because I really liked him.

Mar. So you have learned by watching, observing. Are you very observant?

E9. Much much

Mar. I ask you because your sister-in-law does not have wounds, ulcers that she must heal?

E9. No, well she has one on her heel, a little bit, but when she came to XXX the other day he told me what to do and that's it, I'm doing it to her.

Mar. Do you have problems treating that little sore?

E9. Not at all, not at all, everything suits me. The GP tells me that I am a nurse.

CHILD. The doctor also wants to take her as a nurse.

E9. In other words, what I tell you is quite a lot "you should have been a nurse, you haven't seen something yet and that's it" [the doctor tells him] I have that facility. A neighbor of mine, who has already died, told me: "XXX is that you see everything right away, you see everything right away."

Mar. She is very observant and learns right away

E9. For example, I am going to tell you something that is not relevant to this case. I was going to buy fish and bought some of these that are flat ...

Mar. Soles?

E9. Sole and I noticed how the fisherwoman cleaned it because at first I liked her to do it, but once I saw how she cleaned it I did it at home and my neighbor said: “Of course you are tremendous, if you have already learned Well now, make me mine ”and he made them.

Mar. In other words, you were carrying more and more work, eh, yours and the neighbor's. Come watch and observe and come load up on work ...

E9. [Laughing] I like, “that you have to cut meat”, because maybe I would too

Mar. Sure ...

E9. In other words, it is more than anything to be aware of things.

Mar. Y What have you learned on a personal level? If you have learned something, what does caring for your sister-in-law do for you?

E9. I don't know in what way

Mar. There are people who can say: "I have developed more patience", I was very active or very nervous and as a result of taking care of my sister-in-law ...

E9. Well, the nerves are always being nervous all day, it does not suit you, it does not suit you. Well, you do it with patience and nothing more. What happens is that there are times what I say, there are times that you can't, but those times happen….

Mar. And how has this been achieved? Over time, experience?

E9. Well, everything will be, a little bit of everything, because the truth is, I have the experience of caring. I have had my mother to take care of her, I have had an aunt and now I have my sister-in-law, in other words, I have taken care of myself like this… .behind one another. My mother also had to take 41'36 to the service and dress her, everything, that is, the experience has already come from afar and besides that, I have noticed things. And that I can tell you….

Mar. Now if you have surgery, they will have to take care of you ...

E9. Well, as little as possible.

Mar. Okay, but leave them a little bit too

E9. [Laughing] I don't want to give anyone a job, as long as I can do it, I don't give anyone a job. I had gallbladder surgery and just that day was my granddaughter's graduation. I couldn't go, you did, right? [He asks her son]

CHILD. Yes, yes [reluctantly]

E9. And my husband. I came home with two sisters “there are four of us in total”, two sisters who live here came, the other lives further away, they came and stayed. They gave me dinner and "E9 this or that" and I was so happy and so hot. I mean that I had to stay and be with them because it just so happened that the girl's graduation was that day, I mean that I came home from XXX and I was not there at all because I was pocha than pocha but I, nothing happens, the next day you're better [laughing]

Mar. Is the house comfortable for you, to move, to clean your sister-in-law?

E9. Yes, she has her bed that is raised from her back and feet and of course at night I lift her feet, which are almost always swollen, "these days they have not swollen so much" and I also raise the headboard a little and well. There are two bathrooms that seem not to but, if we are with her in one, the others can go to the other

CHILD. She [the aunt] carries a package

E9. She carries a package that she now sees ... I always pay attention to everything, the time. I am the friend of the hour, now I take him to pee, I prick him to….

Mar. Blood glucose, sugar?

E9. Yes, sugar, before, I pricked her every day but the doctor told me no, that with twice a week already enough and I just do it on Mondays and Fridays and now the time is approaching, so I am watch earring

**E10**

E10, he walks with difficulty, presents a significant kyphosis. In the double room is where she has placed her husband, in an articulated bed, next to it, there is a small bed, where E10 sleeps. The room is spacious, clean and airy, there is no furniture to impede the passage. Until that moment, only the moans and screams of XXX altered the atmosphere. She talks to him as if he could answer her, he tells him the reason for my visit, XXX is calm, quiet and seems to listen, he fills him with kisses and caresses and says several times: but how handsome you are….

Mar: E10 tell me what a normal day is like with her husband.

E10. Well, I never go out, only if ever. As this woman lives across the street, I tell her: "Come with me a little to buy" and my son who is unemployed, because I have one here and the other has his house upstairs. The other one has an accident and is down I don't know what will happen to him, what they will do to him, they have already done a lot of things to his left hand, he has an iron, total that his hand hurts and that, and this my son is unemployed and is here with me at home.

Mar. What has he got? Two children?

E10. I have two sons. I have had seven. Four girls and three boys, And I have died five. SILENCE Four girls and a boy have died. Here a girl and a boy and in Santa Cruz de la Múdela, who lived then, we have been here for many years, I came here with 30 years. And since my girls were dying, my brother-in-law who was here told me to come. Of course, my mother lived at first and my mother was not well and my sisters were all out of her I was always with her that I stayed with my mother until I got married. My mother could not come because she suffered from asthma and the terrain was not going well for her. My sister lived here, she came one day and she knew she was not doing well. She went to XXX with the other one. From XXX she went to my house and she already said that she did not want to leave her house. My brother-in-law told me but…. I wasn't going to leave my mother alone? I totally did not come. Then when my mother died I said "well, what am I doing here, my sisters who are doing their march and everything? Well, I'm leaving." I came with my sister XXX and my brother-in-law who was a railwayman and he brought me the furniture, he found me a house in the same place where she lived in XXX and I came. My husband then thank goodness that he had a job, my brother-in-law and my nephew worked on lamps and they put him there, he earned very little. At the time we were very, very bad, but in short, with time he fixed himself, he changed jobs, well, normal.

Mar. You have always worked at home

E10. In my house, in the field, well, I have worked a lot and here I have sewn that at home. Total that when I already had two children, that I had these two, I stopped sewing, raised them, took them to school and all that.

Mar. The lady is out, has she said that she is a neighbor?

E10. Yes, yes, she is the neighbor across the street who comes and as now that she is going to buy, I tell her "well she brings me this from the pharmacy" because I am very sick to go alone. I feel a little better in the morning because I wake up more rested but my head, the back of my neck, everything is that I have this porosis, my hands hurt, I cannot do a ferza to cook the pot, everything hurts and they told me that with what that I have, I always have to die with the pain, so I am wrong, you know, wrong and I 5'18 because to the woman I say "well come and take me to this, you take me to the other" and my children they stay here, if not, I tell her to go, "yes, I have to go to my daughter to get her out of the pharmacy, or I don't know what," she told me today as well.

Mar. Do you pay him any money or ...?

E10. Yes, I give him something from time to time.

Mar. I am asking you because I am interested in knowing if you have any kind of help.

E10. Well yes, they gave us help but they took it away from us. They gave me…. How much was it? For my husband financial help, as I helped him, I want to touch him, and God willing that nothing happens to me before him, because I ... look at you, I have loved my husband like, like nobody else has. dear to your husband, well, because I have loved him very much, but now look at you, now, I love him more. With all this, I tell him from the heart, I go to bed "then you will go to our room" we have two beds there, I bought him a hospital bed I said: "whatever God wants" and I sleep with him and every Every time he coughs or does something… .that many times, I am giving him that medicine, some pills to start with and that, I take some wipes or some gauze good of everything to be able to remove it, sometimes he swallows it, others I take it out ... And so and so I go pulling. I do not want to stop attending my husband.

Mar E10 I understand that he has said that now you love him more.

E10. Oh, I do, I still love him more, look, I love him very much, I talk to him a lot, I tell him when we get married, that we are husband and wife….

Mar. Does it remind you of things?

E10. He giggles like that. Before he used to tell me "XXX" he called me well and everything ... not now. There are times when And I say "oh he told me E10", because with his tongue it seems, well he doesn't even stick his tongue out, I don't know what's wrong with his throat, he has some lumps there. He has always suffered from angina, but, I went to that throat when he could go in a cart and all that ...

Mar. I saw that XXX is bedridden, does he not get up?

E10. No, no, before yes, we brought it with the cart here to the table, but now nothing, nothing ...

Sea Do you speak?

E10. No no, just (… ..) "My son, I don't know what you're saying, but I love you very much, eat because you can't be without eating." Well, I already tell you that I say a lot of nonsense that then I say "anyone who hears me will say that woman is like a goat"

Mar. No, because you communicate with him

E10. I do, because I think he wants to tell me something or with a little smile and I, when he does that to me, I ... there is a day that I don't know what he told me, something like he understood a "yes or what ... something" oops, it gave me a joy…. You do not know the joy that he gave me [gets emotional and cries]

Mar Claro, because you have understood that XXX understands ...

E10. Yes, he knows that I am his wife, what happens that he cannot tell me, he does not speak and he has moments that, like Alzheimer's, what he has. There are times that if he will realize what I say but other times well ... it is clear that he does not. But just in case he hears me or something, I say many things to him, "oh my husband" [with longing]. It has been very good for me and I for him and I love him very much.

Mar. Their relationship has been very good… [She is very excited]

E10. Yes, very good, and where he has gone, I have gone, where I have gone, he has gone. We were going to XXXX which was a sister. I would go there with him and he would take the car, so we bought a buggy and we would go and spend every summer there, he worked and everything, but, he could no longer drive, and one day I told him “XXX, do you know where we are going? and all that?" He said yes, but the car did not handle it well.

Mar. You noticed that something was happening.

E10. Of course, he no longer had the car well and of course, here in XXX, they saw him from the head and I said to the doctor, what was his name? , oh ... I have the letter and everything that my husband had, well, he told me that if he did not get it better than not 10,18 and no longer…. and he wanted to get his license. On the same day of his 13th birthday, we went to that card…. to renew it and I knew they weren't going to give it to them, but I say "so that you are satisfied, we are going to go" and I don't get it right, I say "if I knew it." Then my daughter-in-law came, who have now argued and…. I have it here [his son]. I have two grandchildren, one small has it, another has gone with her mother-in-law, with her mother or her brother, I don't know, to spend a few months… so look, I don't know.

Mar. E10 Since when is XXX in this situation?

E10. Oh, well, many years ago, when did the euros start? ...

Mar. As it is now, no, it has been little by little…. But, in this situation?

E10. Before I went to the east site of….

Mar. Rehabilitation?

E10. No, no, they were coming for him and that, what is his name? It is that I do not remember ... a house of those that ...

Mar. Don't worry E10,

E10. A house of those that took it in the morning and brought it in the afternoon ...

Mar. A day center….

E10. That, a day center and it has been like this for a long time, but already on a vacation that they threw in the center ... I was already scared because since we do not have an elevator, when I went down I said "take it well", I was going with the garrotilla and I was holding the belt well from behind [XXX is heard shouting] and I was also holding onto the railing in case I fell, he held on and that, but I was scared, then we weren't going well anymore…. I put the club in the middle, in short, I said "we are both going to fall" and we already said "well, look, I don't dare anymore" they spent the holidays and I told them that I was not going to go, because with the stairs I no longer I could and I alone ... so I did not have my children here, I was alone and I did not, I could not.

Mar. And how do you do now? How do you lift it? Because you do it all

E10. Yes, but I tell this woman, since she lives across the street, she comes three times a day and she helps me ...

Mar. When you are going to move it, change it, you notify the neighbor

E10. If she comes to her, what are we going to do?

Mar. Do children help you?

E10. Well, they help me, for example, when we go shopping or to give me a little air that I go very badly [he refers to his mobility, to walk] because, if we do not carry a cart I will hold on to it, but if it carries The cart she, because with one of those handheld does not fit, because I charge water for the week ……. This Saturday I have not gone to buy, so…. cast

Mar. So, when you get out of it, you do it with her

E10. Yes, we buy ……

Mar. And with her husband, her son stays.

E10. That, my son. Of course, before the woman would go and I would stay, of course, because my children were not here and now my son remains, the other one lives higher up.

Mar. Are the grandchildren coming?

E10. Sometimes. They have been here weekends, but now, is that the big one, I do not know that he lacked the card that they had not done, I do not know and totally that they have not caught him at school this year and of course, how is he going to be the boy without this one or nothing, with which he has gone to the town of my mother-in-law that there is a school… [The doorbell rings] I'm going to open. This is the lady, who is coming.

Mar. We talked about the help of the grandchildren, children 15'19

E10. Not before, because since I did everything by myself until na always changing it and to and before we had a bed, which I have in this room, it is my double bed and now my son is there and I have taken my own. I took her little room for myself and bought one of those from the hospital for my husband, and that.

Mar. E10, do you move around the house well? Are you comfortable doing things well?

E10. I get tired, I get tired

Mar. I'm not saying it because it gets tired, but because of the space, is it comfortable? Can you move well to attend to her husband?

E10. I do, yes, oops, with all my soul. I place everything, him and I like to do things for him.

Mar. She has had to fix the house, change furniture to be more comfortable….

E10. Yes, yes, my son, like him, has those things [points to the computer] look at it.

Mar. Yes the computer.

E10. I get along very well with my husband. I make him eat, I feed him, I make my food. He does not snot and what happens is that they go there [points to his throat] because we were in XXX for that once. They put on and took out snot and stuff. What goes around is always constipated

Mar. Did it seem to me that you were telling this lady that she had a sore?

E10. Yes, yes, in the east side [he points to the right side] he has it that I put a gauze on him and it gets stained with blood, from that saying that he made a lot of blood, no, but he has it raw

Mar. Are the nurses coming?

E10. Well yes, look at XXX wine and he takes our blood pressure, I was 16, I was quite a bit, he had it better, he gave us the vaccine.

Mar. Now, will you have to tell him about the sore?

E10. Well look, this trip that came, I didn't say anything to him, I didn't remember the sore. And that he has another in this side [points to the opposite side], you know, but he already has that very well, he sometimes turns colored, sometimes not and of course, he puts on it even though he has no na, he puts a gas and powder And in this one, well, he throws it, what is he going to put? Well, powders so that he holds a little more to this one so that the gauze does not stick so much.

Mar And who has taught you to heal?

E10. Oh, my mother, we wash very well wash yourselves, and we change him very often and all that

Mar. But had he done it before?

E10. No, I alone, I never. My poor mother, she was a little bad there, she was there for 15 days, she also stopped talking, she did not speak to me, she listened yes, pBut he wasn't talking to me.

Mar. So this is the first time you've done these things?

E10. Yes, yes, I do and I want to do everything for my husband and what if the wife helps me? I do not withdraw from it, I can not be for it is that I can not be for 19'18

Mar. Have you told me that you go out from time to time to go shopping?

E10. But if the woman goes to the pharmacy, she comes with the diapers, the medicines and all that because she brings it to me because for her to stay, then I stay with my husband, the better. Yes because that way if she has to get out of it, she buys for her and she manages too.

Mar. You said at the beginning "I think I love my husband even more". What feelings do you have when your husband is in this situation? How do you live this situation? Because easy, I don't think it is.

E10. No, it's not easy because you just look at feeding him I have to suffer. I am making food in the kitchen and I put myself on the bench like this [he does the posture] I like this for a long time so I can't stay because I suffer, I lean on the marble and start peeling the potatoes whatever, I fix it and then If I start cooking then I have to hold on, I don't reach up because I used to, my mother if as I say if I get to know how I am going to put the hangers so big, so high and all, because I have been tall, but i'm shrinking

Mar. Aging what it does

E10. Oh what he does and the pains, the pains.

Mar. I mean, her husband is wrong and so are you

E10. I with my husband giving him that [food] and I am suffering more and I tell him "oh my son, hurry up" oh what a pain and many times he looks at me and it gets like this ... there is XXX come pa ca and he looks at me because I can't stand it , finally I change the position 21'10 and I do everything eh, I already tell you

Mar. You are looking for the postures that allow you to do the activities

E10. Look, I have painted my house, I have washed my curtains myself, what am I going to say? I have everything, everything, everything

Mar. But now he can't do that E10

E10. No, that's not why I have the woman who washes the curtains for me, I put the things on her, I give them to her but she puts them on me [she hangs them] But before, my mother walks, but if I I have done my sister and my poor husband, my niece says “oh aunt, what I remember about the uncle who always came to do me I don't know what, I don't know how many” and I also put the curtains on my sister. I have helped my sisters here and in the village.

Mar. Are your sisters living?

E10. No, they have already passed away, I have my niece who lives nearby.

Mar. And what about her husband's family?

E10. I have a sister-in-law who is the same as him, she lives in a nearby town [the husband can be heard screaming] and she has a brother near here and he comes to see him, that's what the poor man says, he does nothing, and he says and what should I do? ”, my son, what are we going to do?

Mar. The situation is difficult. E10, do you receive a pension

E10. Oh sure, if she didn't charge it what? a disaster, it is the only thing, of course and I already tell you, look at what they were giving me because they took it away from me, but then I have seen in the book and they have given me something € 300 or so and I say, little help is also good.

Mar. What he says they gave him, would it be because of the dependency law?

E10. Yes, it is that we walk with that, we requested it and they gave me a very good help because it is that the wage does not finish me, no, no, no, with a thousand euros and now the medicines that I look at, I have a lot of papers when do I Will they pay everything that I am paying? hey and the medicines that I'm paying for he and me

Mar. You will have to ask at the pharmacy.

E10. Of course, the one from the pharmacy is the one who gives me the papers, but he told me “look, they will have to give you, I don't know what, you have to ask the doctor, I told the doctor and“ go to the counter and now They will tell you “I went that day with the woman, we told them and they told me that they did not know when they would give it to me but that it was 50%, but they have not given anything. Others say that maybe in the rent or I do not know.

Mar. Anyway you don't lose the papers because the moment they start to pay back the money we all find out and what you will need is those papers.

E10. Of course, I have the papers that they give me at the pharmacy, those that enter the pharmacy [social security], those that do not enter do not. That I am also paying for things.

Mar. Because you buy things that do not enter into social security, does the doctor give them to you?

E10. Look, the doctor does not give me the pills that I give him.

Mar. And why do you buy them?

Mar. E10 Since when is XXX in this situation?

E10. Oh, well, many years ago, when did the euros start? ...

Mar. As it is now, no, it has been little by little…. But, in this situation?

E10. Before I went to the east site of….

Mar. Rehabilitation?

E10. No, no, they were coming for him and that, what is his name? It is that I do not remember ... a house of those that ...

Mar. Don't worry E10,

E10. A house of those that took it in the morning and brought it in the afternoon ...

Mar. A day center….

E10. That, a day center and it has been like this for a long time, but already on a vacation that they threw in the center ... I was already scared because since we do not have an elevator, when I went down I said "take it well", I was going with the garrotilla and I was holding the belt well from behind [XXX is heard shouting] and I was also holding onto the railing in case I fell, he held on and that, but I was scared, then we weren't going well anymore…. I put the club in the middle, in short, I said "we are both going to fall" and we already said "well, look, I don't dare anymore" they spent the holidays and I told them that I was not going to go, because with the stairs I no longer I could and I alone ... so I did not have my children here, I was alone and I did not, I could not.

Mar. And how do you do now? How do you lift it? Because you do it all

E10. Yes, but I tell this woman, since she lives across the street, she comes three times a day and she helps me ...

Mar. When you are going to move it, change it, you notify the neighbor

E10. If she comes to her, what are we going to do?

Mar. Do children help you?

E10. Well, they help me, for example, when we go shopping or to give me a little air that I go very badly [he refers to his mobility, to walk] because, if we do not carry a cart I will hold on to it, but if it carries The cart she, because with one of those handheld does not fit, because I charge water for the week ……. This Saturday I have not gone to buy, so…. cast

Mar. So, when you get out of it, you do it with her

E10. Yes, we buy ……

Mar. And with her husband, her son stays.

E10. That, my son. Of course, before the woman would go and I would stay, of course, because my children were not here and now my son remains, the other one lives higher up.

Mar. Are the grandchildren coming?

E10. Sometimes. They have been here weekends, but now, is that the big one, I do not know that he lacked the card that they had not done, I do not know and totally that they have not caught him at school this year and of course, how is he going to be the boy without this one or nothing, with which he has gone to the town of my mother-in-law that there is a school… [The doorbell rings] I'm going to open. This is the lady, who is coming.

Mar. We talked about the help of the grandchildren, children 15'19

E10. Not before, because since I did everything by myself until na always changing it and to and before we had a bed, which I have in this room, it is my double bed and now my son is there and I have taken my own. I took her little room for myself and bought one of those from the hospital for my husband, and that.

Mar. E10, do you move around the house well? Are you comfortable doing things well?

E10. I get tired, I get tired

Mar. I'm not saying it because it gets tired, but because of the space, is it comfortable? Can you move well to attend to her husband?

E10. I do, yes, oops, with all my soul. I place everything, him and I like to do things for him.

Mar. She has had to fix the house, change furniture to be more comfortable….

E10. Yes, yes, my son, like him, has those things [points to the computer] look at it.

Mar. Yes the computer.

E10. I get along very well with my husband. I make him eat, I feed him, I make my food. He does not snot and what happens is that they go there [points to his throat] because we were in XXX for that once. They put on and took out snot and stuff. What goes around is always constipated

Mar. Did it seem to me that you were telling this lady that she had a sore?

E10. Yes, yes, in the east side [he points to the right side] he has it that I put a gauze on him and it gets stained with blood, from that saying that he made a lot of blood, no, but he has it raw

Mar. Are the nurses coming?

E10. Well yes, look at XXX wine and he takes our blood pressure, I was 16, I was quite a bit, he had it better, he gave us the vaccine.

Mar. Now, will you have to tell him about the sore?

E10. Well look, this trip that came, I didn't say anything to him, I didn't remember the sore. And that he has another in this side [points to the opposite side], you know, but he already has that very well, he sometimes turns colored, sometimes not and of course, he puts on it even though he has no na, he puts a gas and powder And in this one, well, he throws it, what is he going to put? Well, powders so that he holds a little more to this one so that the gauze does not stick so much.

Mar And who has taught you to heal?

E10. Oh, my mother, we wash very well wash yourselves, and we change him very often and all that

Mar. But had he done it before?

E10. No, I alone, I never. My poor mother, she was a little bad there, she was there for 15 days, she also stopped talking, she did not speak to me, she listened yes, pBut he wasn't talking to me.

Mar. So this is the first time you've done these things?

E10. Yes, yes, I do and I want to do everything for my husband and what if the wife helps me? I do not withdraw from it, I can not be for it is that I can not be for 19'18

Mar. Have you told me that you go out from time to time to go shopping?

E10. But if the woman goes to the pharmacy, she comes with the diapers, the medicines and all that because she brings it to me because for her to stay, then I stay with my husband, the better. Yes because that way if she has to get out of it, she buys for her and she manages too.

Mar. You said at the beginning "I think I love my husband even more". What feelings do you have when your husband is in this situation? How do you live this situation? Because easy, I don't think it is.

E10. No, it's not easy because you just look at feeding him I have to suffer. I am making food in the kitchen and I put myself on the bench like this [he does the posture] I like this for a long time so I can't stay because I suffer, I lean on the marble and start peeling the potatoes whatever, I fix it and then If I start cooking then I have to hold on, I don't reach up because I used to, my mother if as I say if I get to know how I am going to put the hangers so big, so high and all, because I have been tall, but i'm shrinking

Mar. Aging what it does

E10. Oh what he does and the pains, the pains.

Mar. I mean, her husband is wrong and so are you

E10. I with my husband giving him that [food] and I am suffering more and I tell him "oh my son, hurry up" oh what a pain and many times he looks at me and it gets like this ... there is XXX come pa ca and he looks at me because I can't stand it , finally I change the position 21'10 and I do everything eh, I already tell you

Mar. You are looking for the postures that allow you to do the activities

E10. Look, I have painted my house, I have washed my curtains myself, what am I going to say? I have everything, everything, everything

Mar. But now he can't do that E10

E10. No, that's not why I have the woman who washes the curtains for me, I put the things on her, I give them to her but she puts them on me [she hangs them] But before, my mother walks, but if I I have done my sister and my poor husband, my niece says “oh aunt, what I remember about the uncle who always came to do me I don't know what, I don't know how many” and I also put the curtains on my sister. I have helped my sisters here and in the village.

Mar. Are your sisters living?

E10. No, they have already passed away, I have my niece who lives nearby.

Mar. And what about her husband's family?

E10. I have a sister-in-law who is the same as him, she lives in a nearby town [the husband can be heard screaming] and she has a brother near here and he comes to see him, that's what the poor man says, he does nothing, and he says and what should I do? ”, my son, what are we going to do?

Mar. The situation is difficult. E10, do you receive a pension

E10. Oh sure, if she didn't charge it what? a disaster, it is the only thing, of course and I already tell you, look at what they were giving me because they took it away from me, but then I have seen in the book and they have given me something € 300 or so and I say, little help is also good.

Mar. What he says they gave him, would it be because of the dependency law?

E10. Yes, it is that we walk with that, we requested it and they gave me a very good help because it is that the wage does not finish me, no, no, no, with a thousand euros and now the medicines that I look at, I have a lot of papers when do I Will they pay everything that I am paying? hey and the medicines that I'm paying for he and me

Mar. You will have to ask at the pharmacy.

E10. Of course, the one from the pharmacy is the one who gives me the papers, but he told me “look, they will have to give you, I don't know what, you have to ask the doctor, I told the doctor and“ go to the counter and now They will tell you “I went that day with the woman, we told them and they told me that they did not know when they would give it to me but that it was 50%, but they have not given anything. Others say that maybe in the rent or I do not know.

Mar. Anyway you don't lose the papers because the moment they start to pay back the money we all find out and what you will need is those papers.

E10. Of course, I have the papers that they give me at the pharmacy, those that enter the pharmacy [social security], those that do not enter do not. That I am also paying for things.

Mar. Because you buy things that do not enter into social security, does the doctor give them to you?

E10. Look, the doctor does not give me the pills that I give him.

Mar. And why do you buy them?

E10. I give him one that I don't forget for a day, one, every midday I give it to him

Mar. But why? Do you think they are going well for you?

E10. The thing is that they sent it to me there [in the hospital, a long time ago] in XXX and this doctor is giving it to me too, but since she says that that does not enter into that thing, because I am not going to take it off before I take it off I eat that to him.

Mar. The doctor knows that you give her that pill

E10. Yes, they don't give me a prescription for that and I have to pay for it myself, the diapers, everything

Mar. E10 What difficulties do you have, normally taking care of your husband? Or What things do you find it difficult to do the most?

E10. Well, I can't lift it like before, I can't move it by myself. With the woman we already managed, bad but. Look at you, it turns out that you do not get dirty well, you always get constipated and when you go without constipation there are times when it is soft, it is that you don't throw it at me! Nothing, he doesn't throw it at me and I put on a glove and I take it off myself.

Mar. Very good

E10. When he was in XXX and he didn't poop, they gave him a lot of things and when he came home, my goodness, what I have done and I every day, well, day yes, day not because there are days that, today it has already been dirty, today it has been like shavings today I have taken it out too. I have that patience that I put on the glove, I dip it in oil that I have in the kitchen I have a covered cup, I wet these fingers [points to the fingers] and I take out

Mar. The hardest thing is to move it.

E10. I can't by myself, if I could move it as before because I used to move it, but it is heavy and thinner

Mar. her husband weighs and the years also pass for you, or it is not that too.

E10. And this neck, maybe this woman tells me "I have a pain" and I tell her "Well, look, I can't say that this or that hurts because everything hurts" my arm hurts, my hands are They sleep, they give me cramps at night in bed. Sometimes when I get up, oh, oh, I can't move until I feel good, and getting dressed is hard for me.

Mar. That's why I tell you that the situation of her husband is one thing but you are another.

E10. Hopefully I can do my thing that nobody does to me "oh my God that nothing happens to me before my husband" I really take care of my husband while he lives and I when my husband leaves if he leaves or I'm going, I will come back or I will come the next day 30'24. If I had a daughter like my mother had [she starts crying], I love my mother very much [there is a lot of pain, emotion] and I remember a lot, because my poor mother said "oh E10 always cleaning always doing things" and then work I went to work, my mother the only thing she did was her bed because fatigue did not allow her to do anything. Look, I would go to work, I would get up at five in the morning and before leaving I would wash my house, the kitchen, everything, there in the town, then I would go to work, just to tell you that I washed the threshold and in the winter I froze right away and my mother would scold me “oh my daughter, how much pain”, I remember a lot, [she cries with regret], she always told me, but what was I going to do, I like to do things. If the woman sees me do it and if she does not let me, I am suffering because I cannot do it.

Mar. You've been working your whole life.

E10. Lifetime

Mar. And dedicating his life to others ...

E10. Working, reaping, because before we had and also worked in the field, we had a garden, we already sold it and we had a field, before we were fine, but when the war, they took away our galley, they took away our land, many things and we were clear with less, we had profits when my father died, the mules were sold, only my brother remained and then there was no more for him who was married and there was nothing left because they sold. My mother was left without na, na na, my mother without a penny and my older brother says “you stay here at home with mother and you keep her, you know? expenses, paying for things and supporting my mother and I told her "I don't support mother, I'm here with mother and my mother is there"

Mar. E10 she is a very strong woman.

E10. So when my mother died, the same day my mother died, I left, I went to my mother-in-law's house and then I went to a wedge's house and in the dining room I put my room, then I bought an apartment, a little house there with another girl and I got in there until I came here. But I, I have suffered a lot because I with my four girls who have died that one died of nine months, another of seven, the one of seven was from here, another of four, another of four and something I no longer I remember, when my mother died I was pregnant and I went to XXX because I say: if my mother does not have and my father-in-law then had goats and sheep and that but she was not going to take care of me, well I say: I am going to XXX so that they take care of me and to see why the girls die and they told me that the girls were born very well, but the time they were sucking very well 34 but then They got angry or wanted to suck, or the kettle, they did not want even the spoon that poured anything, nothing and the doctor told me "you give so much serum and I don't know what much", but I say "if you do not drink any milk and he throws it at me ”. They couldn't live, they couldn't live and when the first one died there, in the town, I told my mother “mother, mother today yes”, the one that I brought from XXX “today the girl has taken me very well "And my poor mother said" yes, yes, yes "and the practitioner came because as they pricked him and I said" Mr. XXX, look at the girl has taken it well "because she says" oh well yes "and my poor thing is that he was already swallowing, he was dying and…. all of them have died in my arms 35'13 The boy here in my arms, the girl who took her as she was here, I took her there on the San XXX bridge there was a….

Mar. As it was called, a dispensary….

E10. Yes, yes and my sister like I lived there with my sister

Mar. The first aid house I think was called

E10. That, the first aid house, and then we went “oh look XXX, my girl how is she” and she says “oh let's go there, let's go”, when we arrive they say “look, take her home, it can't be, because if she dies here, you can't take her away ”and…. All of them in my arms have died and my child, the boy, look at you, he grieved more to die, with eyes that were putting on him, look, I do not know, I do not know how my children have died, I do not I know [gets emotional and cries]

Mar. But, E10, it is very strong, everything that has happened and is still taking care of

E10. They told me “oh my daughter I didn't bring any more children”, the girls, my friends 36'12 and I say “well daughter, I ache in my heart because I suffer a lot, look, when I pick up the crib, pick up the rags, pick up everything and wing” , but I, is that I wanted to have children and I said to the doctor "look, if it consists of me, I will not bring more" and he said "no ma'am", they did the HR "for you it does not consist" because we both they made…..

Mar. Yes, they were things that happened outside of you.

E10. Yes and thank goodness that I achieve these two, two tiacos, two tiacos….

Mar. See what life is….

E10. I have suffered a lot… [she relaxes].

Mar. E10 Why do you take care of her husband? What is the reason?

E10. Well, because I have the heart and the energy pa, pa that …… that ……. What do I know …… that I love him, that I love him [cries]

Mar. For love.

E10. That, out of love [cries] like my children who loved them and I love them and I love them all, "oh my God, don't let it happen to them that they are already men who are already men ..." [She continues crying with regret, we stop and when relax].

Mar. E10, Do You Feel Like You?

E10. Not

Mar. Do you feel recognized?

E10. No, I am saddened, as they are men, they go out and that, you know, I….

Mar. Are you being cared for?

E10. Me?… .No, who goes, they leave and… when the grandchildren are here, my son takes care of feeding them, because they are very bad to eat, the little one.

Mar. But, from you, from you.

E10. From my?

Mar. You notice that they take care of her, that they care about you, that they love her.

E10. Well, loving me, they love me.

Mar. Do they pamper you from time to time?

E10. Hey, they give me kisses and stuff, but what am I going to do ...

Mar. You would need more ... [Laughs of both] Yes or no?

E10. Yes, but I eat more with my husband [smiles] than with them [laughs and shakes his head] is the truth, I am here and I am with my husband, I am with food, with that, with the other and I spend the day like this, then the weeks, I say “oh but if we are already in this day, we are already tomorrow…. to Friday and so on ”

Mar. Time passes quickly.

E10. Does it happen? It passes me right away, the days right away, my God.

Mar. What things have you discovered, taking care of your husband?

E10. Well, what do I know ... it seems that I deny more sometimes [he laughs], oh, what do I know [he is sad again], what do I know, if I say something [to the children], then it weighs on me to say "you are going "And then I say" so I say na "and all those things, but they are men and of course, they have to go out and I ..." Mommy I'm leaving "," good "and so we go, oh my Lord, let it be what God wants .

Mar. Are you happy now?

E10. Oh, I'm happy, I'm not happy….

Mar. Happy? How do you feel now?

[It takes time to answer]

E10. [Sighs deeply] Oh well, how can I tell him, because I have happy times when I'm like this with my husband that I'm saying things to him, I'm enjoying that ...

Mar Even though at that moment you feel bad?

E10. Yeah, even if it's wrong, then maybe I'm here and I start crying, you know?

Mar. When you have those moments of being sad or crying, what do you do? How do you get over those moments?

E10. Well, it saddens me, then my children… they tell me… the woman is “there are some movies”. My son was the first to tell me “on the channel (…), there are some movies in the afternoon,” and you see, I'm watching the movie and I get up here and stand up, I can't be there either because I can. I have to do things, I go to what I have to do ...

Mar. Sewing, or other things, distracts him.

E10. Ah yes I look at you, right now I have a shirt of my son that has the neck like that a little bit of a corridor and I have unsewn it, I have it basted to sew it to the machine that I have in that room that is a storage room, well, I I also entertain myself in that, in sewing,… ..

Mar. Does it relax you?

E10. Yes, yes, I like to clean [the husband yells] I can't be around.

Mar. Do you notice that sometimes you have to stop or that you have to relax a little bit?

E10. That is what the woman tells me "you have to be relaxed" the woman tells me. Many times I sit there, but, I am like this and I get up right away and I am better distracted by doing things. Look, I'm making the food and so I have the food that is already on the fire, if I have a pot, that's it, I've already scrubbed it, I scrub it and I leave it ready. Then I'll go, it's already dry…. . Well, I clean it and keep it to myself and so, when I start to eat there are times that I have nothing left but my plate because when I have washed him I will leave everything except mine [silence]

Mar. Do you sleep well? Rest well?

E10. Well, the first dream I do very well, there are times that it is good, but other times, I wake up and I am…. [He gets sad] well, I wake up and I have a hard time falling asleep and when there are bad nights, because there are nights that uh ... many nights, many [XXX continues screaming], look at how you are, as long as you start with a cough ... that's the worst ... I tell him: "boy, it's nighttime" and he seems to hear me and shut up but There are times when I tell him whatever it is and no, nothing at all, I get up, give him water, other times I give him juice and so [he gets sad] ……

Mar. You are testing to see how he reacts.

E10. Yes, yes, I give him breakfast, juice and water, lunch, dessert, others I give him yogurt, others I put fruit, I crush it, which by the way when I crush I make for him and for me too because I crush a lot, a pear , an apple, a banana, well we both eat it, other times I say "well today I'm not going to do more than for him" look, the bananas I don't have anymore I've run out and things that, tomorrow maybe we'll have to go to buy, tomorrow or happen, so… .44`33

Mar. E10 I think he has commented on the questions that may interest me. Does he want to tell me something else that we have not discussed?

E10. What else can I tell you, my daughter, if I have already told you about my youth that my youth I have worked a lot because when we have had to work in my house and someone else knows you and when I have not worked in my house, the house And strangers, I have gone to the hills of the Virgen de XXX which is from Santa Cruz de XXX, there are some hills that have pine trees, well those that have dried up or those that dried up because we were going to make holes with the hoe to slopes like this we would walk from my house and my husband, who was dating then [his son enters, without saying anything and scares us] because he was watering with the backpack they carried and to the hill, then I was going to make the holes eh, others would go planting the pines and burying them and others making holes, I was making holes and when we got together my husband was going down the road and we got together, we were walking to the entrance of the town where he lived and I lived more E10nte by the Vía la Plata, so….

**E11**

E11. Because they have to charge me for the medicines that they charge me for all

Mar. With the new law ...

E11. The new law, listen is that I am chronic, look at you, I have a pharmacy here [all the drugs are in a visible cabinet] and now I am paying a lot in medicine.

Mar. This situation is creating many problems

E11. It was created by the socialist gentlemen. If you are a socialist, don't be too bad, but they are blaming the government and thanks to the government the scoundrel and disorder and disaster that existed in Spain are being discovered, they were going to sink it. You know that XXX, the socialists, as they left Spain.

Mar. How old are you E11?

E11. How many do I have?

Mar. Man if he tells me like that

E11. No, but I know almost how old you are.

Mar. How many do I have?

E11. About 54 or 55

Mar. Well, I'm 57

E11. So….

Mar. Well there is not so much from 54 to 57

E11. How many?

Mar. Eighty something

E11. Eighty something .... I don't know

Mar. Well, 83.

E11. I'm 84. You don't hit bad shots. We are not stupid.

Mar. We have not been wrong much. E11, tell me a normal day with XXX, what it does

E11. The march that I take is the following one, the poor thing, [goes to the assistant] XXX, if you want to sit down, this girl, this lady is a nurse comes to do a voluntary interview with me and she wants us to tell her every day, because this girl takes She has already been with us for three years here in my house and finally she already knows everything about the house.

[XXX from her bedroom asks if she can get up now]

E11. XXX you can hear it if you want. She takes everything and very well [he refers to the maid] Besides, I have another girl from the town hall who comes to clean me and shop.

Mar. Is the person from the town hall a help, a resource for the dependency law?

E11. Yes. I pay this one.

Mar. What is this person doing?

E11. Well, look at you. Right now he is with my wife, he helps her, takes out the urinals, the house, makes us breakfast and then makes dinner and if we have to go buy something, then she goes.

Mar. And you E11 What do you do?

E11. I have seen that I go with crutches, my wife with a walker. We go downstairs to the bar, have a coffee or go upstairs. Then we come and like this, how can I live.

Mar. How long has XXX been in this situation?

E11. Well, look, it's been years now, because she had a little thrombosis on the left side and since then, that and now it's going well, now she's with the walker. I had to buy a wheelchair because I was in pulmonology with the doctor (….) I went and he did not take me, another woman took me and they do not know, in a catheter that I had he put the urbason immobile. The next morning he comes and says he had caught a virus and I told him "look at me, the urbason lady does not suit me" I already quarreled with (....) It takes me 10 or 11 years, do not believe that I started yesterday and they tell me that I have caught a virus, I tell her “ma'am, I can disgrace you because the lawyer does not cost me anything” I told her “the lawyer that I have who has a law firm of tinkers that. . ”He became a notary public and in the street (….) As he did not have the socialist meat hanging in his mouth, they did not approve him and they told him to come back again and he said [he cuts the sleeves] for your father I am going to come back, he has a lot of self-esteem and then what did he do? I take a right coat and I set up a firm with another and the two of them are there and well…. The woman is a cashier and she knows that she is going to know you. I told her "look, lady, it's not hard for me ... I could ruin you" but I know that if I now denounce that woman

[His wife appears]

Mar y XXX Complimentary greetings.

[Follow E11]

E11. And nothing, he ignored me but I, so many things have happened to me in my life, I have already had two prostate operations and another that they have done there, which was not a new XXX prostate that I put the doctor (….) the ones I know, if you weren't ashamed to have me for a month with a probe placed on the 20th and a month later, it wasn't a prostate that I had. I had gotten a tiny lump, a small 8'56 tumor in the urine pocket and of course when they are going to operate the doctor says “you don't have a prostate”, rather a beginning kid “he wants me to operate it” I said “if I want to you practice today. " He sees me, calls the doctor and says "here is something that is not a prostate, a prostate does not have" and then they saw a small tumor and then they operated on me. But with the spasms and what I suffered more than all the time I've been in XXX because I know XXX better than you, I've been through everything eh and, I have no complaints

Mar. E11 from what you have told me, you go out

E11. We can go out but look at you, [she points to her crutches] and with the cart [XXX's walker] I also have a chair to carry her, this girl pushes her.

Mar. Of course you can't.

E11. But I have another electric that I buy [laughs] for when I have to go long, I better exercise but when I suffered from muscular osteoarthritis at every step, to the ground. What hice, stay crippled and abandon myself. I went to the emergency room later and if I tell you the story, my friends would tell me, XXX if one day you notice blood, go quickly, you'll die. Of course, I lost blood, I went to XXX and a certain one was a beginner (...) he didn't put blood on me or anything and after a while I was there I notice [makes a noise] I was like dead from 6 in the morning until 9 in the morning in a coma and then, myself [makes noise] and reacted. I didn't have this girl then, my wife called a niece and I gave that boy the biggest scare of his life because he knew he should put blood on me and since he didn't put me I had a drop in blood pressure and of course the poor thing [ his wife] passed her thing.

Mar. Do you have children?

Cristina and E11. No, either she is not worth it or I am not worth it or neither of us.

Mar. Now, what's done is done.

E11. [Addressing her wife] Have you noticed how young this woman is? Are you married?

Mar. Yes

E11. And do they have children?

Mar. A daughter.

E11. XXX, this lady is from your time and she see how she is preserved

Vero. Not of my time.

E11. No, you are older.

Vero. I do

E11. You older or her.

Vero. Not me.

E11. How does she know?

Vero. Ah

E11. How does she know that she is older?

Vero. Because if.

E11. But how old is this lady? XXX, she is young, she also does not know how old she is, I take care that she cheats.

Mar. How old are you?

E11. Few

Mar. Few? Well I will have a few more

E11. Less

Mar. Well less, E11, I am not going to argue now for years

E11. If we argue, we don't argue because we already understand each other.

Mar. But that will be you with XXX that already understand each other.

E11. You say that we understand each other, every day less [XXX laughs]

Mar. I thought he was going to say that they no longer argued, because they know each other so much.

E11. Do you argue with her husband or not?

Mar. Man sometime, yes, of course, if not that boring [Laughter]

E11. Men are tired, what if?

Mar. There is everything

E11. Well, ma'am, tell me more things.

Mar. No, not if you have to tell me. He has told me that he has help, which is what I am interested in knowing ...

E11. You help but paying me

Mar. Well of all, that has already been clear.

E11. Because I got a place for this neighborhood and in the social one I became close friends with (….) I don't know if you know that she is a councilor and has come to XXX to see me when I have been there and (….) When this happened to me He told me "check out the application." I applied and they called me right away because they are very happy with me and the work I did in the neighborhood. Then I have behaved very well for the time that I have been and this was abandoned that we called it "XXX" because the place where the club is on the street of (….) That is called XXX. I look at the plane (….) And it says “XXX” which is in (…) 19. More things, miss

Mar. How do you handle this situation?

E11. How do I handle it right now? I always carry it well

Mar. Well over the years, because you will have noticed the passage of time.

E11. How can I not notice? Who does not notice it, because now I am a shadow.

Mar. What happened to your legs? How long?

E11. How many years ago? [he asks his wife] Damn, you don't remember anymore either.

Cristina. Six or seven years right?

E11. Or ten, fifteen.

Mar. And since then he has been on crutches

E11. I was going to rehabilitation, the doctor took me (….) Who treated my wife for some of her paralysis and every fortnight she would have her blood drawn and checked I took her blood pressure every day in the morning and at night and he took some pills that they sent him from XXX, from XXX or I don't know where. And then she [the doctor] took me and said "but XXX I will catch you" because that woman left me, just for that I already had a complaint and I did not report it, that I have not finished telling you and I am going to tell you why . Because I thought one thing and I have never been wrong, the one who reports is less, if I denounce that doctor they throw me XXX and my file that you will know that the doctors put a ponytail that they write in the History of each person, I have such a great history, a queue saying what happens and then where I go they don't look at me well, whatever hospital I go they look at me badly. When I told the doctor about this (….)

Mar. You say this because you think or know that in the history of patients there is a section for the doctor to put something negative about you

E11. Of course, the doctor who sees him already knows that I have denounced, that the one who is wrong is the patient and in the other way they have done everything I needed in XXX. I have gone to rehabilitation, and I argued with the doctor because they don't think I'm good where you see me [XXX laughs] It turns out that the doctor sends me (…) when she has me doing gymnastics, she sends me to rehabilitation and there I have to go every day, well, I go and on the second day he tells me "don't come tomorrow" I tell the monitor who is there "if I just want him to tell me the movements, I buy myself a bicycle and I learn it" and they tell me no. What I did, my wife came with a taxi every day to get me and took me. That day when I see a hole in the ground where I used to do it, I get on and he comes and he says “Hey, I told you not to come today, why have you come? I say “look then take me out; Until I finish doing the exercise I'm not going to get up ”she saw that I said it with bad temper, she still punched her and threw her against the ground she was close to her, yes, yes I take care of her. Then the doctor comes, as her name was, later she was a very good friend of mine, when the hen sings they are very friends of yours later, she says “hey”, tell me, doctor “the girl hasn't told you not to come today” and I say “ Well, yes, ma'am, you told me yesterday and I tell you as a doctor when you give medicine to the patient, when do you want it or when do you need it? she was white, she did not expect that, she says "when she needs it" I say "well, according to the doctor (...) who sent me here I had to come every day to do exercises," why can't I for me to do the exercises? " “No, no, yes, come when you want” and how do you know how the people in XXX are willing (….) To be happy, because they are happy that she was at the height of the shoe polish with me and the nurse that no longer he could approach me, not because I told him anything and they put a boy for me and he told me "you come every day and go wherever you want, wherever you want, do the exercises you want and when you get tired you can he " . Well, that's how I finished and when I finished it was when the doctor took me (….) And she has been with me well, [she addresses his wife] how long does it take you

Cristina. Well, a long time.

E11. Look if she knows her, she has very bad veins (….). In XXX they knew me even the cats and the doctors I know a lot and they have all gotten along very well. Well now when she tell you that I am going again and they say that I have the prostate again, but if I had surgery a year ago, how will I have it again? Well, they totally remove the lump from me and tell me "that you are going home tomorrow" I say "what do you say, that after being with the probe, now you tell me that I am going home tomorrow" I say " that is going to be with tomato doctor ”he says“ what have you told me “I say“ with tomato because I have come to remove the probe and leave, no, if I remove the probe and leave, I do not put the probe on, I will you take it away and if not, you take it away from me under my responsibility, I sign a paper for you, do whatever you want I have mortal spasms because to die it is not necessary to go home and I can die here I have had reasons "I know shut up and gave me the papers and I left. Of course, afterwards it has been difficult for me to return more times, they have had to send me many antibiotics, I have had to be in treatment for longer. Look at you, every 8 days I had to go, this girl came with me and to find a wheelchair there in XXX this girl has been walking for half an hour, you know that, there is a right to that, that I criticize it, one day she will I said to (….) I say "XXX, that's shameful" he says "what do you want me to tell you XXX" that I arrive right now and that there has to be a girl, a woman who costs me money and has to be the girl for an hour and not find a chair to get on the elevator to go to the floor I go.

Mar. If of course they are difficulties ...

E11. But many ... regarding the other here from the time I was, as I did not denounce as I said before to you, they have put all the devices on me and for having, they have put me in that drawer, in the ...

Mar. On the MRI.

E11. That and in others and they gave me a liquid, a medicine they gave me, beans and trials with me and I have suffered a lot but if now when a doctor stops to talk to me I say "look, do not get angry but I have many things done" . That I'm used to going to XXX and having a beginning girl that you see as soon as she arrives and says "hey, leave that, send another one that you've already gotten nervous and you don't know that they give me a bad tickle" however later they also did a blood gas test here, they did it to me that I did not find out and here they did it to me two women who were then on the twelfth floor who have already retired and said "oh XXX" I did not know, first they put the anesthesia, after ... 24`59

**E12**

Mar. He told me that XXX is unfamiliar

E12. No, not this lady. I was a hairdresser and her mother loved me so much, you don't know what that lady loved me. Dying in the hospital, "E12, E12 and E12" and a woman across the street said to me, "are you E12?", I say "yes ma'am" she says "always give us a few nights with E12 and E12", and I, who was her daughter ?, Well, XXX was her daughter and yet “E12”. The thing is that I had, I have, a way of being, very helpful, kind to everyone, I have always had a job with ladies and when she died [E12, XXX's mother] well… she had a family eh brothers, nephews, But since she had no money, no farms, because no one told her to take her [XXX] and as I was always with me at the hairdresser, I told her “well girl, look”. She lived in a little house that had a lot of water falling on her, very bad, damp, so I told her "girl then come, come with me" 1'09 and I have it now, well 44 years old. If when her mother died, the girl came here, well, girl who is already 80 years old and I am 81 [laughs]. So I have it, she has her little soldier because she has worked, she was a little short, silly no, but short.

Mar. Some alteration, illness means.

E12. Yes, she has a disease similar to her mother but, she is the other way around, as she gets older she becomes more intelligent. And we are the other way around, the older you get, we lose, because I am not a part of what I have been and she is not. Now she is taking more, she already understands things, before she did not understand the lyrics, she is advancing a lot, look so old, she already tells me things and I say "oh well, yes she has understood" before, no, nothing. However, I took her because, I already told her, (…) she is a good woman, of course what happens, she is very brave and makes me talk a lot in this sense, I tell her “Concha, when you watch TV please shut up”, no nothing, I finish telling her and that's it "E12, this" and I've been through a lot with her eh, I have cried a lot for her, not because she is a bad person, she is a good person but of course she was a little short, it is known that she did not understand that I was saying those things to her for her sake and now she is ill, she is delicate and yet I already tell her, as if she were my daughter, I still advise her and everything, but she, is what I tell her, is a a little like that and that's how we are. I shower her, wash her, take care of everything, all because she is lying down. She was like I was tall, like this [points to height] little and doubled with rheumatism, she has a bending disease, she has an ulcer on her leg that is 72 years old, since she was a child, she told me that, you know that they do verbenas and the parents were there near the botanist, a couple kicked him and, you know, the doctor right now, but in those days of the cuplé we didn't even have enough to eat because by the time they realized he had already been infected and was about to cut off his leg, he was saved, but since then he has had some little thing and ulcer on his leg, an ulcer is always like this, until now. So we went to XXX and a man, a doctor told me "oh, this is very complicated to operate" and I, of course as we do not understand, of course he gives us medicine and nothing. Later, we come back again and Don (….) Who is a flag surgeon, because we love him, she loves him as if he were her father, so Don (….) Says “like you have that leg like that” I say “uay , is that the doctor told me that it was very complicated ", he tells us" on the 9th here, to operate ", I said" but it has sugar ", he says" I will operate on her, come on the 9th "and there that the we go down. He made her a flap, removed meat from here [points to the area] from her thigh and scratched everything, down to her bone, look after 30 years. Anyway, they operated on her, it was fine, the woman has the flap still on and that but, her problem is that she has not yet touched the sheet like that, [claps] again, with ulcers and now that lady, We are in the hands of new XXX and those young ladies, the nurses of (….) come to heal her and that's how we are.

Sea Do they cure her? Have you not cured it?

E12. Oh of course, I have healed her for many years and she, but of course when operating her and everything, the wound is bigger and of course I am not, so the ladies come from here, the nurses, twice a week and they heal her and so on. we're. I shower her, in short I do everything, as if it were me… .5`56

Mar. XXX is she usually bedridden or does she get up?

E12. No, she gets up, walks a little because between her leg, age, rheumatizes her, because she doesn't do anything, poor girl. Look yesterday she scrubbed me and as if she had done a miracle "oh I scrubbed you", she gets up, washes a little, takes care of her little things, makes her bed, but nothing, just I'm the one who carries the whole matuta , everything

Mar. Are you E12 married?

E12. No, single, we both live here. She has already hit herself twice, she broke her arms, so she couldn't do anything, I had to do everything, dress her, comb her hair, everything, everything. She washed her hair, cut her hair, I told you, and I'm 8 1 years old, I'm not 20, uh, 81 years old, hard-worked, long-suffering, and starving a lot. You do not know but after the war we have spent ... and worked 24 hours without sleep, he spliced ​​day and night, he worked in the company of (...) on XXX street a German house, he was the German consul now he is in the conselleria, in short, I have worked a lot. Then from there I went to the houses, after the houses at 28 I was dying, I couldn't take it any more and I said to myself "it's over now" I'm not going to die anymore, I told my boss "Mr. XXX, if you give me something, I'll go "he tells me" from home I won't throw you away "," I know, but I just can't take it anymore. " It was a job there… with some wires, our fingers were cut off and working 8 hours like this [he does the posture, in a chain] at the station, we didn't even have time to go to the toilet to avoid wasting a little time, as it was at the station Well, of course, you did not earn anything, you will tell me ... and you know what I did, I was 28 years old I said "well, I'm not going to die" I took a bag, I put on combs, I put on curlers, I put on the fixes and how in the neighborhood everyone knew me, my mother was French, they called me the madame's daughter or E12 without, "Mrs. XXX whenever I want I will hook her hair" "oh well, look, come on Saturday E12 without", I did not know, of brushes or combing. You imagine the courage that I had

Mar. Very brave

E12. Yes, hunger did, man to make brushes, that I had never combed

Mar. Sure, it was something you didn't know

E12. Another day a lady called me, "I cut my hair" I say "yes ma'am" I thought "I will leave this one pelá" because look at what I knew about cutting hair, well, I was not so bad and that's how I started, but without a hair dryer big eh, I had a tiny one on my hand and before, you don't know, but the ones with long hair were servants who came from the villages, with tails, they didn't go to the hairdressers and one hair came to me here, another… .. With the little hand dryer "oh my mother" and my poor mother was a very fat lady 9'09 and said "lady have the dryer, I have the food" the poor thing had nothing, it was to sit down because she couldn't take it anymore. We have gone through what you do not know, but since then it was a clientele that was familiar, then I, look what I did to them, while drying, marking and sending to the terrace if there was Sun and wing "you see drying yourself"

Mar. What do you need right?

E12. Of course, if I didn't have the means and with the first little money I earned, then I already bought a helmet dryer and I was fighting better and then, well, I started to work in freelancers, I already paid and I retired, because I have cervical osteoarthritis and the bones then you will see how they are. I had some plates done recently and it hasn't come out, I have countless pains but for my age she says "it's not too bad yet" and that's how I'm living, I've already worked, I did a hairdresser, well, I just fought, 10'07 I am not going to die of that because if I continue, I do not arrive, that goes, nothing, nothing and I already started, I started, I got my professional card by myself, without anyone teaching me. I went to examine myself and I have the professional hairdresser's license, the tests that they did to me I got them wonderfully.

Mar. The experience you had

E12. But look, I was not a hairdresser, I was a brushmaker and yet I took it out, I remember that they asked me for a cocktail hairstyle and I did not wear a model and I said "oh mother" and I saw a woman who was a hairdresser and I I said (….) come, and she "girl if I'm not marked" I say, "you come here", I combed her hair, pulled out some highlights and passed the exam, so the need made me work, But because I have been very fighter and hardworking, because I had companions who had their 8 hours and were at home resting and were needy like me, but they work hard, not me, I could not be still starving. My father was also sick, the head of the family and father died at 53, sick, you know that nothing was charged or widowhood, nothing so I had to fight to the death but I say "I'm not going to die ”11`42.

Mar. Do you have a pension?

E12. Yes, very little. When I retired I did not know that I had so little, I retired due to osteoarthritis that made me dizzy and of course when I got dizzy I couldn't exercise and I couldn't work. So I said well, I'm retiring, I thought that I would stay a little pension because of course it was at 60 years old and it was also from when I quoted, but I did not believe that it was so little and it turns out that for one thing I had a bit of luck, I put in a little money, I took three million from the finances and then nothing, they have to take it away from me, well, what am I going to do and now I have to pay the government about five thousand money and I say "oh why, if They have already taken three million from me? ”, but do you know why? Because since I have so little pension I retired with a hundred I don't know how many euros, of course, now my pension is three hundred I don't know how many of course, no You can live with that, of course, I made a contribution, so the government helps me up to six hundred, so when I enter that little money the government also asks for its share and takes that money from me and now I have to spend 5 years paying 86 euros per month, Well, what am I going to do? Well, pay it, I have no choice.

Mar You don't have any help to take care of XXX?

E12. No, nothing like her has her salary. I'm doing a job that I don't have to do ... I'm 81 years old, I'm not 20, they tell me "E12, you could ask for help", because I do everything for him, I wash him, I cook him, I do everything for him. I as if she had a maid and nobody gives me anything, I have a salary, you see [silence]

Mar. And for now you can

E12. Yes, but in fits and starts, you think that at 81 years old and with osteoarthritis I can handle this all, what happens is that how do I leave it?, But I am here to take care of me but if I can not even lift the well and I have to do it, it is a very big responsibility I have to do it without being able to because I do not like to have dirty things and then I have to do it, but since I do not understand that, because I know that there are ladies who They give a helping hand for having another person to take care of them, but I don't, I don't know where those things are done.

Mar. And what do you think about taking care of XXX now? What do you think for the future?

E12. What do you want me to think? Well, have it until I can, [silence], as long as I can, I'll have it and that, I don't want to think about later. It has also been [cries] is that I did not think of anything ...

Mar. They are decisions that are made at a certain time

E12. I did good, without thinking [cries]

Mar. E12, get excited

E12. Yes [cries without consolation]

Mar. It is very complicated

E12. Besides, then [feelings, emotions, cries] he cut my life too, it is not the same to be in my house free than to have a person who is not.

Mar. And she doesn't have a family?

E12. Of course she has her, when I took her she had brothers, weddings and nephews.

Mar. But have they disengaged, or have they passed away?

E12. No, no, not nothing, nor did they worry about her, they did not tell her or come home or anything. Then as I saw that nobody told her anything about her, well I took her, if she had had farms, they would have taken her

Mar. Now, she doesn't have any contact with the family, and neither do you?

E12. Not me, yes, I spoke with a sister-in-law but since, as I told you, the family is not interested in having contact with her, on the contrary, when the nephews were little, yes, she went, but since now they have risen in category, the nieces work one in a bank because they do not relate, he forgot, and they have nothing more than her as an aunt, they have no one as a father, they have no one but her. Not one day a year, nothing

Mar. How has taking care of XXX influenced? 16`40

E12. Man !!!!, It is not the same, although I would not have done anything, because I have been a very homely girl, I have never been liberal at all. But hey, let's say I would have been free from her, well, I didn't cut her life either, but I didn't think of anything, I thought about doing her good and it's over

Mar. Do you have any friends now? Are you dating?

E12. Friends, we play Parcheesi.

Mar. But you leave home, do you relate?

E12. Yes, but more they come here, we play . Now because I am constipated and I am exhausted, very exhausted, but if not, then yes, you go out and go to a friend's house, what I can, but now I am annoyed. I have chest pain, it is that I have been starting for 15 days, it is a long time, I already told the doctor and he tells me "I have given him a very weak antibiotic" now I am thinking of going to a doctor that I know in particular because it is I'm tired, everything hurts ...

Mar. You notice that it does not improve

E12. I'm not getting any worse, but, I still start and everything, the color that I shoot is white that is not infectious and I have had a little fever, but I don't feel well. Since you already have what you have, spending 15 days making efforts because of course you notice it more than other people and I am exhausted. And I don't know what to do, whether to go to this man or what, he is very kind, very knowledgeable and to see if he gives me something, what costs me money? Well, look, it's the same, what am I going to do? Because if I advance a little, then it is always better, to be able to take care of it, because if not, do you see what plan I have? Who washes it, who fixes it, who [silence] everything? And I can't force it, don't believe the plan, I don't want to think about anything, but if it couldn't move, what would I do? I couldn't move it. That's how I am [cries]

Mar. I would have to assess what can be done

E12. But she is already like a daughter

Mar. Sure, you are fond of him

E12. She makes the gesture of a lot with her hands [she keeps crying]

Mar. People interact with each other and we love each other many times more than with the family, it is more the friendship that is made with a friend ...

E12. The family, the family don't even remember her, [crying], or at Christmas, one day, nothing at all. I would be satisfied if one Christmas day they said "XXX come", but no and she loves them but nothing.

Mar. Do you have an E12 family?

[She can't answer out of emotion, with her head she says no. We stop and I try to calm him down. When she recovers, we continue]

E12. My mom was French, with my mom's family nothing and my dad ... well my dad was from Valencia from a good family, he went to XXX with some friends 100 years ago and in 20'09 they just had to operate on a hernia, my mom I was a nurse and they met there, they had two children there, no, there were 4 boys and my father because of the disease, he had asthma, it was not good for him to be there and first he came and my mother brought me in the panchita, I was in 7 months, I was born in XXX. I am brought in from XXX and born in XXX. My brother, XXX who went to XXX with a lady with a girl and a boy and my brother, XXX, here single. It turns out that my brother from XXX was a healthy man, but he smoked a lot, a hard worker and XXX from here, was a piece of bread, better, not because he was my brother because the other one was more selfish, but this one was, Come on, we were both alone and of course he adored me, we lived very well, but in 15 days this one and the other will die. My brother got sick, the one here lasted 6 months and the one there, how he was from smoking because it is known that he ate the oxygen in the blood, he could not breathe well, he dies XXX and at the door he tells me “now I will die E12 ”[Cries inconsolably] 21'38 and that's how it was. My brother dies and after 6 or 7 days they call me that XXX is dying. I had to go to XXX [cries] and I'm alone. In XXX I have nephews and I cannot go there, I have already gone, I have only gone to XXX for grief, nothing more and now I ... I am not going anymore, whatever happens I am not going anywhere, no and that. They come sometime but they are far away, the money. Last summer they came and of course I'm happy and that's how we are, always waiting for them to come if they were here in XXX, I think so. I have nephews, nephews, little ones [laughs] of everything but of course not there, it's another march, another life. And I don't want to think anything, whatever will be

Mar. But you are worried

E12. Man, what do you think? I don't want to think about it, because if I thought about it, I would get worse, but they are things that I know are on the way, that's the way it is, you don't want to, you don't want to think but whatever it is will be. I live in the present, because if you don't make life more bitter and no, not me because I'm like this but I've been very happy I had a character that made everyone laugh, with what I was going through, hunger and everything, towards laugh at all the ladies. I used to tell the ladies jokes and they told me “oh E12, what a good character you have” thanks to that, it has saved me. Because here in front of me there was a hairdresser but that lady was very proud if you didn't go and she made you pout, I was the other way around, even if it took you two months to come, she told them "uy Mrs. XXX how are you" always so kind, they said " With E12 you can go, he always receives everyone with affection ”and that is what has saved my life, a very open character, I believe it if it had not been like that and that is how my life has happened.

Mar. E12 in the house with XXX they handle well?

E12. Woman, I can't, but I have to, you think that with my age, also with a general osteoarthritis that makes me dizzy, when I get dizzy I can't even get up

Mar. So what does he do?

E12. Now they have made me a badge, and in fits and starts, or I call a lady, pay her and she helps me and if not, what do I do?

Mar. Every now and then she has help

E12. It is that if I do not do, I can not. Look yesterday I came from XXX Street to get some plates because I have a pain and if I make a bad gesture, a gesture that is a little abrupt, they catch me like needles that are stuck here [points to the cervical area] I have to scream and everything Of the pain that takes me, because you imagine being as I am, because today she is lying down and I am cleaning the house. My television there also broke down and the man told me "I'll put it on again, although it's not fixed" I said no, sir, don't worry, leave it for me on the table, it doesn't bother me at all to make him work so that later I have to take it off and I have it there that's why the house is not tidy, but hey, that's how we're going, miss. Well, you know my history, take care of her and thank you that she has her pay because with that money I, with 500 euros, look at paying 90 for electricity, almost the same water, the stairs, the Sunset, because that month I do not eat, thank goodness if no, we couldn't be holy and that's life like that

Mar. Why would you say you take care of XXX?

E12. I took it because I am a very sensitive person and I feel sorry for everything.

Sea And now?

E12. [Cries] I would never leave her

Mar. Why do you think he would never leave her?

[Cries]

Mar. What do you think connects you to her?

E12. I am very sensitive [crying] and I would feel sorry for myself

Mar. Has she ever thought that she could go XXX to a nursing home, where she was cared for?

E12. Yes, but it would be when I could not attend to her, while she can not [cries], she makes me talk and everything, but you have her there. She has made me talk a lot shortly, but I will never leave her while she can, that's the force majeure, I can't [it sounds like] that it makes me have to blow my nose.

Mar. But that's good that way the mucus comes out, it's good

E12. It's already 15 days miss

Mar. As long as she needs her body

E12. But I suffer. I will never leave her while I can, when she cannot, she will be by force majeure, maybe it's me, maybe she lasts longer than me, she endures more than me. It is not known, it is not known. Look at the XXX coach, again to operate on him for cancer. That's why I don't want to think anything, because if I think I get sadder and so no, I turn on the TV and since I really like music and my life has been working and dancing, because I have worked like a black woman, I already know. I have explained and the only escape I had in life was music and I have danced very well, in movies, very, very well, so it is with the only thing that I escape

Mar. And now how does she escape?

E12. Well now I'm escaping in the retirement club, we friends went down but nobody knows how to dance, no, no. Look, they dance all the same and I have danced very well and then I hear the music and I get, I get more nervous, I like to dance, there is no one to dance, there are four couples, do you know what I mean? Since there is no one, then, or under, what for? They dance everything the same, they play a tango, they dance it the same, they play a chachaca, to everything the same and of course I have danced in movies, it is ugly that I tell you but I have danced very well. You do not see that you did not have money to go anywhere because in one peseta you entered and of course it was the only distraction and the first thing you went with the mothers there was nothing else in those times and my life has been that

Mar. Now when you have sadder days, what do you do? How do you get over those moments?

E12. Well, nothing, I call my friends, we start talking, we play Parcheesi, I have a big Parcheesi and we play games

Mar. Do you have support with friends?

E12. Yes, yes with friends, three or four are the same, then they go home and that's at least you're not so lonely all day, at least have some way out, if you don't notice

Mar. Do you have any kind of conversation with XXX? 30`49

E12. No, she doesn't have, XXX makes me nervous, you know? If not of course I would talk to her and of course I talk but she makes me die because I tell her one thing, she says it 20 times, I ask her something, 40, I tell her "XXX shut up and now I'll explain it to you" so I don't even I don't even know about the novel, I don't even know about the movie, I don't find out about anything and I am like that, I don't feel like telling you some things and then sometimes I regret it, “I'm going to send you to the room where you have your television there and I you will leave alone ”but then, I have her here by my side and everything passes, is what I tell her and so we go through, better times and more bad times. With the salary that I have, 31'45 because many ladies have told me "they gave me to help my mother, I don't know how many"

Mar. Have you requested any help?

E12. I don't know where I have to go, is that about social?

Mar. Talk to the social worker of (….) And she informs you.

E12. The girls who come see me. Since she has a salary, but it is not my fault, she has to eat, she has to pay the same as me, so I take the dead, I don't have to do all the work I do. In (….) There is a social one, in the street of the XXX there is that. Now when it's okay.

Mar. You have to go find out

E12. I am taking care of her for 40 years Do not believe that it is now, it is since I have it as it was short I have always been always, I take the house, everything, well I am. All my life I have it, I think I was 33 years old and I am 80 because I have it all my life.

Mar. Has the meaning of caring for XXX changed for you?

E12. No, no, I did not take her that day and always with the same love and always the same, because I took her because I wanted to, because it hurt me to see her alone and not with a very advanced intelligence and then I said "this woman ..." Well, no, he couldn't live like that because he is not a person who is well and you handle yourself. The girl to work and everything but she was short and I say "alone, the house as it is, a cold that was stuck there"

Mar. At first, from what you told me, did you take care of her for her mother?

E12. I took her, her mother loved me very much, she came to the hairdresser and I had a great friendship with her mother and the lady is that she was very good, and so that she was not alone. When her mother was there, she with her mother, but when her mother died is when I was sad to see her alone there and that is when I told her "well come with me" without thinking about it, or anything and it tied my life, of course, no Anyway, I was a very nice girl, now I will show her the picture of what she was like, a very nice girl and she cut my life, I did not think that I was shortened or that I was ahead of anything, I did it for good, I It was sad, I had my brother then, I was not alone, I had my brother and yet I put her in my house without thinking about anything and sometimes I have had them, of course. I had a relationship and because of XXX it was cut off, he told me "look at E12, I have no problem with you, but I can't stand XXX, you took her and it's you, but I have no need", it's not bad But, since she is deaf, she is deaf because sometimes she says something that, and I don't know what she said to the one that didn't sit well with XXX and then she cut herself off. She was a good person, an honest man, a hard worker, I entered her house, I knew the children all over the place, but I couldn't leave her abandoned either, look at me, I am like that, I do good to everyone I can. A lady comes and says "E12 leave me, what do I know, leave me I don't have to finish the month" I leave it to her, I have done a lot of good to everyone and they have made me talk a lot, I have lost many things and money and Everything for being stupid, for trusting me, it hurts me everything, not even that I was Sister Calcutta because yesterday I left her the money to finish the month, little that you have then wing ... ...

Mar. This gives you satisfaction

E12. It is born to me, it tastes fatal to me that this lady cannot finish the month, she has two children who do not work 37'23 and she says "I don't have a penny E12" you think that I, another says "well stop" and I, little what I have, well I'll leave it to you, since I've been through so much I know what it's like to go hungry

Mar. She puts herself in that person's place

E12. Man, I know what that is and I thank God I can eat and everything. I see that he doesn't even have enough milk, because I say "no, drink and when you can give it back to me" well, and I would tell him like this until tomorrow, I have done a lot of good to many people, to a lot, because I used to be at the hairdresser Well, I was earning more money and everything makes me feel sorry for me, is that what I say to you, they told me “it seems Sister Calcutta, everyone hurts you” and I killing myself at work and then….

Mar. E12, do you feel that these grants are recognized by those people?

E12. No, no, for some, I have only had two people, not two, one, who was married and the husband was a bullet, she worked, but of course with her salary, only the girl, like the husband, spent it badly or not he had and said to me "E12, leave this to me, when I get paid", the only person that I left him because I had that luck had a little bit and the only one who thanked me for it, I did nothing to make him thank me, because I Thank God I don't need it, I am a girl who with what I have I have plenty of everything, let's see if you understand me, the only one, because she bought me some wool and made me some bufanditas of those that were styled, so hairy and I He said "XXX you don't have to do anything to me daughter" and said "E12 you deserve it" and I "but no, no, I will do it to you because I want to do it to you, the poor girl" I didn't even have to finish, well, I had him to leave to finish the month and that's how it was going 39'21 so I would tell him until tomorrow, I'm like that but if I've spent more than San Amaro mpos, no one left you anything, and no one helped you because everyone was in need and I am like that. I do good without thinking about anything. It has to see that I have done that good without thinking that it could harm me, but I have done it because I am like that I cannot help it when I get angry I say "no more, no more" comes her niece "E12 let me have a boy ”we are the same again and I say“ no ”and then yes and I am like that.

Mar. You notice that XXX has some feeling of gratitude

E12. She says that she loves me very much, if she loves me, when I get angry or something she says "that she is like that, do not take it into account that I am like that, I love you very much" and why do you make me talk? "Ah, I'm like that" But she makes me talk and it's because she's a little short, she knows, being short a little bit, no

Mar. You excuse her

E12. Of course if not, she would not have it 40 years ago, but she does make me talk yes, I get angry at the moment but then nothing, she still makes me feel sorry, she does not see me as I have cried

Mar. She gets excited when she talks about XXX

E12. Yes, but I believe that God has always helped me, he has always helped me, he has given me a shame but on the other hand he has given me a window to breathe, always and that is true what I say eh, he has given me a It's a shame, a disappointment, I had depressions and everything but I have come forward [cries], I don't know, I always cheer on Him and I always move forward, for me it is very important I have always been like that, I tell you, I don't I do good because of what they tell me, how holy, but I am born to be and I have already told you I have many sorrows in my life, but also on the other hand it has brought me forward, I have seen other people who have not come out, do you understand me? And I am happy just as I am. I am not from mass, I tell you the truth, but I always believe and every morning I entrust myself and I do it because I am like that and I ask God to always help me and that's how it has been, it has given me a shame , a window has opened for me, I've had another pain, another window, that's how we've been playing and that's it and I'm happy, and I don't want to think about anything, or anything and go ahead if I cower, it's worse than me. Then, I start to clean the house as I can and I put on music and thus listening to music I don't remember anything anymore, she has to call XXX to put breakfast, while she washes I fix her and that's how the years go by, miss . She already knows my life

Mar. A very full and full life

E12. Yes, a lot and I have done a lot of good, you see, without being rich, but always, whenever I have been able, I have helped everyone, my mother, if I told you, it is that I am like that, at the same time I say I would have to change not being the way I am soft and that, because there has been a person who has hurt me a lot, a lot and yet I forgave him and helped him until he died, another says, if with the evil that you have done to me and that, nor look at them, I did not forgive everything. We cannot change and there I lost two million and a bit pesetas 30 years ago, which 30 years ago was a lot of money that I worked all my life and I lost everything and I even forgave him there I have the paper, if you want now I'll show it to you… .43 `32 [takes out the documents] and excuse me and until death I took care of everything [cries while taking out the papers]

Mar. But you are proud to be like this

E12. If not, I would not do it, look if years ago, 34 years at least, because 34 years ago two million three hundred and three hundred more than I forgave him [he indicates the name of the person and what he declares before a notary] I swear that he would pay me, me He would pay, because nothing and I even forgave him everything and he even lowered himself to me, he was dead, because I thought it was good for him, because it's the same. So you can see that I am not lying to you, go. And in 30 years two and a half million pesetas, 30 not 34 or 35 years

Mar. A lot of money, yeah

E12. And yet I served him to the end, huh. It was a family that, my mother, loved me very much, I took care of my mother-in-law, and he, well look, he was not a bad man, but no, he behaved very badly with me, but hey, it's over ...

Mar. How many experiences lived

E12. Look when he was younger, he left before, he did not pay attention to anyone, look what the couple he was with told him, "not like that", but nothing, he was a man that if you see him you fall, ua !, brunette , handsome, a boy, my dear, a good man, but what happens, they start, they start that if they smoke and that's it, and at the last minute they were going to cut off his leg and of course, they didn't [makes a money gesture with his hand] his little payment did not arrive at all and he called me "Duck" because he called me duck, duckling, because I walk like this [points with his hands, laughs] a little with my feet and I said "chicken, black chicken" as He was dark and then he called me and said “look at E12 they will surely cut my leg”, I said: “don't worry” 45`43 of course I didn't have money for a chair “I'll buy it for you” and he hears that I would even buy the chair. He tells me: [silence because he tries to control so as not to cry] he was very proud and he tells me "you are the most good woman in the world" [he cries with emotion, without consolation, we stop] I could already say it to. But I am happy, although he has hurt me, I forgave him, I live and I do not lack anything, neither to eat nor for anything, I have clothes I have everything, now what I have [laughs] is many years.

Mar. Nothing can be done there

E12. A man came when we were playing Parcheesi and he says "Hey lady, you want to change the gas, you want to change I don't know what" "no sir, no" "but you don't want to change" I said "yes, but change my age" he laughed . But hey look, I've been like this and I don't regret everything I've done, it's been a day, you see, why, no, man hI've been more miserable than someone else, but that's it. She already knows my life

Mar. It's a very interesting life. He has voluntarily cared for and helped people that often not even the family because of the relationship

E12. Or for the goods, for being a family, they are forced, they do not want, they do not want. Take her to the doctors at XXX, without being able I have to take her as if she were a daughter and you see. The passage of time makes a dent, if I was 20 or 30 nothing would happen, but I am older than her and I am, but well now God will make me feel good is that you have caught me at a time that of course as I am constipated no I don't feel like fixing myself or anything but when I fix myself and everything nobody makes me age or anything, I don't seem like that, uh! I'm horrible but come on when I get ready and I go cute little dress and everything… look, see if I have clothes I'm a very green grandmother [in the living room she opens some closets and shows me all the clothes] look at this blouse…. look, look [she is taking out pieces of clothing] see if I have clothes

Mar. But what closet has E12

E12. Look, look, everything is full, up and down and apart in other rooms, since you don't break it and I'm very capricious because she bought me [takes out clothes] a little thing in the middle of my possibilities, look, look, you see? three little suits and I have everything and there I have the XXX thing, the closet is full, do you know why? Because as it does not break, you see, look at what dress, look, because you have spent so much, as you have spent so much, look I have a friend who has been very hungry so she has…. gluttony, how do you say?

Mar. Craving to eat?

E12. That. Nothing else comes, he tells me "I'm going to do that, I'm going to eat this" I tell him, "but change the conversation" and I, the clothes, I the clothes, as I only had a little dress because I came out with wet sleeves and I " Oh my goodness, no one touches my arm ”Each one of them has had, he has hit me for my clothes and her for the food. I used to go every Sunday to the convent of XXX because I lived across the street, every Sunday for a year, so that they would give me four meters of fabric to make a little dress, every Sunday, all afternoon there without going out so that they would give me four meters of cloth, what do you think if we have passed? 50`00 Well, that's why I tell you that now, I do what I want and with my means because I don't think about anything and whatever it is, it will sound, because if I have to think that if it gets worse or that if it does not get bad, no , no, I don't think anything, so if I've bored you, I'm sorry miss, but that's how my life has been, I can't tell you anything else, [laughs] I've had good times and more than that. Now I am going to show you the photos and you are going to see…. [Goes out and brings photos]. Will you say, is that E12 ?, my mother, poor me, E12, who was going to tell me, of what I have been, but hey [bring more photos] and in this one I am also very beautiful eh, I am also very well and in this one you will see me, look, with this face and lock me up for the lady [referring to XXX] ……

Mar. Geez E12 is that she looks like an artist

E12. Well, that's when I took Doña Flor [he refers to Conchin] I closed myself in life. How old was I there? Well 30, when I took her and how pretty she was

Mar. Ava Gardner appears in this photo

E12. And how pretty was she? And I closed my life because see if I had admirers and I had everything, because I closed my life for her and I did not think about anything, I felt sorry and I took her and it's over, that's it. Look what face he had

Mar. The truth is that it is a photo of those of the actresses of the time

E12. I'm not so good here because this is a photocopy, but in the original I have it, you'll see, I'm going to get it, now you'll see. From E12 there is nothing left

Mar. The years go by and of course it has to be noticed in the physical

E12. You know what I'm telling you, that even when I fix myself I see other ladies my age and they are even worse than me 52'01. You see, this one looks more perfect, look at what hair I had, I used to comb my hair huh.

Mar. She is very pretty.

E12. She was one of those attractive faces.

Mar. But you E12 now don't look your age either

E12. What a saint and that you see me like this, but if you see me fixed, painted that painting favors me a lot because I had my job, because when I go painted and fixed and with a cute little thing. I went to (….) One day and he told me "Madam up to 70 years old, no ..." I say "until I'm 70? Well, if I'm 80 years old," he says "oh, well, God may keep her like that". Anyway, so I have had a very sad life and on the other hand happy, as I have done good to everyone I go to bed very calm, I lie in my bed so calm I say "oh what a rest" do you know how beautiful it is Is it going to bed with a clear conscience? What have you done well? What have they paid you badly? Well look, bad luck, but my conscience is very calm and I always have the illusion of living and of everything and of making myself beautiful and of finding a boyfriend [laughs]

Mar. Thank you and the recording ends. 53`29

**E13**

M. Carmen, since when have you been taking care of your husband in this situation?

E13 Well, look for many years, because my husband has been ill for many years. First they cut off his toe, he had gangrene and I alone with him because the boys are working the truth and of course they do a lot now with the work as it is, thank God, you know, a woman comes an hour a week and also I have a boy who comes for two hours, because my husband has to go out a bit because I see that he is doing well. If you ask him, I have to ask him a lot of things because he remembers everything more than I do, I am about that [memory]… you know, I don't do any at ca, but… .1'15, a lot happens to me little things and it's been many years now. Then a heel ulcer that the nurses took almost two years to heal, she did not heal, they tried everything. A nurse came who is in XXX who is very good and came to cure him and said "come on, E13 we are going to change him and see" (... ..) and it turns out that it took a long time and that is how we are almost, will we be 20 years? [Ask husband]

M 20 years XXXX in the wheelchair?

E13 No, when they cut off his leg, what were you doing XXX? I do not remember

M Don't worry ...

XXXX My leg was cut off this year.

E13 Face I would take him and he would get up and take care of his needs and face everything with the walker, he would walk around the house, which we also bought in (…) when he faced he was running and I was behind, always behind, then making my food, buying and my daughters also poison sometimes. I have one in XXX working there, when he finished his degree she went there and there she is, look

M What has two daughters? And they are in XXX

E13 No, one there and one here

M I had misunderstood him.

E13 Telephone, what I say, I am going to unsubscribe the telephone, you are doing one thing pum, pum, pum the phone tells me "why don't you pick up the phone?" Well, look why I couldn't, like this, like this the years have passed and look here we are. Then they cut him off, last year….

XXXX This year.

E13 For San José, they were also treating him and at the moment it was also done and they had to cut his leg, look and the other has it, touch it, when he left XXX, he had a wound on his big toe but there he has it and is adding to it. The other day he went to XXXX and they had to treat him every day.

M Do you cure it?

E13 Yes, I heal it

M Have you always cured him?

E13 Always, always, because this first one, the nurses here, who can ask, they came every day, as it was so long because they go day in and day out and face go once a week "heal it so, do it like this and like this ”come on and that's how I am.

M I understand that they have taught you to heal?

E13. Well yes and always looking clear.

XXX. There are many locks there ... [Silence]

Mar. What do you mean by bolts?

XXXX. Clumsy

Mar. Ah, the nurses?

XXXX. Yes

E13. No man no

Mar. Maybe he doesn't like how ...

E13. When they prick him they come to draw blood to do the control and everything, because there are some that come and immediately bang and others start and start and he says that this is bad, how they annoy him, but they are good people 5'28

Mar. But have the nurses taught you to heal or have you learned over time?

E13. They told me how I had to do it, "look first, she washes it in this water" that I have a little bottle there, it doesn't fit, "she washes it and then puts this insulin after this ointment", whatever it was and I It is not a thing that washed it well either, I washed my hands and I heal it and so I continue to heal it, every day

XXX. But nothing that follows.

Mar. That wound is still there

XXX. But it is not a care wound, no. But what happens is that this leg is the one that I have always had a little clumsy and it is the one that has remained, I resented this leg, the other one was good, but they made me a transfer there and a….

E13. They took a lot of fun to send us, you know, a little bit, because when I remember that finger that turned black, there was that woman who cleaned me and I said "come and see" her foot was in the water and she washed it "come and You will see my husband's foot "I didn't say anything to him" it seems that he has it purple, right "and that lady tells me" oh well, yes, he is right, he has a little "that was in the morning and at night. He had it black, see if it was quick and we immediately took him here and on his leg because they entertained a bit because he was telling them that he had his leg, that they were going to cut his leg "street seems like he wants to" and I told him man do not say that for the love of God do not cut your leg and look 7`42

Mar. E13, what do you do on a normal day?

E13. Well, on a normal day, Saturday and Sunday I have it only I have to get him up and go to bed, by the way I pick him up, he shaves the little sitting with the razor, he washes himself, the day before we shower him with the guy who comes, the Boy it goes clear and on Sunday I pick it up I put it here and I go doing things, the beds, the food and at 10:30 hours we are s the two of us sitting there listening to mass, every Sunday of the year and then later, not in the afternoon I have more free time, in the morning I am going to buy something that I forget as I can not carry it because I can not carry it and that in Just in the afternoon if, after eating I wash and we sit there, reading or watching television, until the evening to give him dinner and that's it, I make dinner. We eat at 1:00 p.m. or 1.30pm and dinner at 8.30pm. And so I wash, I leave the four dishes that are there and to bed.

Mar. Do I understand that one of her daughters lives here at home with you? Or not?

E13. No, no, we live alone, that's why I was very scared the other day because I fell recently in the elevator, a boy picked me up because I can't get up, I tripped in the elevator, look, it's just that I was a little high and I took the letters from the mailbox and I was looking at them and so I hit the ground, well look at this arm I have it facing badly that I went to Alboraya street last week

Mar. Were you able to get up?

E13. No, no, no, I got up, a boy who came down and lifted me and here I fell and I said "oh my lord, my husband cannot call anyone if he is not loud."

Mar. But E13 this second fall, has it been recently?

E13. Here in the kitchen and I fell, because I did a walk I do not know if I got dizzy, I got dizzy or I don't know what happened to me and I "bang, bang, I fall and bang" to the ground and I pull up and knees that I have them very bad

Mar, were you conscious?

E13. Yes, yes and I caught myself, I opened the washing machine, I was able to kneel a little which I cannot because it hurts a lot I am to operate, this arm I cannot lift it anymore [makes the movement] to comb my hair look, I have to comb my hair in this one and this neither, look

Mar. And what did he do?

E13. Well then little by little I put one foot like this [he does the posture] and little by little I made an effort and I was able to get up.

XXX. She leaned against the hole in the washing machine.

E13. I leaned so hard and it cost, it cost me but finally, do not believe that it was and I got up

Mar. Was she alone?

E13. Yes we are always alone, my husband was there but he could not hear me, he was in a little room that he has to read and even if I had called him he could not help me, well to give voices to the neighbors, but it took me almost half an hour, and little by little thanks to God and here we are. And the children who also have children, have their problems that many have and look …….

Mar. Do children help you?

E13. Yes Yes

Mar. What do children help you?

E13. Well, look, this one who is here, is the one who goes to XXX with her father and then when I go to the doctor she comes with me, to the doctors everything, everything is with me this girl who is here that if my husband and me she accompanies us .

Mar. And then she has the help of that boy to lift him

E13. To lift it, lay it down. Before, since everything costs a lot, I came at night to put him to bed on purpose, he came for a long time but things are happening and I can not do everything

Mar. Do you pay for this? Isn't it a resource or aid under dependency law?

E13. No, nothing, nothing and the woman who comes once a week also pays for it myself and of course I can't and this boy came up with putting a long this on the wall on the wall and my husband does like that [he pretends to take some of the wall and he stretches] and he grabs and helps himself, he gets up I put the mattress on his ass and he sits there and on Sundays and I can handle everything. When the boys come, they come one day, well they help you, but they always come running, what I say always running

XXX. Doctor visit can be said. Except for this one who is here and accompanies us to the doctor [silence] 14`12 because that's what there is

E13. That's what I say, as long as we can use it like that, because I once said it to the nurses who call me from XXX "E13 we take him [to XXX] somewhere" I say, no, while I can, I don't want him to go to nowhere my husband, I take him home and I will do everything I can until God wants and I can help them [cries] because sometimes God knows. He is very well now, they did the analysis of the syntron and he is quite well. This is what there is, there is neither more nor less [silence] He remembers more of the things that I [cries], he is 88 and I am 86 years old, it's been many years now [silence with great emotion, XXX goes down head and cry]. I manage as well as I can and he has fallen on the ground many times and with a short leg I fell another day, he fell asleep a little and fell and I called the neighbors and they came to help me pick him up

Mar. Can you count on the neighbors?

E13. Oh yes, they have already told me many times, you call me right away. Another time, he fell in the kitchen, we were going to have dinner, one of them came and says "I just can't pick him up" because he has something too, he now knows that I can't, but before he fell, because he has fallen a lot of blows , a lot because he fell down and said "don't call anyone, no, come here and help me, help me" [with a high and authoritarian tone] and of course I couldn't and said "XXX, I can't" well look, that's how we are and that's how it goes, while I can, we'll be here.

Mar. How many years have you been married?

E13. Huy I no longer remember, 60 and I don't know how many

XXX. 60 years made in February.

E13. We got married in '52.

Mar. I ask you because you said that they told you at the hospital that XXX should not be at home?

E13. You can ask the nurses, what is the name of the one that comes from XXX ?, the one that calls you on the phone….

XXX. Mª Angeles.

E13. She's the one who told me, that's when they already cut off her finger.

XXX. She is the one who takes the chronic and calls every 15 days, she has that occupation not only to me, to many. The other day he told me "I'll call you in three weeks" and that's because "because they have already cut us off, the crisis" he told me so every 15 days now it will be every 21 days, you can see the mismanagement and fraud that there is behind

E13. It seems to my son that he found it funny but no, no, the father is coming home and I want to have him here while I can.

Mar. Do you have another child?

E13. Yes

Mar. Two Daughters and a Son

E13. Two sons and two daughters, the other one died, look at her, that's the one [shows the photo in the dining room]

Mar. And the two children also live here in XXX?

E13. Well, look, yesterday someone came, he called me at 6:00 p.m. "Mom, I'm going to go" I say, now at 6:00 p.m. "no face at 7:00 p.m." and at 8:00 p.m. he had already left "to see how you are, to be a a little while with you ”and the other, since he's not here, he's in XXX, he's a doctor too, because that one, you can't count on him either. When he comes, well he comes one day and leaves 18'52

Mar. As you say, doctor visit

E13. Doctor visit, well yes, everyone is working, thank God but everyone has their problems because they sometimes come and face me, they tell me, the one from XXX tells me "Ah, I will not reach your years, mom, I will not arrive" because it will be God willing, look

XXX. You'll get by [laughs]

Mar. Many times we children do not realize what our parents are going through, we think they are better and it turns out that they are not.

E13. Do you know what I answered? I say, I no longer remember what I was going to say, sir, look at ourselves we started and alone we are finishing, oh yes, I already remember what it was, she told me "I will not arrive "I say then it will be what God wants and I would like you to arrive to know the disappointments that are received, that they have [cries, XXX looks the other way] and they are good, I have nothing to say about them because, they are good, they have never They came drunk, nor did they hit us like those who kill their parents, no, but I wanted something else….

Mar. You are alone, unattended 20`28

E13. Yes, they say, this one says "mom, well, if I… .everything, everything" and the one from here, if I call him, he comes right away, hey, I call him and he comes right away, everyone, every day of the year they call me, but I I say, what for I am so much ... "how are you?" if it's always the same, well here we are, what are you going to say, fine as always and it's over.

Mar. And tomorrow more ...

E13. That is tomorrow more, and this is what it is.

Mar. Do you have grandchildren?

E13. Yes, I am 6, all boys and all older, yes, the oldest will almost be 40 years old.

XXX. Well 33 or so he has.

Mar. How about the relationship with the grandchildren?

E13. Well, well also on the phone, when they call.

XXX. The elders do not call, the only one of this one [points to the photo] who is near here comes and the one from Tarragona who is the little one when the parents come approaches

E13. This is coming

XXX. Well every month they go around to see how it is, because it is more related, the others have gotten older and not even see you revere. When my children come I don't even ask about them because they are old enough to ask and worry about their grandparents and they don't do it because they give them blood sausage [E13 cries and XXX shows irritability]

Mar. Of course with those ages they are already adults

XXX. To take what little is left if they will be ready. Children, grandchildren and sons-in-law and daughters-in-law, eye

Mar. Do they move easily around the house, with the chair?

E13. Yes, yes and in the bathroom I also have one of those chairs to bathe him, but since the boy is strong, he seats him and he does so. You have to get used to everything, everything. I have never been sick and neither has he, since he started with diabetes but we have never spent, is that no, you had a cost and anyway you were cured and you did not go to the doctor and na, you know? And the children, thank God too, they have grown up healthy but he comes to work, Sundays and everything, day and night and I do the same.

Mar. Have you worked outside the home? 23`43

E13. No, but look [he speaks with great emphasis and authority] I have had five children and I have never, never looked for anyone, I have never looked for anyone, I have done it at night or during the day I have done it to myself as I could , but nobody has ever come to my house, so now I find nothing well done. I say Lord close my eyes and let it pass, what are we going to do, is it like this, like this

Mar. Did you take care of your parents?

E13. Yes, my father, we were already married, yes. When my mother was single, my mother had high blood pressure, when it was very high a practitioner would come and draw blood from her, look at what way to heal us, they took two little attacks, she stayed a little and then recovered, I remember that she took the leaves of the olive trees to lower the tension. My father has never been, never sick, but they told him not to smoke, mare bought him a tar cigar that they used to sell, now there won't be any of that, it was a cigarillo that tasted like tar. He was 8 days without smoking, after 8 days he was already smoking in hiding and told ma mare "look after lunch I will smoke a cigarette" [he laughs, it is the only time I see him smile], and ma mare said "well "In the end we also left it as impossible and from that he died, he developed angina pectoris and he died. But [she raises her voice angrily] has never spent on doctors either 25`53 has never said my father "I'm going to the doctor" like now everyone goes to the doctor of the balls, [very angry] everyone has something, I have never I heard my father go to the doctor, have you ever heard him?

Mar. It is that before he lived differently ...

E13. Well, he has never, never been to the doctor. The time it was, where XXX was not there and he died at home where the doctor came from XXX that I was from here from XXX and the doctor came running, it was my husband with a bicycle and when he came he said "there is nothing to do" and caught him in the car, thank you that he got home. Most of my father's family died in the fields, one was watering, he stayed; another, he too fell dead right away, like that, like that everyone. But to the doctor, never, my father went for that so that he would stop smoking and he did not quit, but he has never gone for a cost or anything, nothing. His kidneys ached, he said, he put on his girdle tightly and went to work in the fields, well, that's how it was. And neither are we, and I am Margarita [the assistant], she tells me "E13 because she complains if she is in the best of times" and I say: of course we are all fine visiting. They do an analysis a year "look at E13 everything has gone well for you a little bit of cholesterol" it came out the last time, he did not give me pills or anything, well look. I have had the children, see if I tell you, I had the little one at 3 in the afternoon, my husband went to get them from school and when he came I got up and went to give the children dinner. I got up, made dinner and I was there with him, you see, in the afternoon at 9 o'clock at night he was in the kitchen and he was born at 3 o'clock, you won't believe it but it's true.

Mar. Of course I believe it, you tell me and I believe it, I smile because today ... 28`48

E13. The one that cost me the most is one that did the sesarea for life or death, the first sesarea that was done in XXX, so the midwife told me, then I had insurance and paid more than medicine, the doctor did not say "You already have the insurance paid" for the whole year, for my whole life, that the year, I had a cesarean section. They took me home after 3 or 4 days and right away to work, if I've had a lot of calamities, but why? Now I see everything that happens, I have never been a boyfriend anywhere alone, we always had company. We were 7 brothers, 4 sisters, the oldest was me and here [points to the place] another was going who was almost my age, we got along little I see the things that are happening and ... not so much not so little that is my idea eh, because Now I believe that women do not know, I do not know how to say it, to respect or to keep their honor. Now they are sold daily, today they go with this one and tomorrow with that one and that's why those things, no, no

Mar. Do you have any friends? 30`25

E13. A sister that I have left comes to see me another from XXX who comes who knows us

XXX. Here in the neighborhood we have lived since we got married and here on this farm, it was a trash can, the palm tree that is on the door was inside the trash can, so the street of XXX was, well, half or the third part of what is wide and there were two directions, with old cobblestones. In front of the field, this is the paper mill, there a factory of boards and all orchards. We lived in the group of arches, we have lived there all our lives and we know if there is any old man around here from the neighborhood then, we say of the group because they are the ones we know and people, the neighbors, because when they started to build this farm they already We stayed on this floor, when the foundations began we already started to pay and of course we are among the first to come and we meet some, but not from visits to each other, each one at home. I think I have not gone to anybody's apartment here, nobody's, we know each other from the stairs but nothing else.

Mar. I ask you because E13 has said that you go out with the boy every day, do you E13 go out?

E13. No, I do not go out, well to buy or if I have to go to the doctor's office, some slip, I go into my house and it is already fa years

XXX. Visiting and that nothing.

E13. Before, do you know where I went every day, even though it was the little kids? He sent them to school ... to San Nicolás, on Mondays to do the novena. Since I have not been able to go well, He knows, but I do pray to Him. I used to go with my sister, the two of us together, because that's what I used to go out all my life, I haven't had Sundays, or parties or at Christmas because the children used to get together and we and look at them there you have [points to the photos] everyone, we joined at home and we didn't go out anywhere, we spent the parties at home and at. 34`01 now is something else ...

Mar. E13 Do you feel that you are thanked for what you do? For example, his husband, even if he is here, do you feel grateful for what he does to him or to the children?

E13. No, no, no, no, no, no, no, me, my husband tells me "bring me this, or the urinate bottle" or "drain me water" and right away, I leave what I have to do and I first things first he. And they, well the same.

XXX. But she doesn't ask you that, [she tells me she] "she is deaf, with one ear nothing, for years and with the other she has worn a device, but she hears what she hears"

Mar. Well E13 yes he is talking to me, continue the conversation

E13. Yes, but from this one [points to ear] I don't hear anything, nothing

Mar. I understand perfectly what she is telling me and she also understands me

E13. I've already spent 2 or 3 of these

Mar. Gadgets?

E13. Yes, look at those photos, both of you young

In January, we were single and in that [points to another photo] we had been married for 50 years, look how good we were there, it was the same date.

Mar. What I'm going to ask you may be silly. Have you noticed the passage of time?

E13. Well, look, I have not realized it, so many years have passed, I have not seen them count, no, not that they have happened quickly, sometimes, before my girls when they went to school, I was already 50 years old and I pissed " Mom, XXX always asks me how old you are ”tell her 38 and she answers“ my mother told me that she is 38 years old ”, she said“ 38 years old your mother, she would like that, she will be at least 50 or throw up "

Mar. The thing is, now you don't look how old you are and neither does her husband

E13. But here I am, look at these two [my knees], they told me to operate on both of them, this arm is useless because all the strings broke, from here I cannot lift it anymore, look, [makes the movement and actually does not have the bow of full movement]

Mar Why is it not operated?

E13. Because I don't want to [angry] here [for my arm] they told me that I had to spend 4 hours in the operation and that they had to put me here I don't know what and they didn't give me guarantees, I say no, as it doesn't hurt. But this one [points to the other arm] hurts, of course from the blow that I fell in the elevator because a month later I went to the doctor to see and he told me to get an X-ray and I had it that I had to go to XXX and they told me that It was very bad, I had I do not know what, the truth is that they brought the result about a month ago and they told me that there is no solution this is artocis. When it hurts me very badly and here also in the legs, they told me if it hurts a lot, he goes to the office to give him anti-inflammatory pills and nothing else and that's how I am. With both fingers [he shows me the fingers of his hand, a malformation is evident, important] I have been a season when I could not sleep in bed these two fingers hurt a lot, I went to the doctor, my daughter says “I'm ashamed, afterwards, you don't want to do what they tell you ”. They wanted to operate on both fingers and I said "I do not have surgery" and they did not operate on me. Here [he points to his arm] they told me no, but here [he points to his finger and knees] yes and I know what he did I stretch them and with a ball [he does the exercise], I had them very bad, now they fall asleep , so, many things but here I am [silence] 39`40
